# Geminal bis-borane formation by borane Lewis acid induced cyclopropyl rearrangement and its frustrated Lewis pair reaction with carbon dioxide†
†Electronic supplementary information (ESI) available: Experimental and analytical details are given. CCDC 1487577–1487584. For ESI and crystallographic data in CIF or other electronic formats see DOI: 10.1039/c6sc03468c



**DOI:** 10.1039/c6sc03468c

**Published:** 2016-09-16

**Authors:** Yun-Lin Liu, Gerald Kehr, Constantin G. Daniliuc, Gerhard Erker

**Affiliations:** a Organisch-Chemisches Institut , Universität Münster , Corrensstraße 40 , 48149 Münster , Germany . Email: erker@uni-muenster.de

## Abstract

Cyclopropylacetylene reacts with two molar equivalents of Piers' borane [HB(C_6_F_5_)_2_] under mild conditions by an addition/rearrangement sequence with cyclopropyl ring opening to give a mixture of two α-B(C_6_F_5_)_2_ substituted tetrahydroboroles. This compound forms an active frustrated Lewis pair with P^*t*^Bu_3_ that heterolytically splits dihydrogen and adds carbon dioxide as a geminal chelate bis-boryl component. The respective reactions of the two-fold HB(C_6_F_5_)_2_ addition to Ph-CH_2_CH_2_C≡CH were studied as a geminal Lewis acid reference. Most of the products were characterized by X-ray diffraction.

## Abstract 2

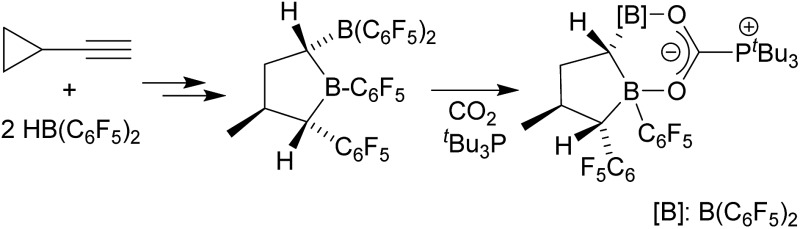
The borylated tetrahydroborole obtained by the reaction of cyclopropylacetylene with Piers' borane adds carbon dioxide under frustrated Lewis pair conditions.

## Introduction

**Scheme 1:**
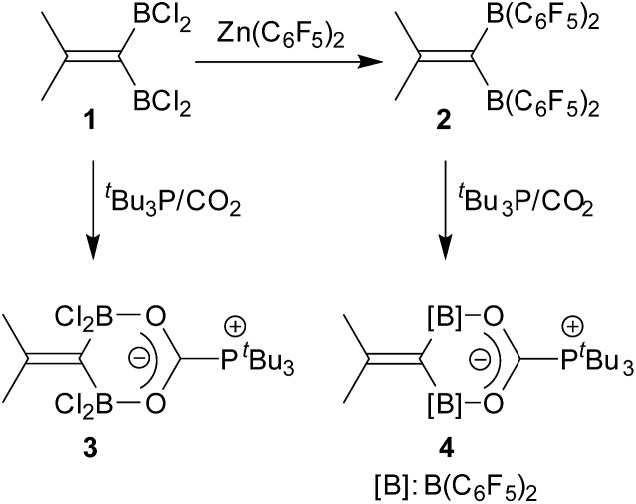
Geminal bis-boranes and their FLP reactions with CO_2_.

Bis-boranes featuring pairs of strongly Lewis acidic B(C_6_F_5_)_2_ groups should be ideally matching templates for binding of CO_2_ under frustrated Lewis pair (FLP) conditions. Although such geminal bis-boranes are principally readily available from terminal alkynes by sequential hydroboration reactions with two molar equivalents of HB(C_6_F_5_)_2_, as it has been shown by Piers *et al.*,^1,2^ surprisingly little is known about this CO_2_-trapping reaction. Stephan *et al.* had used Siebert's unsaturated geminal BCl_2_ compound **1** (ref. 3) and the corresponding B(C_6_F_5_)_2_ analogue **2**, which was derived from **1** by treatment with Zn(C_6_F_5_)_2_, for FLP/CO_2_ scavenging,^4^ but the vast majority of FLP/CO_2_ chemistry used non-chelate Lewis acidic binding motifs^5,6^ (Scheme 1).

We have now investigated the ^*t*^Bu_3_P/CO_2_ trapping reaction using a pair of geminal C_6_F_5_ containing bis-boranes. Both were obtained by the treatment of the respective terminal acetylene starting materials with two molar equivalents of Piers' borane [HB(C_6_F_5_)_2_]. While we observed the expected normal behaviour upon reacting the alkyne Ph-CH_2_CH_2_C≡CH (**5a**) with the hydroboration reagent, we observed a rather complex rearrangement behaviour that took place upon the treatment of cyclopropylacetylene (**5b**) with the HB(C_6_F_5_)_2_ borane. The characterization of the resulting special rearrangement product, its formation and its FLP reaction with CO_2_ in the presence of a *tert*-phosphine will be presented and discussed in this account.

## Results and discussion

### The Ph-CH_2_CH_2_C≡CH/2HB(C_6_F_5_)_2_ system

Terminal acetylenes undergo regioselective 1,2-hydroboration with the HB(C_6_F_5_)_2_ reagent to yield the respective substituted vinyl boranes.^7^ When the reaction is carried out in a 1 : 2 molar ratio of alkyne and [B]H borane, the respective saturated geminal bis-borane is obtained in many cases under kinetic control.^1^ This typical reaction path was also observed when we treated the alkyne **5a** with HB(C_6_F_5_)_2_ in a 1 : 2 ratio in toluene solution at r.t. (1 hour reaction time). Workup gave the product **6a**, which we isolated as a white solid with a 76% yield. The compound was characterized by C,H-elemental analysis and by spectroscopy, and we carried out some characteristic reactions.

Compound **6a** shows a single ^11^B NMR resonance at *δ* = 72.1 ppm, which is typical for Lewis acidic planar tricoordinate R–B(C_6_F_5_)_2_ situations.^8^ Consequently, we observed the three ^19^F NMR signals of the symmetry-equivalent C_6_F_5_ groups at boron. They show a typical large *meta*/*para* fluorine NMR chemical shift difference (Δ*δ* ^19^F_*m*,*p*_ = 13.7 ppm). The mixture of compound **6a** with the bulky phosphine P^*t*^Bu_3_ (1 : 1) represents a reactive frustrated Lewis pair that is able to heterolytically split dihydrogen^9^ under mild conditions (r.t., 2.0 bar of H_2_, overnight in pentane). The product precipitated from the reaction mixture and was isolated as a white solid with an 87% yield. Compound **7** was characterized by X-ray diffraction (single crystals were obtained from pentane/dichloromethane at –35 °C by the diffusion method).

**Fig. 1:**
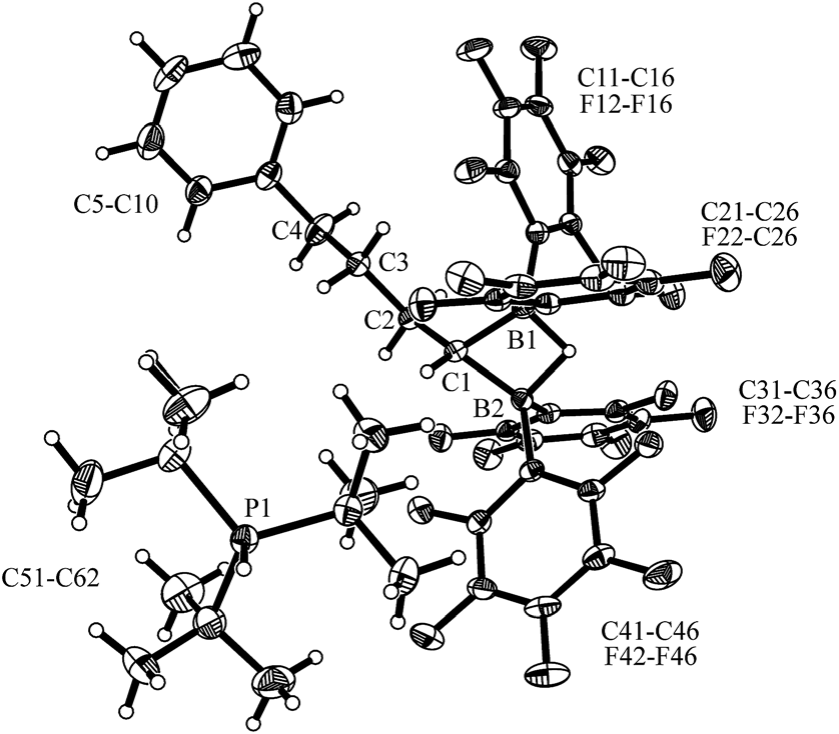
A projection of the molecular structure of the FLP dihydrogen splitting product **7** (thermal ellipsoids are shown with a 30% probability level). Selected bond lengths (Å) and angles (degrees): B1···B2 1.945(5), C1–C2 1.526(4), C1–B1 1.609(5), C1–B2 1.598(5), B1–H01 1.26(4), B2–H01 1.34(3), B2–C1–B1 74.7(2), B2–H01–B1 96.9.

**Scheme 2:**
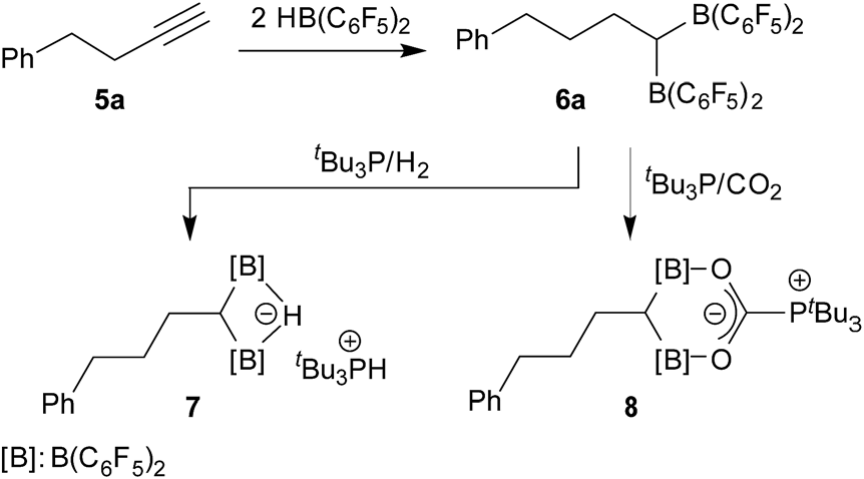
Frustrated Lewis pair reactions with the geminal bis-borane. **6a**.

Compound **7** shows a fully extended all *anti*-periplanar C_4_-chain featuring the phenyl substituent at one end and the geminal pair of boryl groups at the other. The C1–B1/B2 bonds are almost of the same length and the pair of boron atoms is bridged by the hydride (see Fig. 1). In the crystal there is an independent HP^*t*^Bu_3_^+^ countercation. In solution, compound **7** shows the ^31^P NMR signal of the [P]H phosphonium cation (*δ* = 60.2 ppm, ^1^ *J*_PH_ ∼ 430 Hz). The anion shows the single broad ^11^B NMR resonance of the symmetry-equivalent pair of B(C_6_F_5_)_2_ groups in the typical tetracoordinated borate range (*δ* = –18.8 ppm). The C_6_F_5_ groups of the boryl groups are diastereotopic. Therefore, we have observed two sets of *o*,*p*,*m*-C_6_F_5_^19^F NMR resonances for these units. The bridging [B]–H–[B] hydride gives rise to a broad ^1^H NMR signal at *δ* = 2.64 ppm (Scheme 2).

**Fig. 2:**
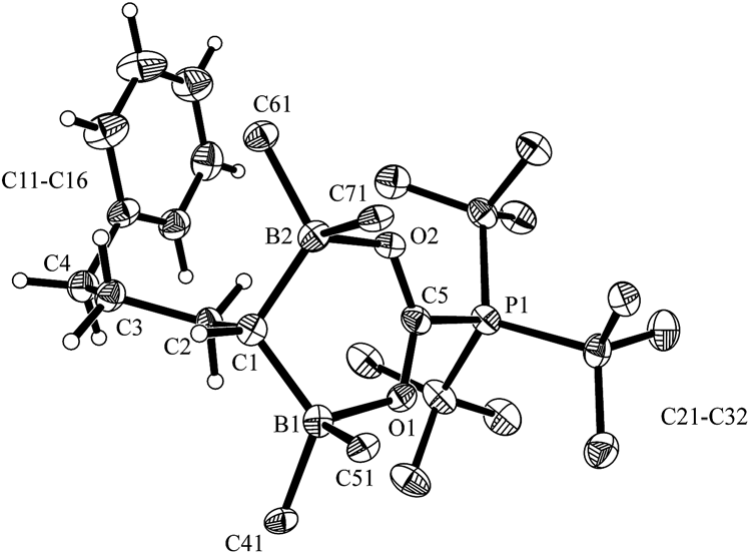
A view of the molecular structure of the zwitterionic FLP/CO_2_ adduct **8** (thermal ellipsoids are shown with a 50% probability level; hydrogen atoms of the P^*t*^Bu_3_ group and the C_6_F_5_ substituents at boron atoms B1 and B2 are omitted for clarity: for details see the ESI†). Selected bond lengths (Å) and angles (degrees): B1–O1 1.628(4), B2–O2 1.635(4), O1–C5 1.265(4), O2–C5 1.264(4), B2–C1–B1 108.6(2), O2–C5–O1 127.7(3).

We then treated the bis(boryl)alkane/phosphine FLP [**6a**/P^*t*^Bu_3_] with carbon dioxide. Exposure of the **6a**/P^*t*^Bu_3_ mixture in pentane at r.t. to CO_2_ (2.0 bar) quickly (in 2 hours) resulted in the formation of a white precipitate of compound **8**, which was isolated with an 81% yield. Compound **8** is sensitive in solution (CD_2_Cl_2_) and decomposed above 0 °C. Single crystals of the FLP/CO_2_ adduct **8** suitable for characterization by X-ray diffraction were obtained from pentane/dichloromethane at –35 °C by the diffusion method (see Fig. 2). In the crystal, compound **8** shows a *gauche*/*anti*-periplanar conformation of the Ph–CH_2_CH_2_CH_2_–CH-chain. The geminal pair of B(C_6_F_5_)_2_ substituents at carbon atom C1 has taken up the CO_2_ molecule in a rather symmetric way by forming two boron–oxygen bonds of almost the same length, and also the C5–O1/O2 bonds are almost equal in length, indicating a fully delocalized structure for this sub-moiety of compound **8**. The resulting six-membered heterocycle features an almost coplanar arrangement of the BOCOB unit with only the carbon atom C1 being localized markedly outside of this plane. The bulky P^*t*^Bu_3_ group is found attached at the central carbon atom C5 of this heterocyclic subunit of the overall molecular zwitterionic FLP/CO_2_ addition product **8**.

In solution we observe the ^13^C NMR resonance of the scavenged CO_2_ molecule at *δ* = 172.7 ppm with a ^1^ *J*_PC_ coupling constant of 92.6 Hz. Compound **8** shows a typical phosphonium ^31^P NMR signal at *δ* = 60.3 ppm, and a single broad ^10^B NMR resonance at *δ* = 10.8 ppm. The C_6_F_5_ groups at the pair of boron atoms are pairwise diastereotopic, giving rise to two equal intensity pairs of *o*,*p*,*m* ^19^F NMR features, with a rather small chemical shift difference Δ*δ* ^19^F_*m*,*p*_ around 5.5 ppm, as is typical for borate type structures based on the B(C_6_F_5_)_2_ subunit.

### The cyclopropylacetylene/2HB(C_6_F_5_)_2_ system: rearrangement to tetrahydroborole derivatives

**Scheme 3:**
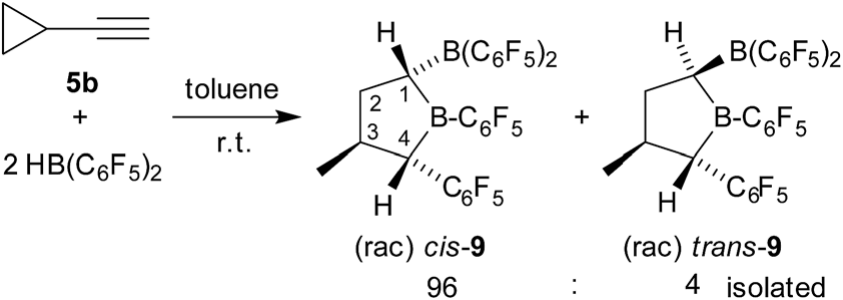
Formation of the tetrahydroborole derivative **9** (with unsystematical atom numbering scheme as used in Fig. 3).

We next reacted cyclopropylacetylene (**5b**) with two molar equivalents of Piers' borane [HB(C_6_F_5_)_2_] (toluene, r.t., 1 hour). In this case, we did not obtain the simple cyclopropyl-CH_2_CH[B(C_6_F_5_)_2_]_2_ product (**6b**), but found that a rearrangement had occurred. *In situ* NMR spectroscopy revealed the formation of a *ca.* 7 : 1 mixture of the α-boryl-tetrahydroborole products *cis*-**9** and *trans*-**9**. We isolated the compound *cis*-**9** in an almost pure condition (96 : 4) after workup as a pale yellow solid with a 67% yield (see Scheme 3).

**Fig. 3:**
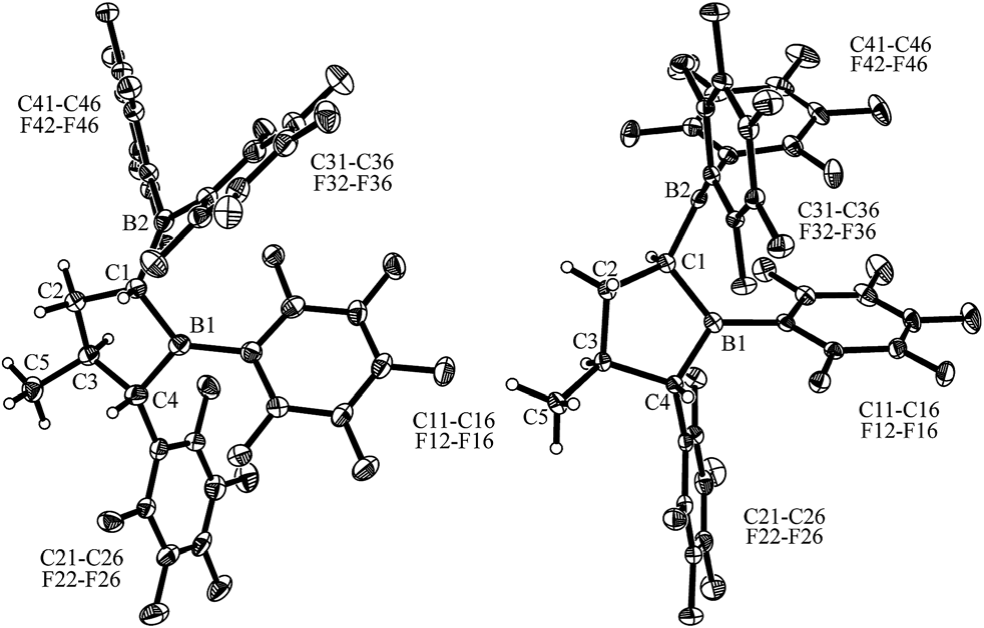
A view of the molecular structures of the α-boryl-tetrahydroborole *cis*-**9** [left, thermal ellipsoids are shown with a 30% probability level; selected bond lengths (Å) and angles (degrees): B1–C1 1.582(7), C1–B2 1.561(7), B1–C4 1.576(7), B2–C1–B1 120.3(4), C11–B1–C4 125.5(4)] and the *trans*-**9** epimer (right, thermal ellipsoids are shown with a 50% probability level; the separate synthesis of *trans*-**9** is described below). Selected bond lengths (Å) and angles (degrees): B1–C1 1.588(2), C1–B2 1.543(2), B1–C4 1.579(2), B2–C1–B1 114.0(1), C11–B1–C4 124.3(1), ∑B1^ccc^ 360.0, ∑B2^ccc^ 359.8.

Compound *cis*-**9** was characterized by X-ray diffraction using single crystals that were grown from a pentane solution of the compound at –35 °C (see Fig. 3, left). The X-ray crystal structure analysis has shown that a five-membered saturated tetrahydroborole framework had been formed in the reaction, bearing a B(C_6_F_5_)_2_ substituent at the α-position C1, a methyl substituent at C3 and a C_6_F_5_ substituent at C4. The pair of substituents in *cis*-**9** at carbon atoms C1 and C4 are *cis*-oriented; both are in a *trans*-arrangement with the methyl substituent at carbon atom C3. The plane of the C_6_F_5_ group at C4 is oriented markedly away from the mean heterocyclic core [dihedral angle *θ* B1–C4–C21–C22 –119.6(1)°], whereas the C_6_F_5_ group at the adjacent boron atom B1 is rotated slightly in the opposite direction [*θ* C1–B1–C11–C12 49.0(7)°, C4–B1–C11–C12 –136.2(5)°]. Both of the boron atoms B1 and B2 show trigonal planar coordination geometries (∑B1^ccc^ 359.8°, ∑B2^ccc^ 359.9°), which should render these both as strongly Lewis acidic centres. Consequently, we have monitored a pair of ^11^B NMR features for compound *cis*-**9** in solution (d_6_-benzene) in the typical range of Lewis acidic B(C_6_F_5_)R signals (*δ* = 78.0 ppm, 67.6 ppm) and a similar appearance of the ^19^F NMR spectrum was observed for the B(C_6_F_5_)_2_/B(C_6_F_5_) units [Δ*δ* ^19^F_*m*,*p*_ = 13.9 ppm, 16.0 ppm] (for details, see the ESI†).

We tried to find a mechanistic rationale for the formation of the boryl tetrahydroborole product **9** in the reaction of cyclopropylacetylene (**5b**) with two HB(C_6_F_5_)_2_ equivalents. It is known that cyclopropanes are often readily opened to the respective olefin isomers upon exposure to boron Lewis acids.^10^ Therefore, we briefly checked whether the opened isomer of **5b**, 2-methyl-1-buten-3-yne, might be involved in this reaction. However, this was not the case. Its reaction with two equivalents of HB(C_6_F_5_)_2_ took a different course (for details, see the ESI†).

**Scheme 4:**
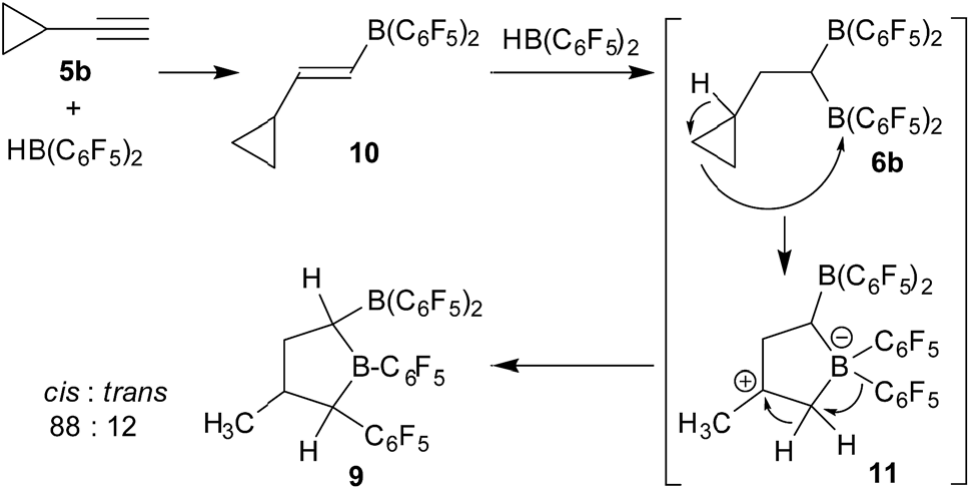
Rearrangement reaction leading to **9**.

**Scheme 5:**
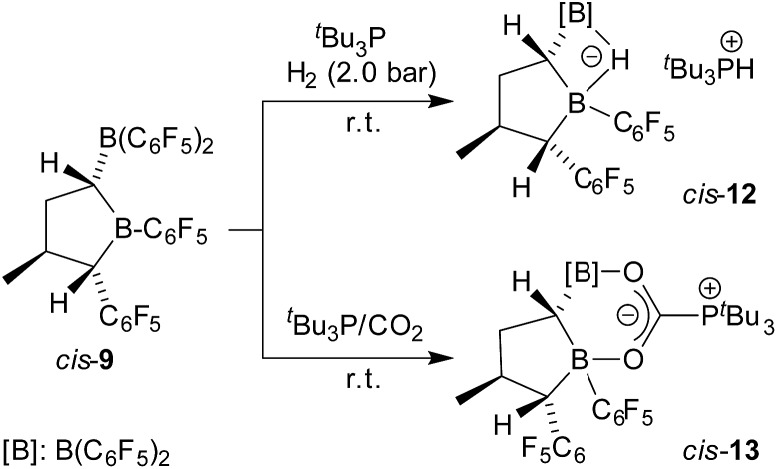
FLP reactions of compound *cis*-**9**.

Therefore, we assumed a reaction pathway as outlined in Scheme 4. It is known that **5b** undergoes a single hydroboration with Piers' borane to give **10**, so we assume it to be the initial intermediate.^11^ With a second HB(C_6_F_5_)_2_ equivalent this can then undergo the subsequent hydroboration reaction to give the geminal bis-boryl substituted compound **6b**. In the *in situ* NMR experiment we observed an intermediate which is likely **6b** (for details, see the ESI†). This is not stable under our typical reaction conditions but undergoes Lewis acid induced cyclopropyl ring opening, potentially leading to **11** which is subsequently stabilized by a sequence of hydride/C_6_F_5_ 1,2-shifts to result in the observed product **9**. We must stress that we so far have no information about the alleged intermediates on the way and we cannot convincingly explain, let alone predict, the preferred stereochemical outcome, aside from the assumption that the formation of the observed *cis*-**9** product is following a pathway of least steric hindrance on the way (Scheme 5).

**Fig. 4:**
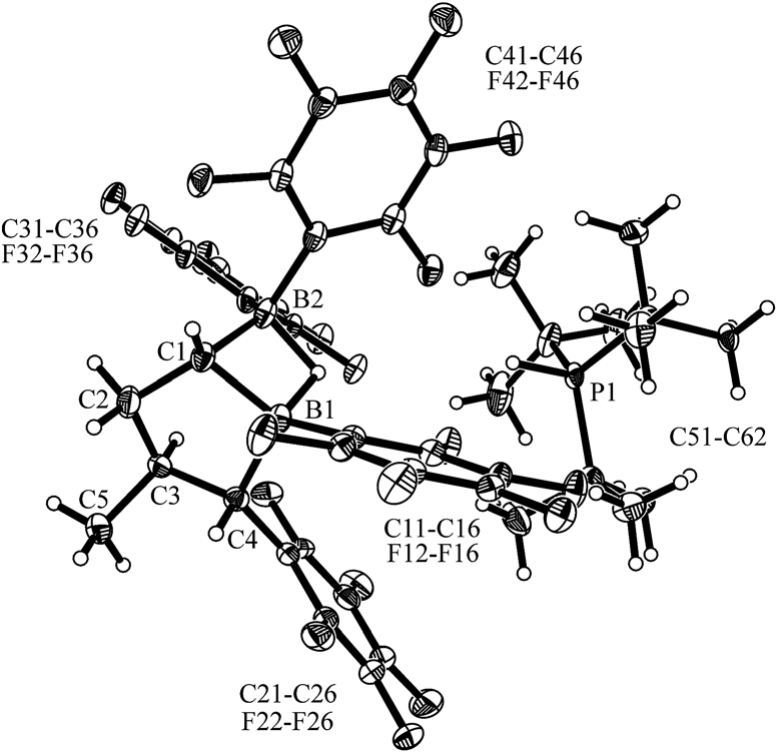
Molecular structure of the dihydrogen splitting product *cis*-**12** (thermal ellipsoids are shown with a 30% probability level). Selected bond lengths (Å) and angles (degrees): B1···B2 1.924(6), B2–C1 1.584(5), B2–H1, 1.33(3), C1–B1, 1.603(5), B1–C4 1.653(5), B1–H1 1.35(3), B2–C1–B1 74.3(2), B1–H1–B2 91.7.

The geminal bis-boryl compound contains a pair of Lewis acidic boron atoms and, consequently, it may serve as a chelate boron Lewis acid component in FLP chemistry. The isolated *cis*-**9** in conjunction with the phosphorus Lewis base P^*t*^Bu_3_ served as an active dihydrogen splitting reagent. Thus, treatment of a 1 : 1 mixture of *cis*-**9** and P^*t*^Bu_3_ with dihydrogen (2.0 bar) in pentane solution overnight produced the dihydrogen splitting product *cis*-**12** as a precipitate. The salt *cis*-**12** was isolated as a white solid with a 71% yield. We obtained single crystals of compound *cis*-**12** from pentane/dichloromethane by a diffusion method which were suitable for characterization by X-ray diffraction (see Fig. 4). In the crystal, we see the typical r-1-boryl, t-3-methyl, c-4-C_6_F_5_ arrangement^12^ of the substituents on the tetrahydroborole framework. There is now a hydride bridging between the two boron atoms.^13^ Consequently, both the boron atoms B1 and B2 have attained distorted tetrahedral coordination geometries (∑B1^ccc^ = 345.3°, ∑B2^ccc^ = 349.8°), and we found the HP^*t*^Bu_3_^+^ cation in the crystal.

The bulk isolated product *cis*-**12** (in CD_2_Cl_2_) contained *ca.* 15–20% contamination of the isomer *trans*-**12** since we had started from a not completely pure starting material (for details, see the ESI;† the characterization of the independently synthesised isomer *trans*-**12** will be described below). Compound *cis*-**12** shows a pair of ^11^B NMR signals in the typical borate chemical shift range (*δ* = –14.5 ppm, –19.7 ppm). It shows a ^31^P NMR phosphonium doublet at *δ* = 60.6 ppm with ^1^ *J*_PH_ ∼ 428 Hz. We also exposed the *cis*-**9**/P^*t*^Bu_3_ FLP (again contaminated with a small amount of *trans*-**9**) to carbon dioxide (2.0 bar, r.t., overnight) in pentane solution. Under the typical conditions, the zwitterionic FLP/CO_2_ addition product precipitated and was recovered by filtration to give *cis*-**13** as a white solid with a 73% yield. The NMR analysis (in THF-d_8_) again showed the presence of a second isomer (*trans*-**13**, see below, *ca.* 3%).

**Fig. 5:**
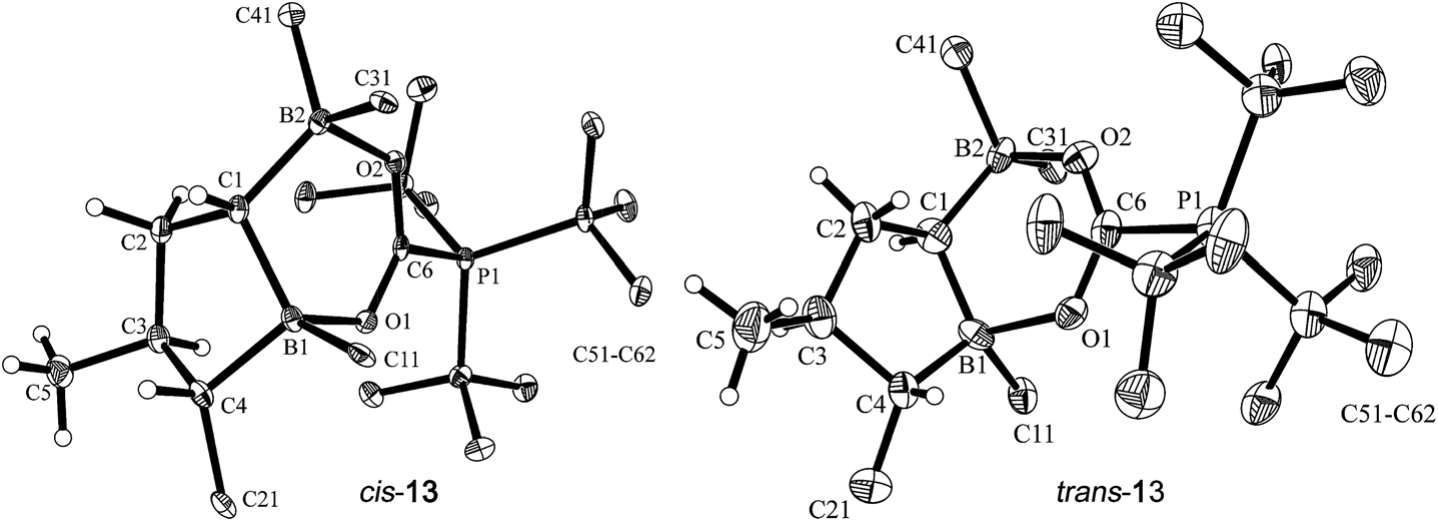
Projection of the molecular structures of the FLP/CO_2_ addition product *cis*-**13** [left, thermal ellipsoids are shown with a 50% probability level; hydrogen atoms of the P^*t*^Bu_3_ group and the C_6_F_5_ substituents at boron atoms B1, B2, and at carbon atom C4 are omitted for clarity: for details see the ESI;† selected bond lengths (Å) and angles (degrees): P1–C6 1.905(2), B1–O1 1.657(2), B2–O2 1.636(2), O2–C6 1.258(2), O1–C6 1.255(2), O1–C6–O2 128.0(2), B2–C1–B1 110.9(2)] and *trans*-**13** [right, the independent synthesis of *trans*-**13** is described below; thermal ellipsoids are shown with a 30% probability level; hydrogen atoms of the P^*t*^Bu_3_ group and the C_6_F_5_ substituents at boron atoms B1, B2, and at carbon atom C4 are omitted for clarity: for details see the ESI;† selected bond lengths (Å) and angles (degrees): P1–C6 1.913(10), B1–O1 1.717(13), B2–O2 1.634(13), O2–C6 1.248(12), O1–C6 1.271(12), O1–C6–O2 128.7(9), B2–C1–B1 118.1(9)].

Single crystals of *cis*-**13** suitable for X-ray crystal structure analysis were obtained from pentane/dichloromethane at –35 °C by the diffusion method (see Fig. 5). The compound contains a central heterocyclic six-membered ring that was formed by double chelate coordination of the geminal bis-boryl acceptor with the oxygen atoms of the phosphine activated carbon dioxide molecule. The structure of this subunit is largely delocalized with similar bond lengths in the B1–O1/B2–O2 pair as well as the C6–O1/O2 pair of carbon–oxygen bonds. Carbon atom C6 has the P^*t*^Bu_3_ group attached to it. This chelate heterocycle is interlocked with the five-membered tetrahydroborole framework, which has the boron atom B1 incorporated in it. This section of the molecule shows the same characteristic stereochemical features as we had found for its precursor *cis*-**9**. The hydrogen atoms at C1/C4 and the methyl substituent at carbon atom C3 are all in a *cis*-arrangement on this five-membered ring.

The boryl tetrahydroborole system **9** contains three independent carbon chirality centres. Therefore, there is the possibility of forming four diastereoisomers. So far our rearrangement reaction was rather stereoselective and produced the major product *cis*-**9** with the relative stereoselectivity r-1, t-3, c-4 plus a small amount of a minor isomer which probably represents one of the other three diastereoisomers, but whose relative stereochemistry we did not know. We have now prepared and characterized the isomer “*trans*-**9**” (of relative r-1, c-3, t-4 stereochemistry) by a selective isomerization process at the saturated central heterocyclic framework.

For that purpose, we treated the substituted tetrahydroborole product *cis*-**9** [r-1, t-3, c-4] with a catalytic amount (20 mol%) of the persistent nitroxide radical TEMPO (pentane, r.t., 4 days).^11,14^ This reaction apparently proceeded with reversible H-atom abstraction at the activated C1 position of the heterocycle and we isolated the *trans*-**9** epimer [r-1, c-3, t-4] as a colourless solid with a 74% yield. This compound was characterized by C,H-elemental analysis, by NMR spectroscopy (^11^B: *δ* = 79.6 ppm, 72.9 ppm, for details see the ESI†) and by X-ray diffraction. Single crystals suitable for the X-ray crystal structure analysis of compound *trans*-**9** were obtained from a pentane/dichloromethane mixture at –35 °C (see Fig. 3, right). It shows the typical five-membered tetrahydroborole framework with the B(C_6_F_5_)_2_ and C_6_F_5_ substituents at carbon atoms C1 and C4 now in a *trans* relationship. The methyl group at C3 has remained *trans* oriented to the C_6_F_5_ group at C4.

**Fig. 6:**
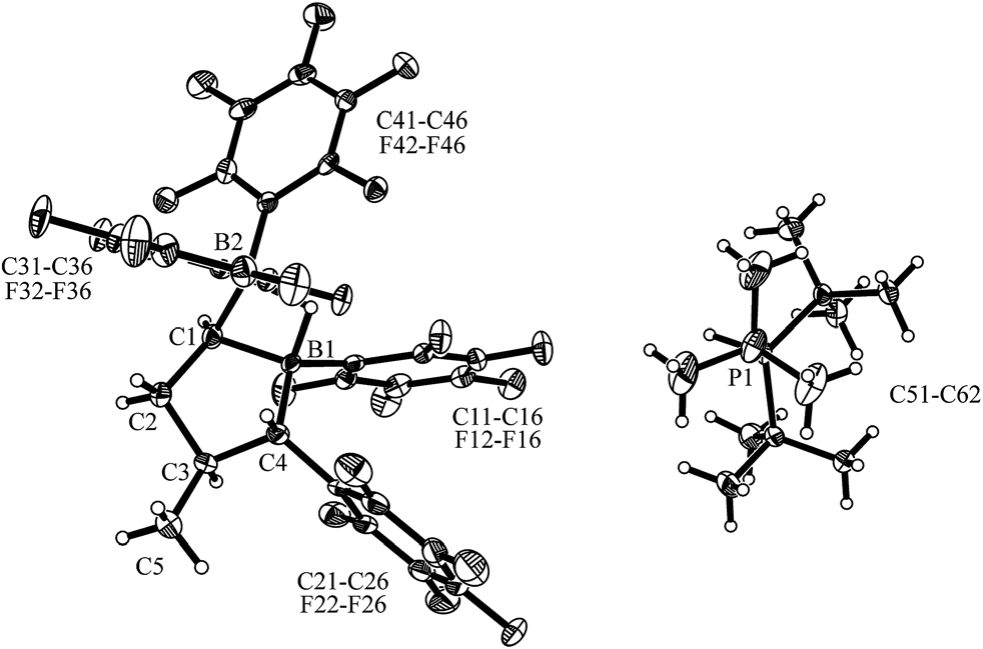
A view of the molecular structure of the hydridoborate/phosphonium salt *trans*-**12** (thermal ellipsoids are shown with a 50% probability level). Selected bond lengths (Å) and angles (degrees): B1···B2 1.909(5), B2–C1 1.584(5), B1–C1 1.596(4), B1–C4 1.636(5), B2–H1 1.31(3), B1–H1 1.30(4), B2–C1–B1 73.8(2), B1–H1–B2 93.8.

**Scheme 6:**
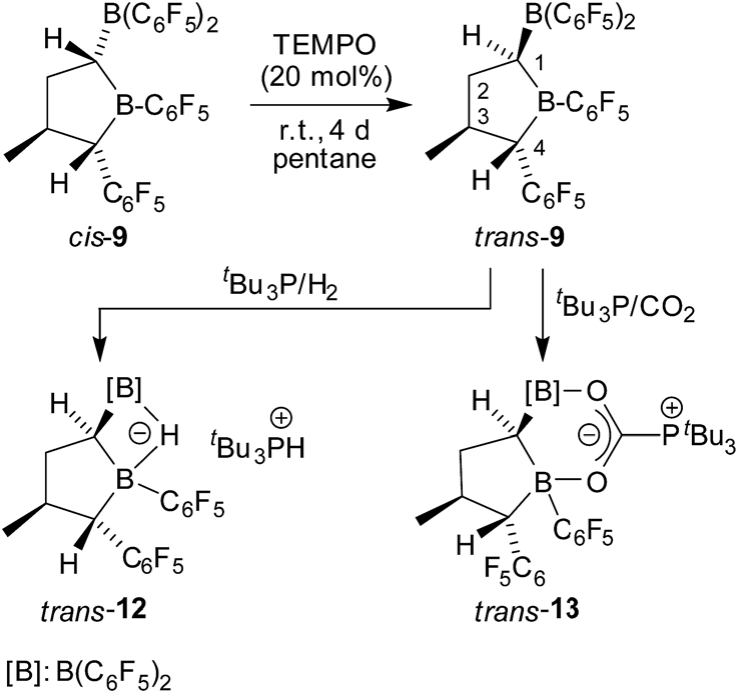
Isomerization of compound *cis*-**9** and the FLP reactions of *trans*-**9**.

Compound *trans*-**9** also formed an active frustrated Lewis pair with P^*t*^Bu_3_. The system heterolytically cleaved dihydrogen at near to ambient conditions (pentane, r.t., 2.0 bar H_2_, overnight), and we isolated the hydridoborate/phosphonium salt with a 62% yield. It shows typical ^11^B NMR signals at *δ* = –13.3 ppm and –17.1 ppm and a ^31^P NMR feature at *δ* = 60.7 ppm (^1^ *J*_PH_ ∼ 428 Hz). Compound *trans*-**12** was characterized by X-ray diffraction (single crystals were obtained from pentane/dichloromethane at r.t. by the diffusion method). The X-ray crystal structure analysis (see Fig. 6) showed the presence of the hydride bridged pair of boron atoms inside the anion and the separate HP^*t*^Bu_3_^+^ cation. The framework of compound *trans*-**12** features the expected *trans*-orientation of the B(H)(C_6_F_5_)_2_/C_6_F_5_ pair of substituents at the ring carbon atoms C1/C4 and the vicinal *trans*-orientation of the C3–CH_3_ group with the C4–C_6_F_5_ substituent. The system has consequently conserved the relative stereochemistry of the starting material *trans*-**9**. Compound *trans*-**12** shows a relative stereochemistry of r-1, c-3, t-4 (see Scheme 6 and Fig. 6).

Compound *trans*-**9** also reacts with carbon dioxide in the presence of P^*t*^Bu_3_. Exposing a mixture of *trans*-**9** and tris(*tert*-butyl)phosphine in pentane solution overnight at r.t. to a CO_2_ atmosphere gave the FLP/CO_2_ adduct *trans*-**13** as a white precipitate with a 76% yield. The compound turned out to be only sparingly soluble in many solvents. However it could be characterized by X-ray diffraction using single crystals that were directly obtained from the reaction mixture of *trans*-**9**/P^*t*^Bu_3_ with CO_2_. The structure (see Fig. 5, right) confirmed the stereochemical assignment of the backbone of the compounds of this *trans*-series: in compound *trans*-**13** the boryl substituent at carbon atom C1 is in a *trans* relationship with the C_6_F_5_ substituent at the distal ring carbon atom C4, and the latter is oriented *trans* relative to the methyl group at C3. Consequently, the relative positions of the three substituents at the central tetrahydroborole framework in compound *trans*-**13** are r-1-boryl, c-3-methyl, t-4-C_6_F_5_ configured. The CO_2_ oxygen atoms are found to be bonded to the pair of boron Lewis acid sites and the phosphorus atom is coordinated to the CO_2_ carbon atom. The CO_2_ bonding to the geminal bis(borane) acceptor is slightly unsymmetrical with the B1–O1 bond in the central position being markedly longer than the lateral B2–O2 contact and also the P1–C6 linkage is rather long (see Fig. 5). Compound *trans*-**13** was just sufficiently soluble in d_8_-THF to allow the recording of most of its NMR features. The actual sample used was *ca.* 90% pure, and it contained a minor compound of unknown composition. Compound *trans*-**13** shows a ^31^P NMR resonance at *δ* = 57.4 ppm. The ^13^C NMR signal of the CO_2_ derived moiety occurs at *δ* = 173.4 ppm (^1^ *J*_PC_ = 90.0 Hz) and compound *trans*-**13** features a total of six *o*- (two overlapping), four *p*- and four *m*-C_6_F_5_^19^F NMR signals in d_8_-THF at 233 K (for further details see the ESI†).

## Conclusions

We have shown in this study that the reaction of cyclopropylacetylene with two molar equivalents of Piers' borane [HB(C_6_F_5_)_2_] takes an unusual course. We assume that initially the usual two-fold hydroboration reaction of the terminal alkyne takes place with the anti-Markovnikov orientation generating the respective geminal bis-boryl compound. This is apparently not stable under the applied mild reaction conditions, but undergoes an intramolecular rearrangement process initiated by cyclopropyl ring opening by the adjacent strong borane Lewis acid. This initiates a series of 1,2-migration reactions involving the migration of one C_6_F_5_ group from boron to carbon which eventually yields the α-boryl tetrahydroborole system **9**. This is obtained with a rather high diastereoselectivity from this rearrangement process. The major compound *cis*-**9** is an active FLP dihydrogen cleavage reagent in the presence of the bulky P^*t*^Bu_3_ Lewis base. The *cis*-**9**/P^*t*^Bu_3_ FLP also sequesters CO_2_ cleanly in a chelate fashion, similar to the here studied more Lewis acidic geminal R-CH[B(C_6_F_5_)_2_]_2_ reference systems, despite the loss of one electron withdrawing C_6_F_5_ substituent at a boron atom. This probably indicates the favourable influence of the geminal bis-boryl situation for both chelate hydride and chelate CO_2_ binding.

## Experimental section

### Preparation of compound **6a**

A solution of 4-phenyl-1-butyne (**5a**, 65.0 mg, 0.50 mmol) in toluene (1.0 mL) was added to a suspension of bis(pentafluorophenyl)borane (345 mg, 1.00 mmol) and toluene (3.0 mL). The reaction mixture was stirred at room temperature for 1 hour and then the suspension was filtered by cannula filtration. The volatiles of the obtained filtrate were removed *in vacuo* to give a colorless oil. Subsequently pentane (4.0 mL) was added and the mixture was stored at *ca.* –35 °C overnight. The formed white powder was isolated by filtration, washed with pentane (2 × 1 mL) and dried *in vacuo* to give compound **6a** (312 mg, 0.38 mmol, 76%) as a white solid. Anal. calc. for C_34_H_12_B_2_F_20_: C, 49.68%; H, 1.47%. Found: C, 49.40%; H, 1.40%. For the NMR data see the ESI.†

### Preparation of compound **7**

A solution of compound **6a** (82.2 mg, 0.10 mmol) and tri-*tert*-butylphosphine (20.5 mg, 0.10 mmol) in pentane (3.0 mL) was exposed to a hydrogen atmosphere (2.0 bar) at room temperature and stirred overnight. The resulting white precipitate was collected by cannula filtration and washed with pentane (3 × 2 mL). After the removal of all volatiles *in vacuo*, compound **7** was obtained (88.6 mg, 0.087 mmol, 87%) as a white solid. Anal. calc. for C_46_H_41_B_2_F_20_P: C, 53.83%; H, 4.03%. Found: C, 53.81%; H, 4.01%. Single crystals suitable for the X-ray crystal structure analysis were obtained by the slow diffusion of pentane into a solution of compound **7** in dichloromethane at –35 °C.

### Preparation of compound **8**

A solution of compound **6a** (123.3 mg, 0.15 mmol) and tri-*tert*-butylphosphine (30.3 mg, 0.15 mmol) in pentane (5.0 mL) was exposed to CO_2_ (2.0 bar) at room temperature and then stirred for 2 hours. The resulting white precipitate was isolated by cannula filtration and washed with pentane (3 × 1 mL). After drying the solid *in vacuo*, compound **8** (129.4 mg, 0.12 mmol, 81%) was obtained as a white powder. Anal. calc. for C_47_H_39_B_2_F_20_O_2_P: C, 52.84%; H, 3.68%. Found: C, 53.21%; H, 3.91%. Single crystals of compound **8** suitable for the X-ray crystal structure analysis were obtained by the slow diffusion of pentane into a solution of the white powder in dichloromethane at –35 °C.

### Preparation of compound *cis*-**9**

A solution of compound **5b** (33.0 mg, 0.50 mmol) in toluene (1.0 mL) was added to a suspension of bis(pentafluorophenyl)borane (345 mg, 1.00 mmol) and toluene (3.0 mL). After stirring the reaction mixture at room temperature for 1 hour, the solution was separated from the resulting suspension by cannula filtration. Then all volatiles of the filtrate were removed *in vacuo* to give a yellow oil, which was dissolved in pentane (2.5 mL) and stored at –35 °C overnight. The precipitated pale yellow solid was isolated by filtration and washed with cold pentane (2 × 0.5 mL). The removal of all volatiles *in vacuo* gave a pale yellow solid (253 mg, 0.34 mmol, 67%). Anal. calc. for C_29_H_8_B_2_F_20_: C, 45.95 %; H, 1.06%. Found: C, 45.74%; H, 1.07%. Crystals of compound *cis*-**9** suitable for the X-ray crystal structure analysis were obtained from a solution of the yellow solid in pentane at –35 °C.

### Preparation of compound *trans*-**9**

TEMPO (16.6 mg, 0.11 mmol) was added to a solution of compound *cis*-**9** (400 mg, 0.53 mmol) in pentane (15 mL). After stirring the reaction mixture at r.t. for 4 days, the resulting suspension was concentrated to about 2.0 mL, and stored in the fridge (–35 °C) overnight. The precipitated white powder was isolated *via* cannula filtration, and washed with cold pentane (2 × 1.0 mL). The removal of all volatiles under reduced pressure gave product *trans*-**9** (296 mg, 0.39 mmol, 74%) as a white solid. Anal. calc. for C_29_H_8_B_2_F_20_: C, 45.95%; H, 1.06%. Found: C, 45.45%; H, 0.95%. Crystals suitable for the X-ray crystal structure analysis were obtained from a solution of compound *trans*-**9** in pentane (1.5 mL) and CH_2_Cl_2_ (0.5 mL) at –35 °C.

### Preparation of compound *cis*-**12**

A solution of compound *cis*-**9** (*cis*/*trans* **≈** 96/4, *vide supra*) (113.7 mg, 0.15 mmol) and tri-*tert*-butylphosphine (30.3 mg, 0.15 mmol) in pentane (5.0 mL) was exposed to dihydrogen (2.0 bar) at room temperature and then stirred overnight. The formed white precipitate was collected by cannula filtration and washed with pentane (3 × 1 mL). After the removal of all volatiles *in vacuo*, a white solid was obtained (101.4 mg, 0.11 mmol, 71%). Anal. calc. for C_41_H_37_B_2_F_20_P: C, 51.17%; H, 3.88%. Found: C, 50.96%; H, 3.76%. Single crystals of compound *cis*-**12** suitable for the X-ray crystal structure analysis were obtained by the slow diffusion of *n*-pentane into a solution of the white solid in dichloromethane at room temperature.

### Preparation of compound *trans*-**12**

A solution of compound *trans*-**9** (75.8 mg, 0.10 mmol) and tri-*tert*-butylphosphine (20.2 mg, 0.10 mmol) in pentane (4.0 mL) was exposed to a dihydrogen atmosphere (2.0 bar) at room temperature and stirred overnight. The formed white precipitate was collected by cannula filtration and washed with *n*-pentane (2 × 1 mL). The removal of all volatiles *in vacuo* gave compound *trans*-**12** (59.2 mg, 0.062 mmol, 62%) as a white solid. Anal. calc. for C_41_H_37_B_2_F_20_P: C, 51.17%; H, 3.88%. Found: C, 51.09%; H, 3.67%. Single crystals suitable for X-ray crystal structure analysis were obtained by the slow diffusion of pentane into a solution of compound *trans*-**12** in dichloromethane at room temperature.

### Preparation of compound *cis*-**13**

A solution of compound *cis*-**9** (*cis*/*trans* **≈** 96/4, *vide supra*) (113.7 mg, 0.15 mmol) and tri-*tert*-butylphosphine (30.3 mg, 0.15 mmol) in pentane (5.0 mL) was exposed to a CO_2_ atmosphere (2.0 bar) and then stirred overnight at room temperature. The formed white precipitate was collected by cannula filtration and washed with pentane (3 × 1 mL). After the removal of all volatiles *in vacuo*, a white solid was obtained (108.3 mg, 0.11 mmol, 73%). Anal. calc. for C_42_H_35_B_2_F_20_O_2_P: C, 50.23%; H, 3.51%. Found: C, 50.07%; H, 3.36%. Single crystals of compound *cis*-**13** suitable for the X-ray crystal structure analysis were obtained by the slow diffusion of *n*-pentane into a solution of the obtained white solid in dichloromethane at –35 °C.

### Preparation of compound *trans*-**13**

A solution of compound *trans*-**9** (75.8 mg, 0.10 mmol) and tri-*tert*-butylphosphine (20.2 mg, 0.10 mmol) in pentane (5.0 mL) was exposed to CO_2_ (2.0 bar) at room temperature and then stirred overnight. The formed white precipitate was collected by cannula filtration and washed with pentane (3 × 1 mL). After the removal of all volatiles *in vacuo*, compound *trans*-**13** (76.2 mg, 0.076 mmol, 76%) was obtained as a white solid. Anal. calc. for C_42_H_35_B_2_F_20_O_2_P: C, 50.23%; H, 3.51%. Found: C, 49.96%; H, 3.27%. Single crystals of compound *trans*-**13** suitable for the X-ray crystal structure analysis were obtained directly from a reaction solution of compound *trans*-**9** (37.9 mg) and tri-*tert*-butylphosphine (10.1 mg) and dichloromethane (1.0 mL) in a CO_2_ atmosphere (2.0 bar) at room temperature.
