# Supplementary material for: Geminal bis-borane formation by borane Lewis acid induced cyclopropyl rearrangement and its frustrated Lewis pair reaction with carbon dioxide
Source: Chem Sci. 2016 Sep 16;8(2):1097–104. doi: 10.1039/c6sc03468c (PMC5369401; doi:10.1039/c6sc03468c)
Supplement: Supplementary file 1 [file SC-008-C6SC03468C-s001.pdf]

Supporting Information

Geminal Bis-borane Formation by Borane Lewis Acid Induced Cyclopropyl Rearrangement and its Frustrated Lewis Pair Reaction with Carbon Dioxide

Yun-Lin Liu, Gerald Kehr, Constantin G. Daniliuc and Gerhard Erker

Organisch-Chemisches Institut, Universität Münster, Corrensstrasse 40, 48149 Münster, Germany.

Experiment Section

General Information. All reactions involving air- or moisture-sensitive compounds were carried out under an inert gas atmosphere (Argon) by using Schlenk-type glassware or in a glovebox. All solvents were dried and degassed before use, if necessary for the respective reaction. Chemicals: Unless otherwise noted all chemicals were used as purchased. The following instruments were used for physical characterization of the compounds: melting points: TA-instruments DSC Q-20; elemental analyses: Foss-Heraeus CHNO-Rapid; IR: Varian 1300 FT-IR; NMR spectra were recorded on the following instruments: Agilent DD2 500 (^{1}H: 500 MHz, ^{13}C: 126 MHz, ^{19}F: 470 MHz, ^{11}B:160 MHz, ^{31}P:202 MHz), Agilent DD2 600 (^{1}H: 600 MHz, ^{13}C: 151 MHz, ^{19}F: 564 MHz, ^{11}B: 192MHz, ^{31}P:243 MHz). ^{1}H NMR and ^{13}C NMR: chemical shift is given relative to tetramethylsilane and referenced to the solvent signal. ^{19}F NMR: chemical shift is given relative to $CFCl_3$ (external reference, δ ^{19}F($CFCl_3$) = 0); ^{11}B NMR: chemical shift is given relative to $BF_3{\cdot}Et_2O$ (external reference, δ^{11}B($BF_3{\cdot}OEt_2$) = 0); ^{31}P NMR: chemical shift is given relative to H_3PO_4 (85% in D_2O) (external reference, δ^{31}P(H_3PO_4) = 0).

X-Ray diffraction: For compounds **7** and ***trans*-13** data sets were collected with a Nonius Kappa CCD diffractometer. Programs used: data collection, COLLECT (R. W. W. Hooft, Bruker AXS, 2008, Delft, The Netherlands); data reduction Denzo-SMN (Z. Otwinowski, W. Minor, *Methods Enzymol.* **1997**, *276*, 307-326); absorption correction, Denzo (Z. Otwinowski, D. Borek, W. Majewski, W. Minor, *Acta Crystallogr*. **2003**, *A59*, 228-234); structure solution SHELXS-97 (G. M. Sheldrick, *Acta Crystallogr*. **1990**, *A46*, 467-473); structure refinement SHELXL-97 (G. M. Sheldrick, *Acta Crystallogr*. **2008**, *A64*, 112-122) and graphics, XP (BrukerAXS, 2000). For compounds **8** and ***cis*-9** data sets were collected with a Kappa CCD APEXII Bruker diffractometer. For compounds ***trans*-9**, ***cis*-12**, ***trans*-12** and ***cis*-13** data sets were collected with a D8 Venture Dual Source 100 CMOS diffractometer. Programs used: data collection: APEX2 V2014.5-0 (Bruker AXS Inc., 2014); cell refinement: SAINT V8.34A (Bruker AXS Inc., 2013); data reduction: SAINT V8.34A (Bruker AXS Inc., 2013); absorption correction, SADABS V2014/2 (Bruker AXS Inc., 2014); structure solution SHELXT-2014 (Sheldrick, 2014); structure refinement SHELXL-2014 (Sheldrick, 2014) and graphics, XP (Bruker AXS Inc., 2014). *R*-values are given for observed reflections, and wR^2 values are given for all reflections. *Exceptions and special features*: For compounds **7**, **8** and **cis-13** one dichloromethane molecule, for compound ***trans*-12** the tBu$_3$PH cation and for compound ***cis*-12** one dichloromethane molecule, the tBu$_3$PH cation and the five membered ring (C1-C2-C3-C4-B1 ring) containing the C_6F_5 substituent were found disordered over two positions in the asymmetric unit. For all these compounds several restraints (SADI, SAME, ISOR and

SIMU) were used in order to improve the refinement stability. One badly disordered pentane molecule for compound **8**, a second dichloromethane molecule for compound ***cis*-12**, and one and a half dichloromethane molecule in compound ***trans*-12** were found in the asymmetrical unit and could not be satisfactorily refined. The program SQUEEZE (A. L. Spek J. Appl. Cryst., 2003, 36, 7-13) was therefore used to remove mathematically the effect of the solvents. The quoted formula and derived parameters are not included the squeezed solvent molecules. Compound ***trans*-13** was refined using the 'HKLF 5' option, whereby the BASF factor was refined to 0.22.

Materials. Bis(pentafluorophenyl)borane (*Piers'* borane) was prepared according to a procedure described in the literature. [D. J. Parks, R. E. von H. Spence and W. E. Piers, *Angew. Chem., Int. Ed.,* 1995, **34**, 809; D. J. Parks, W. E. Piers and G. P. A. Yap, *Organometallics*, 1998, **17**, 5492.]

Preparation of compound **6a**:

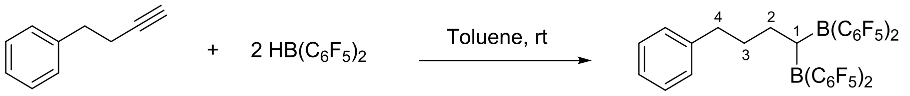

5a **6a**

A solution of 4-phenyl-1-butyne (**5a**, 65.0 mg, 0.50 mmol) in toluene (1.0 mL) was added to a suspension of bis(pentafluorophenyl)borane (345 mg, 1.00 mmol) and toluene (3.0 mL). The reaction mixture was stirred at room temperature for 1 hour and then the suspension was filtered by cannula filtration. The volatiles of the obtained filtrate were removed in vacuo to give a colorless oil. Subsequently pentane (4.0 mL) was added to the oil and the mixture was stored at ca. -35 °C overnight. The formed white powder was isolated by filtration, washed with pentane (2 × 1 mL) and dried in vacuo to give compound **6a** (312 mg, 0.38 mmol, 76%) as a white solid.

Melting point: 100 °C.

Anal. Calc. for $C_{34}H_{12}B_2F_{20}$: C, 49.68; H, 1.47. Found: C, 49.40; H, 1.40.

^{1}H NMR (600 MHz, 299 K, C_6D_6): δ 7.01 (m, 2H, *m*-Ph), 6.96 (m, 1H, *p*-Ph), 6.78 (m, 2H, *o*-Ph), 3.48 (t, $^3J_{HH}$ = 5.5 Hz, 1H, 1-CH), 2.26 (m, 2H, 4-CH_2), 1.97 (m, 2H, 2-CH_2), 1.48 (m, 2H, 3-CH_2).

^{13}C{^{1}H} NMR (151 MHz, 299 K, C_6D_6): δ 145.9 (dm, $^1J_{FC}$ ~ 246 Hz, C_6F_5), 143.4 (dm, $^1J_{FC}$ ~ 260 Hz, C_6F_5), 140.9 (*i*-Ph), 137.5 (dm, $^1J_{FC}$ ~ 254 Hz, C_6F_5), 128.48 (*m*-Ph), 128.45 (*o*-Ph), 126.2 (*p*-Ph), 113.5 (br m, *i*-C_6F_5), 59.6 (br, 1-CH), 35.9 (m, 4-CH_2), 35.4 (3-CH_2), 29.4 (2-CH_2).

^{1}H, ^{13}C GHSQC (600 MHz / 151 MHz, 299 K, C_6D_6): δ ^{1}H / δ ^{13}C: 7.01 / 128.48 (*m*-Ph), 6.96 / 126.2 (*p*-Ph), 6.78 / 128.45 (*o*-Ph), 3.48 / 59.6 (1-CH), 2.26 / 35.9 (4-CH_2), 1.97 / 29.4 (2-CH_2), 1.48 / 35.4 (3-CH_2).

^{19}F NMR (564 MHz, 299 K, C_6D_6): δ -130.7 (m, 2F, *o*-C_6F_5), -146.3 (t, $^3J_{FF}$ = 20.8 Hz, 1F, *p*-C_6F_5), -160.0 (m, 2F, *m*-C_6F_5) [$\Delta\delta^{19}F_{m,p}$ = 13.7].

^{11}B{^{1}H} NMR (192 MHz, 299 K, C_6D_6): δ 72.1 ($\nu_{1/2}$ ~ 1500 Hz).

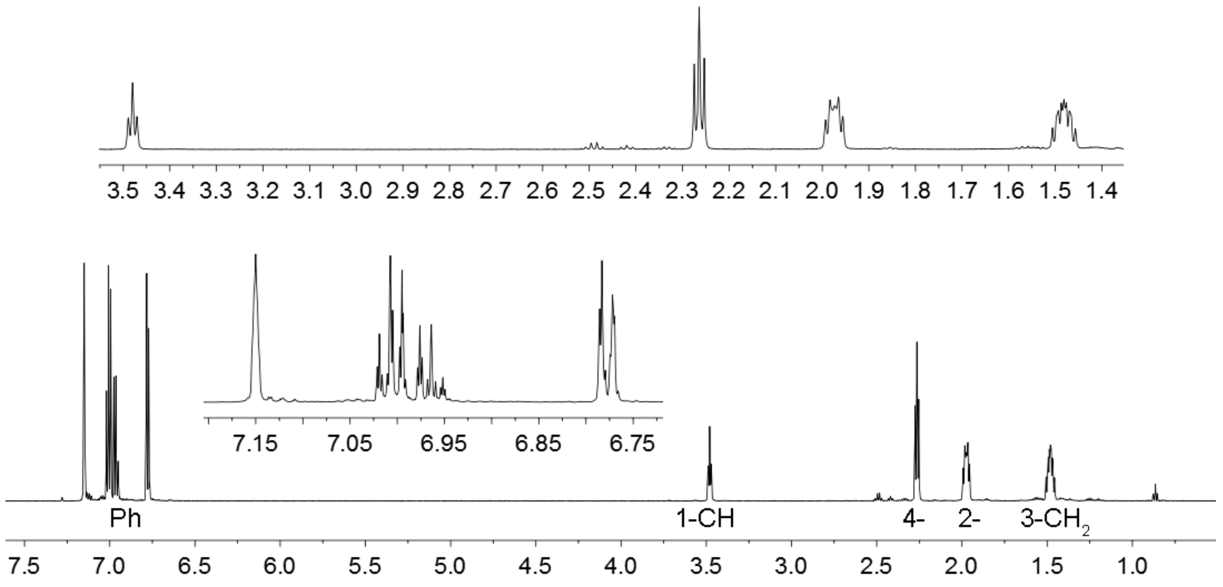

^{1}H NMR (600 MHz, 299 K, C_6D_6) spectrum of compound ***6a***.

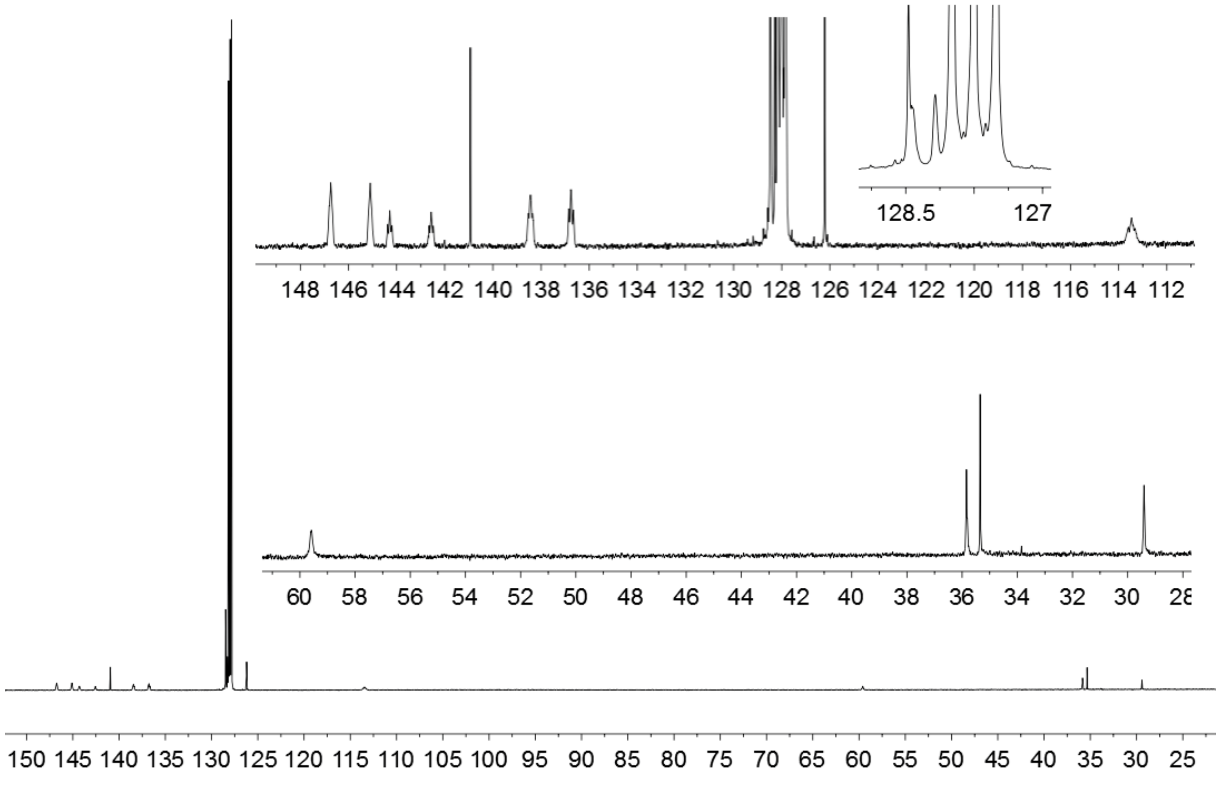

$^{13}C\{^{1}H\}$ NMR (151 MHz, 299 K, C_6D_6) spectrum of compound ***6a***.

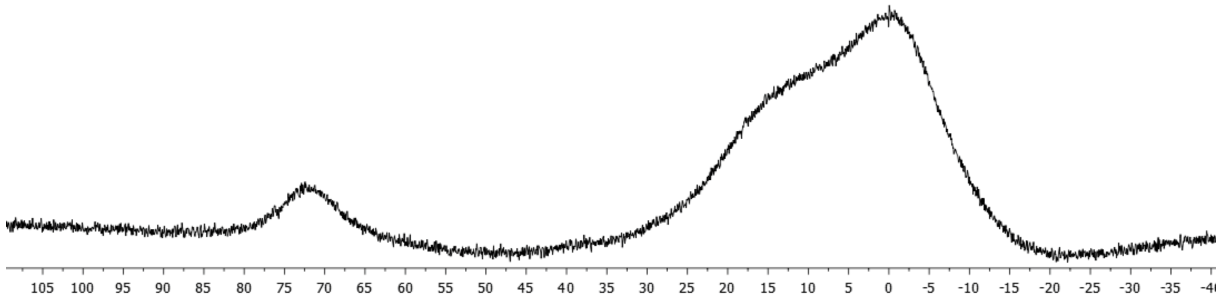

$^{11}B\{^{1}H\}$ NMR (192 MHz, 299 K, C_6D_6) spectrum of compound ***6a***.

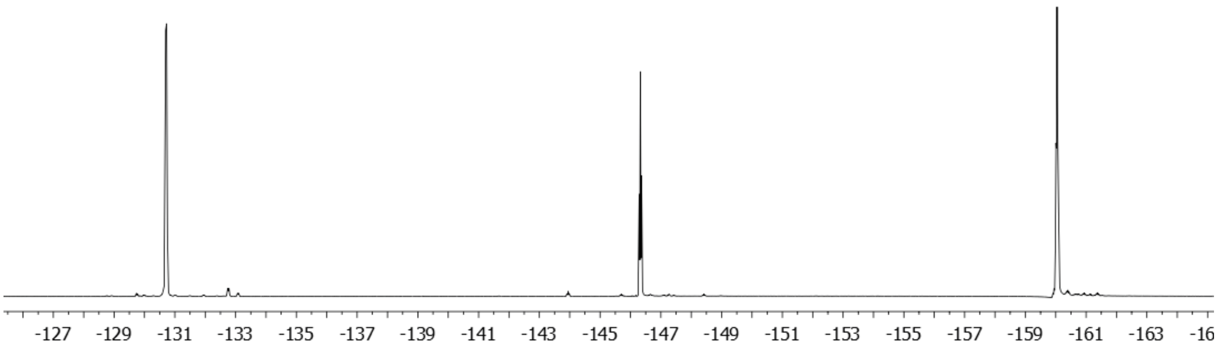

*^{19}F NMR (564 MHz, 299 K, C_6D_6) spectrum of compound **6a**.*

Preparation of compound **7**:

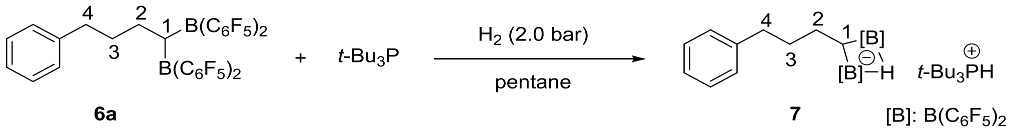

A solution of compound **6a** (82.2 mg, 0.10 mmol) and *tri*-*tert*-butylphosphane (20.5 mg, 0.10 mmol) in pentane (3.0 mL) was exposed to a hydrogen atmosphere (2.0 bar) at room temperature and stirred overnight. The resulting white precipitate was collected by cannula filtration and washed with pentane (3 x 2 mL). After removal of all volatiles in vacuo, compound **7** was obtained (88.6 mg, 0.087 mmol, 87%) as a white solid.

Melting point: 136 °C.

Anal. Calc. for $C_{46}H_{41}B_2F_{20}P$: C, 53.83; H, 4.03. Found: C, 53.81; H, 4.01.

^{1}H NMR (600 MHz, 299 K, CD_2Cl_2): δ 7.18 (m, 2H, *m*-Ph), 7.08 (m, 1H, *p*-Ph), 7.04 (m, 2H, *o*-Ph), 5.10 (d, $^1J_{PH}$ = 429.2 Hz, 1H, PH), 2.64 (br, 1H, BH), 2.48 (m, 2H, 4-CH_2), 1.65 (d, $^3J_{PH}$ = 12.0 Hz, 27H, t-Bu), 1.59 (m, 2H, 3-CH_2), 1.36 (m, 1H, 1-CH), 1.23 (m, 2H, 2-CH_2).

^{13}C{^{1}H} NMR (151 MHz, 299 K, CD_2Cl_2): δ 144.5 (*i*-Ph), 128.7 (*o*-Ph), 128.2 (*m*-Ph), 125.3 (*p*-Ph), 38.1 (d, $^1J_{PC}$ = 26.8 Hz, t-Bu), 36.9 (4-CH_2), 35.2 (3-CH_2), 30.6 (2-CH_2), 30.4 (t-Bu), 11.5 (br, 1-CH), [C_6F_5 not listed].

^{1}H, ^{13}C GHSQC (600 MHz / 151 MHz, 299 K, CD_2Cl_2): δ^{1}H/δ^{13}C 7.18 / 128.2 (*m*-Ph), 7.08 / 125.3 (*p*-Ph), 7.04 / 128.7 (*o*-Ph), 2.48 / 36.9 (4-CH_2), 1.65 / 30.4 (t-Bu), 1.59 / 35.2 (3-CH_2), 1.36 / 11.5 (1-CH), 1.23 / 30.6 (2-CH_2).

^{19}F NMR (564 MHz, 299 K, CD_2Cl_2): δ -129.5, -131.4 (each br, each 2F, *o*-C_6F_5), -161.2 (t, $^3J_{FF}$ = 20.2 Hz, 1F, *p*-C_6F_5), -161.6 (t, $^3J_{FF}$ = 20.4 Hz, 1F, *p*-C_6F_5), -166.2, -166.5 (each br m, each 2F, *m*-C_6F_5).

^{11}B{^{1}H} NMR (192 MHz, 299 K, CD_2Cl_2): δ -18.8 ($\nu_{1/2}$ ~ 350 Hz).

^{31}P{^{1}H} NMR (243 MHz, 299 K, CD_2Cl_2): δ 60.2 ($\nu_{1/2}$ ~ 20 Hz).

^{31}P NMR (243 MHz, 299 K, CD_2Cl_2): δ 60.2 (dm, $^1J_{PH}$ ~ 430 Hz).

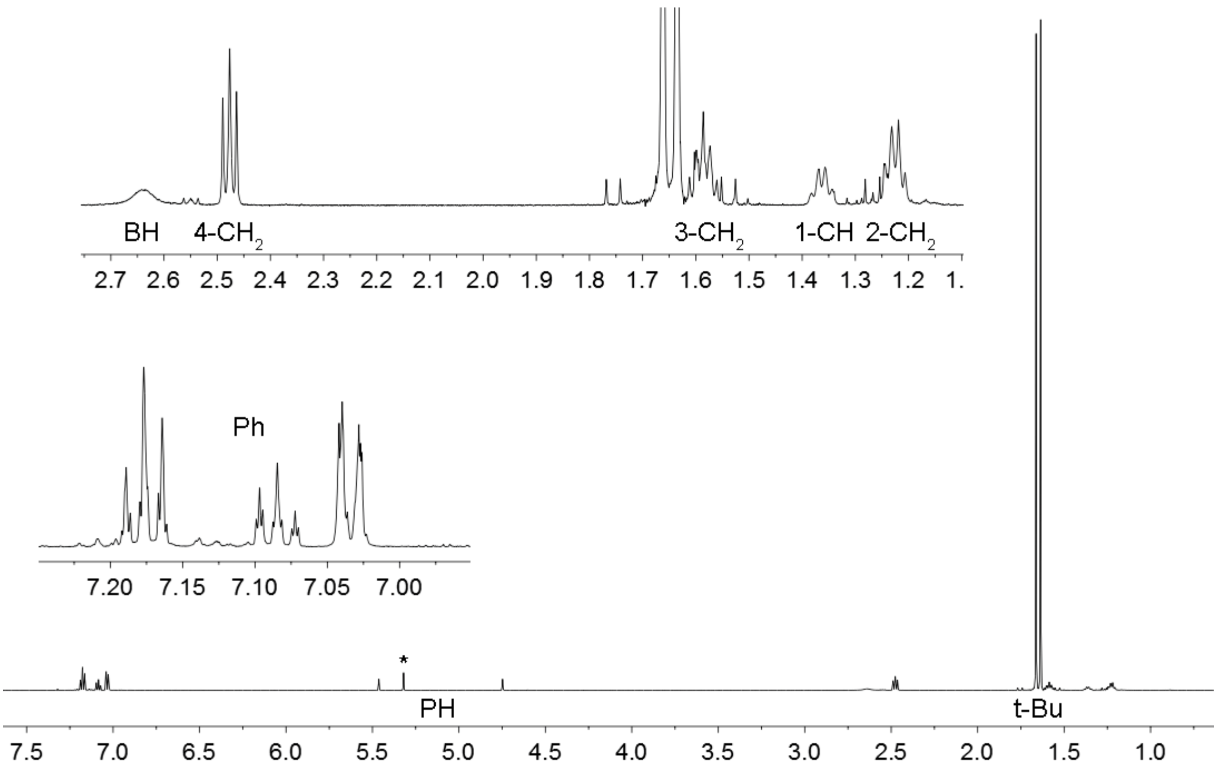

^{1}H NMR (600 MHz, 299 K, CD_2Cl_2*) spectrum of compound **7**.

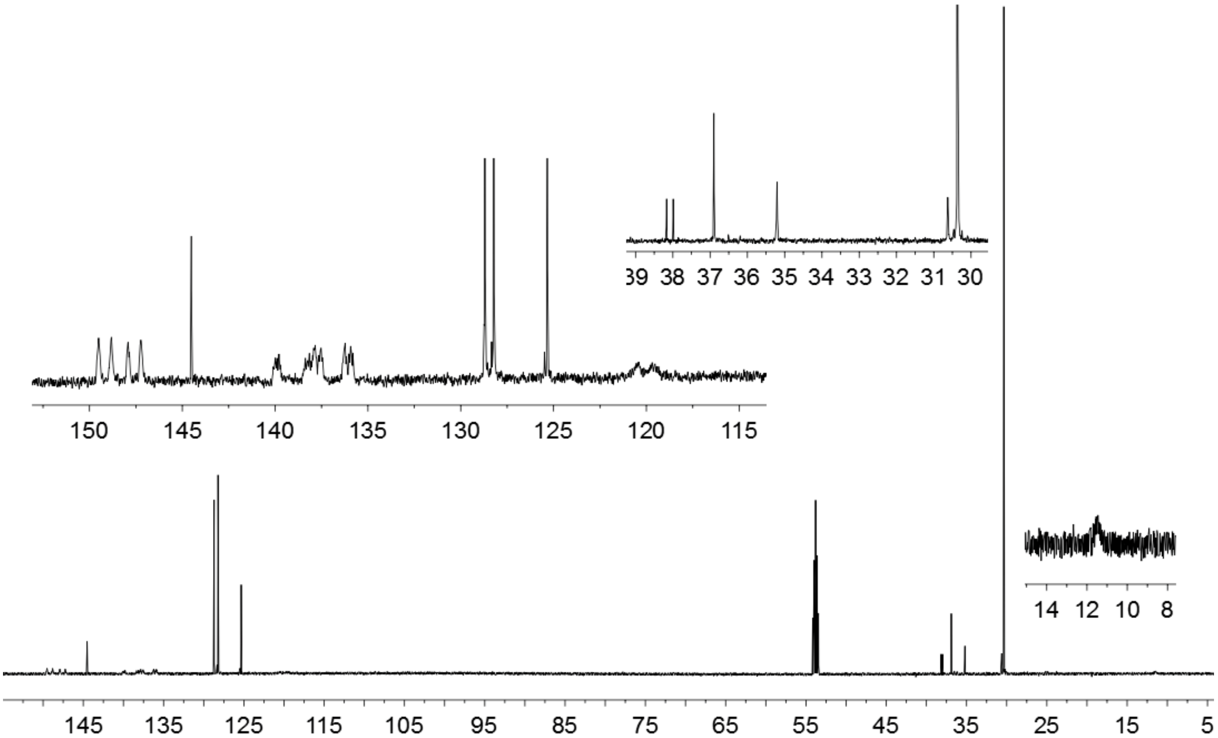

$^{13}C\{^{1}H\}$ NMR (151 MHz, 299 K, CD_2Cl_2) spectrum of compound **7**.

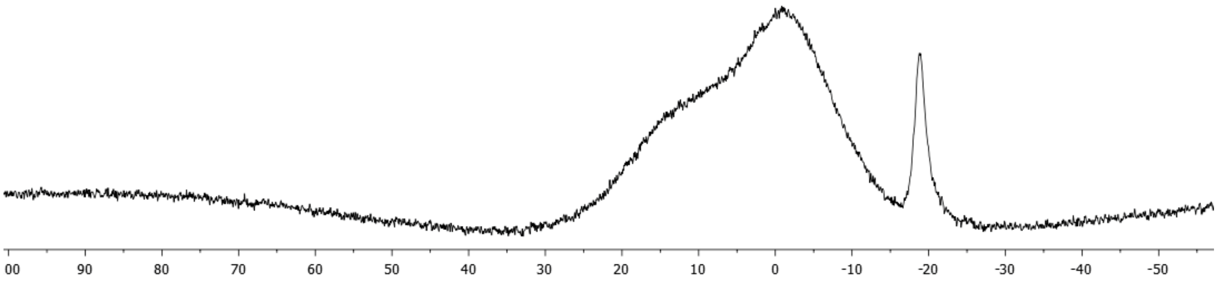

*^{11}B {^{1}H} NMR (192 MHz, 299 K, CD_2Cl_2) spectrum of compound **7**.*

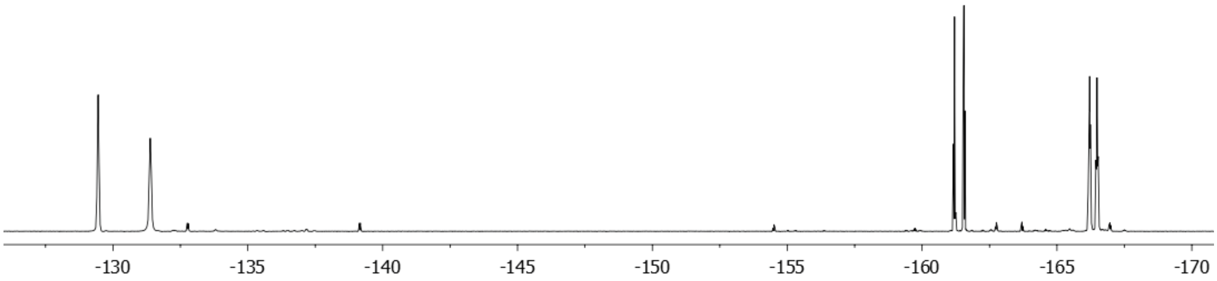

*^{19}F NMR (564 MHz, 299 K, CD_2Cl_2) spectrum of compound **7**.*

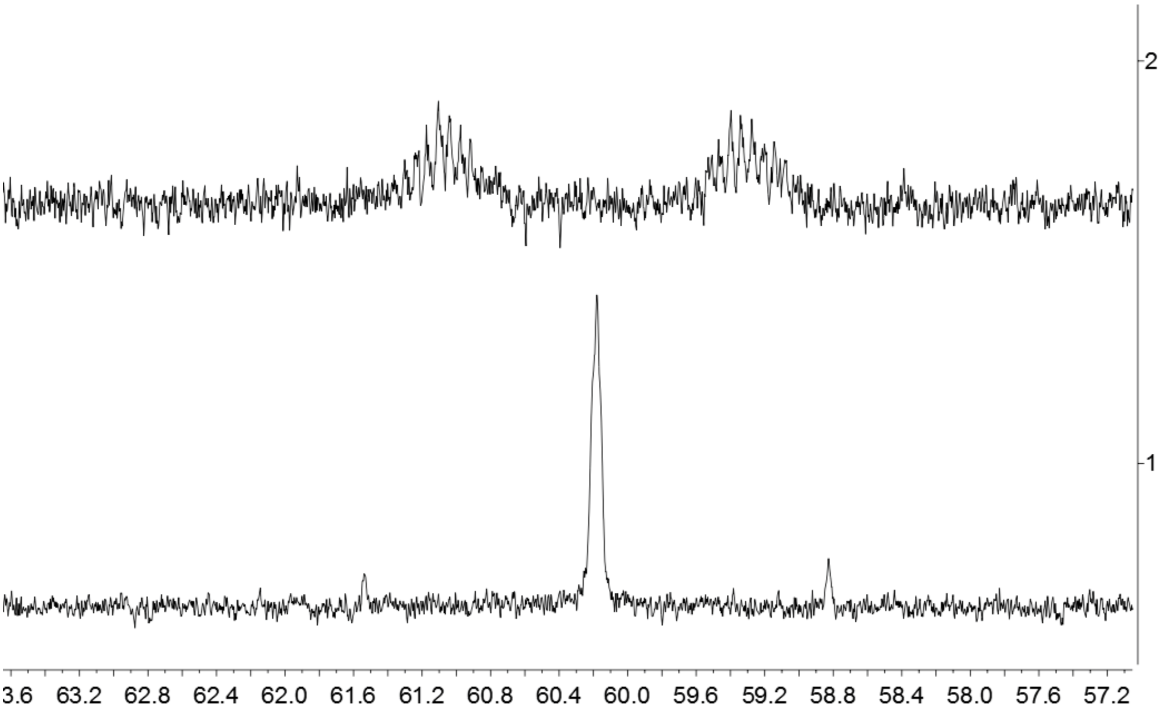

*(1) ^{31}P{^{1}H} and (2) ^{31}P NMR (243 MHz, 299 K, CD_2Cl_2) spectrum of compound **7**.*

Single crystals suitable for the X-ray crystal structure analysis were obtained by slow diffusion of pentane into a solution of compound **7** in dichloromethane at −35 °C.

X-ray crystal structure analysis of compound 7: formula $C_{46}H_{41}B_2F_{20}P \cdot CH_2Cl_2$, *M* = 1111.30, colourless crystal, 0.16 x 0.12 x 0.08 mm, *a* = 10.1634(2), *b* = 14.7985(3), *c* = 17.1628(4) Å, α = 96.740(1), *β* = 97.620(1), γ = 105.399(1)°, *V* = 2435.1(1) $Å^3$, ρ_{calc} = 1.516 gcm^{-3}, μ = 0.277 mm^{-1}, empirical absorption correction (0.957 ≤ T ≤ 0.978), *Z* = 2, triclinic, space group $P\bar{1}$ (No. 2), λ = 0.71073 Å, *T* = 223(2) K, ω and ϕ scans, 23728 reflections collected (±*h*, ±*k*, ±*l*), 8454 independent (R_{int} = 0.047) and 6396 observed reflections [$I>2\sigma(I)$], 694 refined parameters, *R* = 0.065, wR^2 = 0.140, max. (min.) residual electron density 0.28 (-0.33) e.$Å^{-3}$, the hydrogens at P1 and B1 atoms were refined freely; others were calculated and refined as riding atoms.

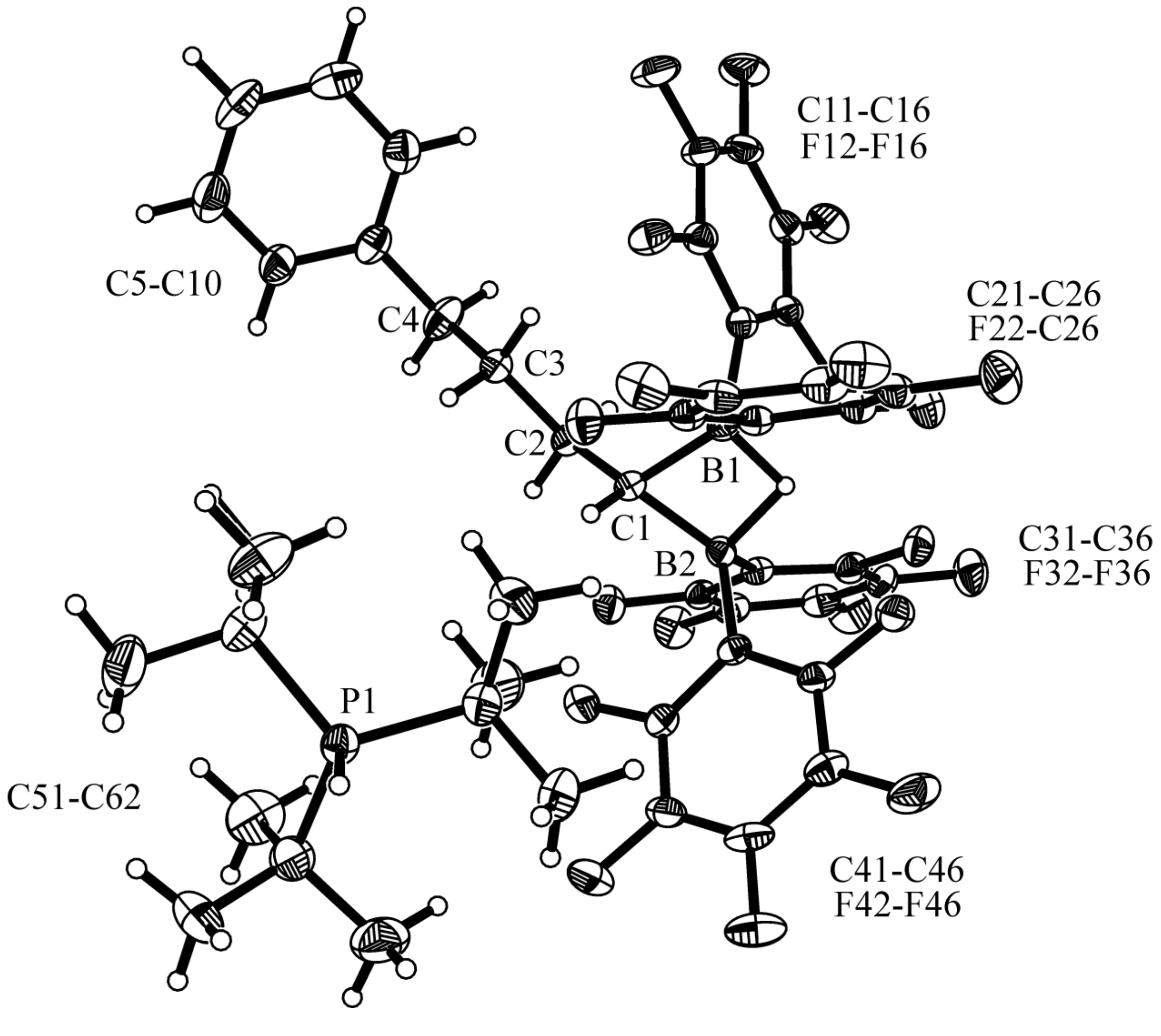

Preparation of compound **8**:

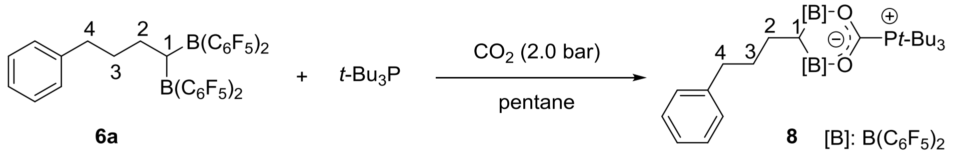

1st Experiment: A solution of compound **6a** (123.3 mg, 0.15 mmol) and *tri-tert*-butylphosphane (30.3 mg, 0.15 mmol) in pentane (5.0 mL) was exposed to CO_2 (2.0 bar) at room temperature and then stirred for 2 hours. The resulting white precipitate was isolated by cannula filtration and washed with pentane (3 x 1 mL). After drying the solid in vacuo, compound **8** (129.4 mg, 0.12 mmol, 81%) was obtained as a white powder.

Melting point: 103 °C.
IR (KBr, selective wavenumbers): ṽ [cm^{-}1] = 1587 (s, C-O asym. stretch).
Anal. Calc. for $C_{47}H_{39}B_2F_{20}O_2P$: C, 52.84; H, 3.68. Found: C, 53.21; H, 3.91.

Single crystals of compound **8** suitable for the X-ray crystal structure analysis were obtained by slow diffusion of pentane into a solution of the white powder in dichloromethane at −35 °C.

X-ray crystal structure analysis of compound 8: A colorless prism-like specimen of $C_{47}H_{39}B_2F_{20}O_2P \cdot CH_2Cl_2$, approximate dimensions 0.060 mm x 0.120 mm x 0.180 mm, was used for the X-ray crystallographic analysis. The X-ray intensity data were measured. A total of 2291 frames were collected. The total exposure time was 28.14 hours. The frames were integrated with the Bruker SAINT software package using a wide-frame algorithm. The integration of the data using a monoclinic unit cell yielded a total of 9427 reflections to a maximum θ angle of 66.59° (0.84 Å resolution), of which 9427 were independent (average redundancy 1.000, completeness = 99.8%, R_{int} = 11.29%, R_{sig} = 9.43%) and 7055 (74.84%) were greater than 2σ(F^2). The final cell constants of a = 13.061(2) Å, b = 22.030(4) Å, c = 18.545(4) Å, β = 91.787(7)°, volume = 5333.4(17) Å^3, are based upon the refinement of the XYZ-centroids of 9949 reflections above 20 σ(I) with 6.231° < 2θ < 132.4°. Data were corrected for absorption effects using the multi-scan method (SADABS). The ratio of minimum to maximum apparent transmission was 0.671. The calculated minimum and maximum transmission coefficients (based on crystal size) are 0.6770 and 0.8720. The final anisotropic full-matrix least-squares refinement on F^2 with 713 variables converged at R1 = 6.04%, for the observed data and wR2 = 15.85% for all data. The goodness-of-fit was 1.065. The largest peak in the final difference electron density synthesis was 0.386 e$^-$/Å^3 and the largest hole was -0.540 e$^-$/Å^3 with an RMS deviation of 0.076 e$^-$/Å^3. On the basis of the final model, the calculated density was 1.436 g/cm^3 and F(000), 2336 e$^-$.

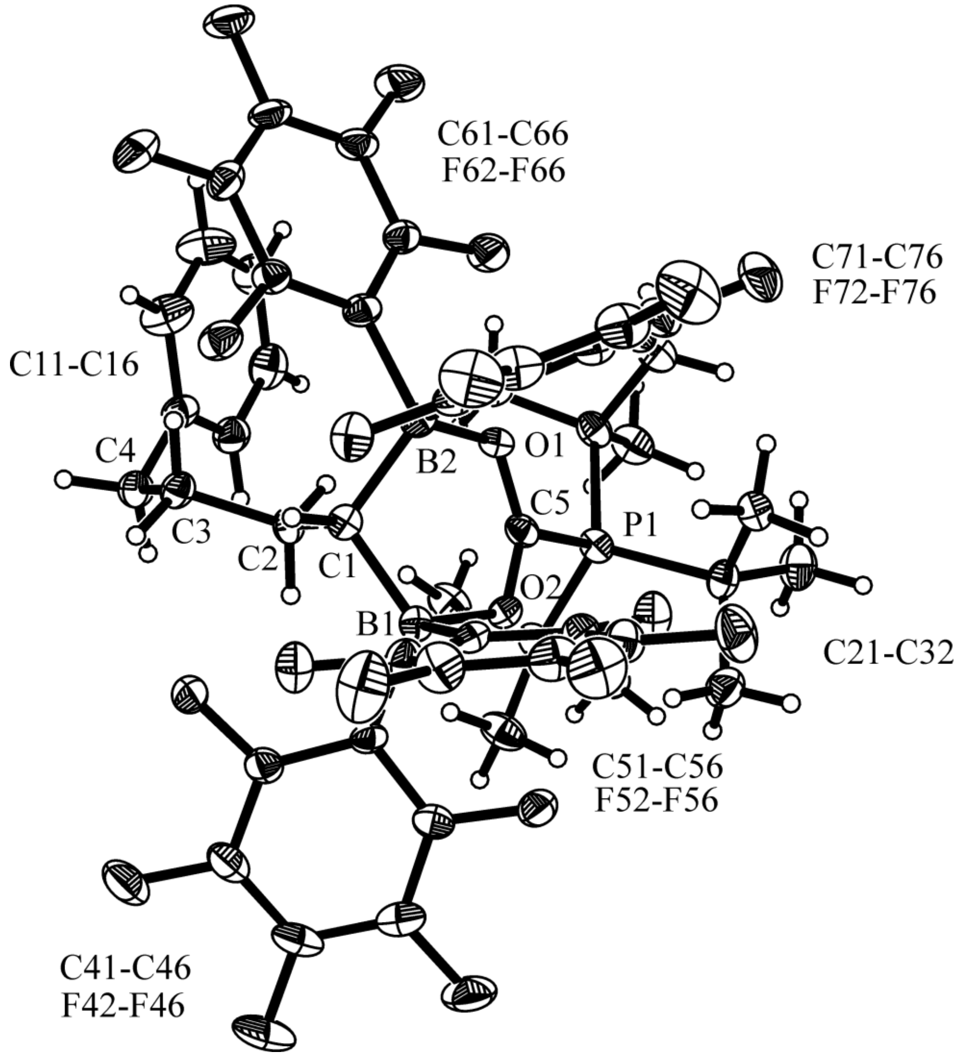
C61-C66
F62-F66
C71-C76
F72-F76
C11-C16
C4
B2
O1
C5
P1
C3
C2
C1
O2
B1
C21-C32
C51-C56
F52-F56
C41-C46
F42-F46

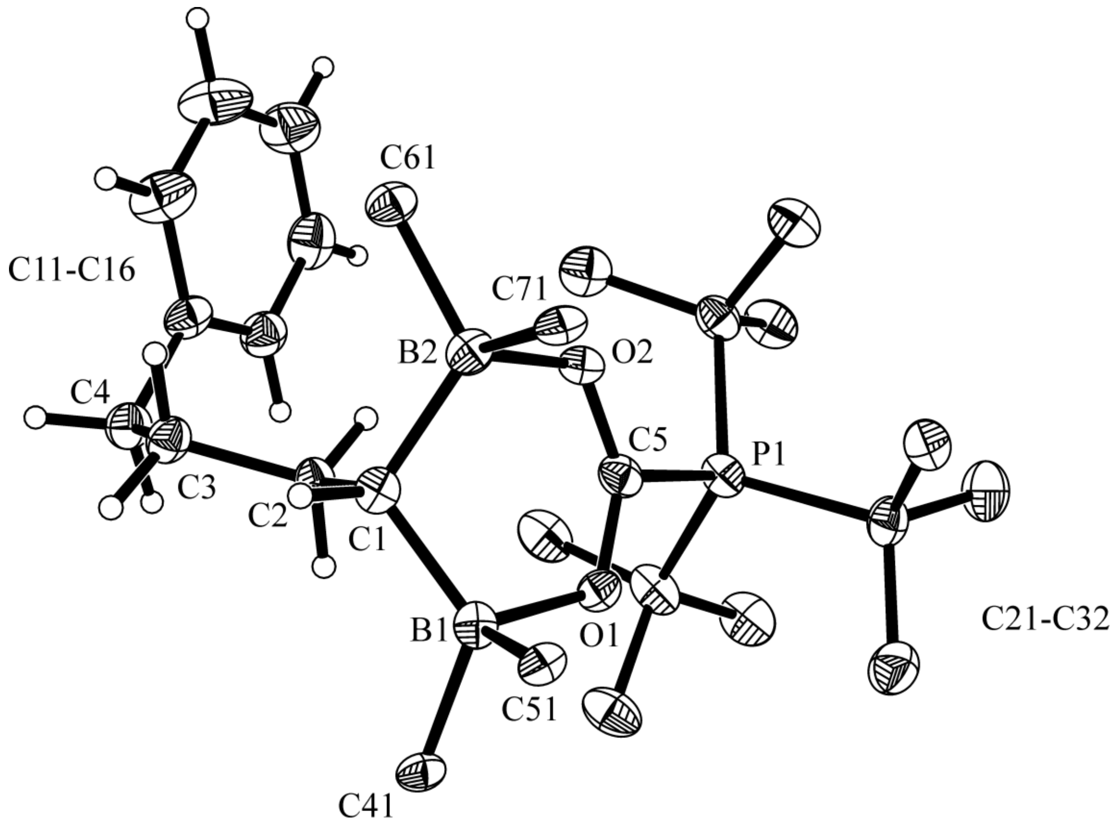
C61
C11-C16
C71
B2
O2
C4
C5
P1
C3
C2
C1
B1
O1
C21-C32
C51
C41

*2nd Experiment (NMR scale; in situ; characterization of compound **6a** by NMR):* Compound **6a** (41.1 mg, 0.05 mmol), *tri-tert*-butylphosphane (10.1 mg, 0.05 mmol) and CD_2Cl_2 (0,8 mL) were successively added into a J Young NMR tube. Then the reaction mixture was exposed to CO_2 (2.0 bar) at room temperature and shaken for 2 hours. Subsequently the resulting colourless solution was characterized by NMR experiments.

^{1}H NMR (600 MHz, 299 K, CD_2Cl_2): δ 7.13 (m, 2H, *m*-Ph), 7.07 (m, 1H, *p*-Ph), 6.89 (m, 2H, *o*-Ph), 2.46 (m, 2H, 4-CH_2), 1.84 (m, 1H, 1-CH), 1.78 (m, 2H, 3-CH_2), 1.67 (d, $^3J_{PH}$ = 15.5 Hz, 27H, t-Bu), 1.26 (m, 2H, 2-CH_2).

^{13}C{^{1}H} NMR (151 MHz, 299 K, CD_2Cl_2): δ 172.7 (d, $^1J_{PC}$ = 92.6 Hz, C=O), 143.1 (*i*-Ph), 128.5 (*o*-Ph), 128.4 (*m*-Ph), 125.7 (*p*-Ph), 43.3 (d, $^1J_{PC}$ = 16.7 Hz, t-Bu), 37.4 (4-CH_2), 32.4 (3-CH_2), 31.6 (2-CH_2), 30.7 (t-Bu), 18.0 (br, 1-CH), [C_6F_5 not listed].

^{1}H, ^{13}C GHSQC (600 MHz / 151 MHz, 299 K, CD_2Cl_2): δ^{1}H/δ^{13}C 7.13 / 128.4 (*m*-Ph), 7.07 / 125.7 (*p*-Ph), 6.89 / 128.5 (*o*-Ph), 2.46 / 37.4 (4-CH_2), 1.84 / 18.0 (1-CH), 1.78 / 32.4 (3-CH_2), 1.67 / 30.7 (t-Bu), 1.26 / 31.6 (2-CH_2).

^{19}F NMR (564 MHz, 299 K, CD_2Cl_2): δ -129.0, -131.4 (each br, each 2F, *o*-C_6F_5), -159.8, -160.2 (each br, each 1F, *p*-C_6F_5), -165.4, -165.6 (each br, each 2F, *m*-C_6F_5).

^{10}B{^{1}H} NMR (54 MHz, 299 K, CD_2Cl_2): δ 10.8 ($\nu_{1/2}$ ~ 750 Hz).

^{31}P{^{1}H} NMR (243 MHz, 299 K, CD_2Cl_2): δ 60.3 ($\nu_{1/2}$ ~ 20 Hz).

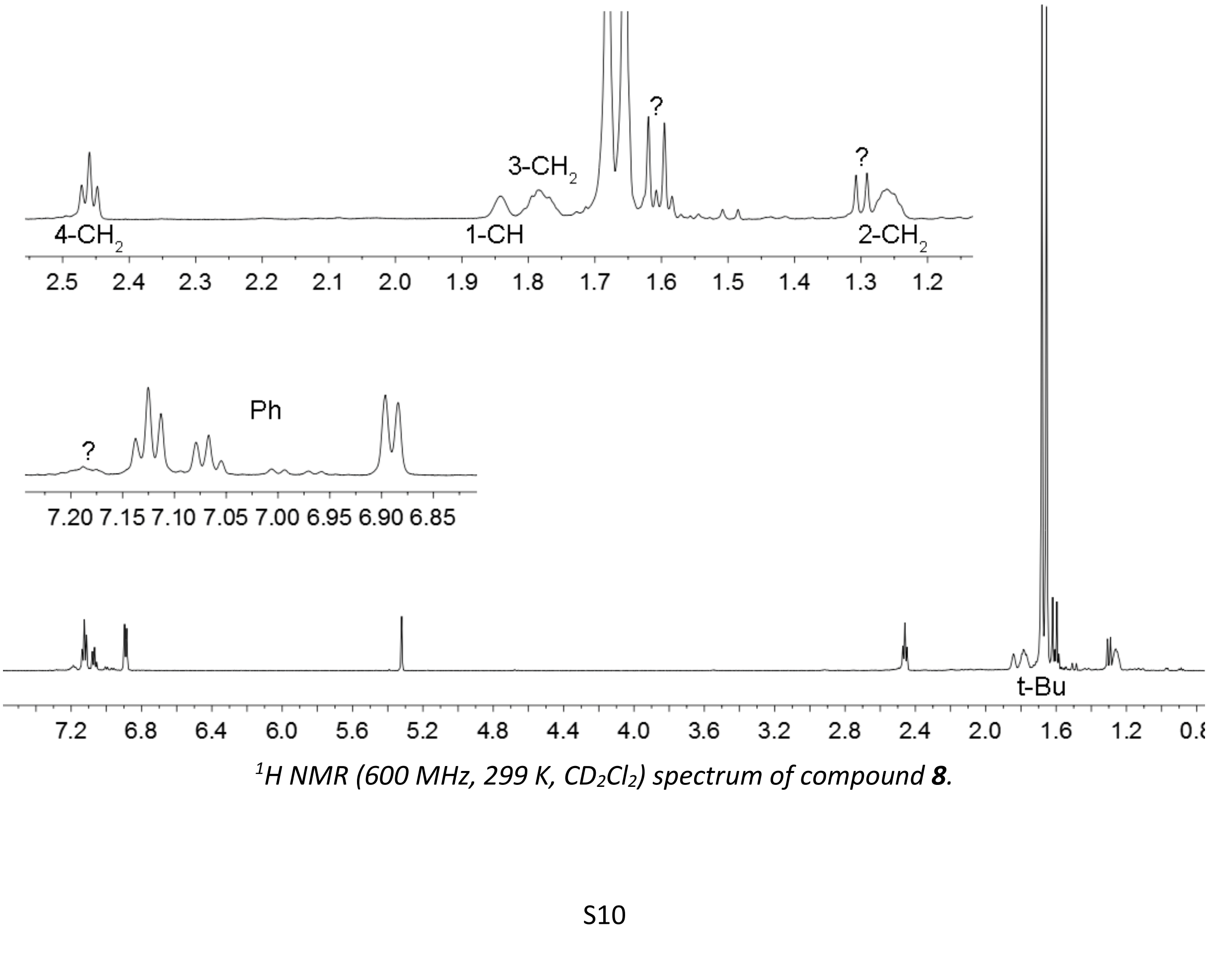

*^{1}H NMR (600 MHz, 299 K, CD_2Cl_2) spectrum of compound **8**.*

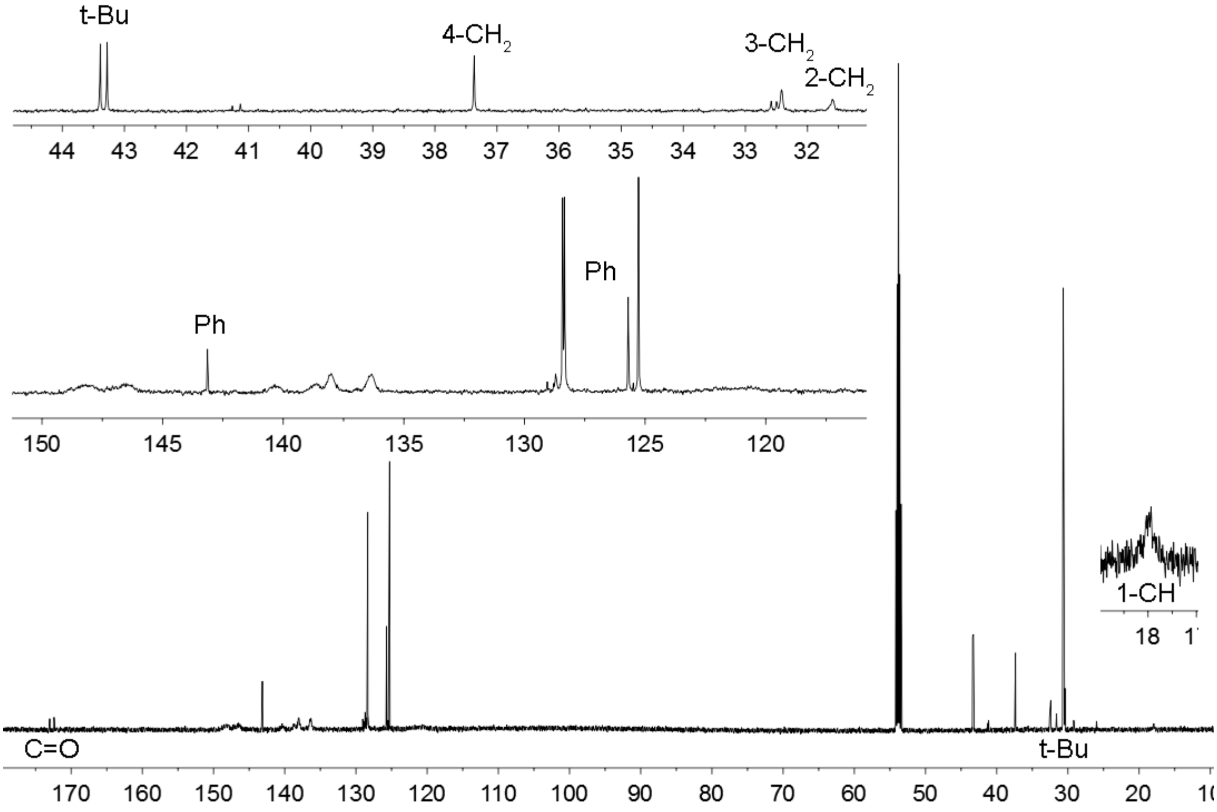

$^{13}C\{^{1}H\}$ *NMR (150 MHz, 299 K, CD_2Cl_2) spectrum of compound* ***8***.

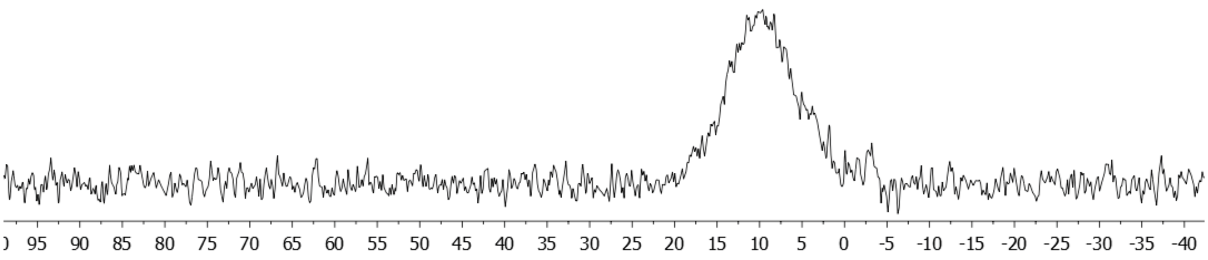

$^{10}B\{^{1}H\}$ *NMR (54 MHz, 299 K, CD_2Cl_2) spectrum of compound* ***8***.

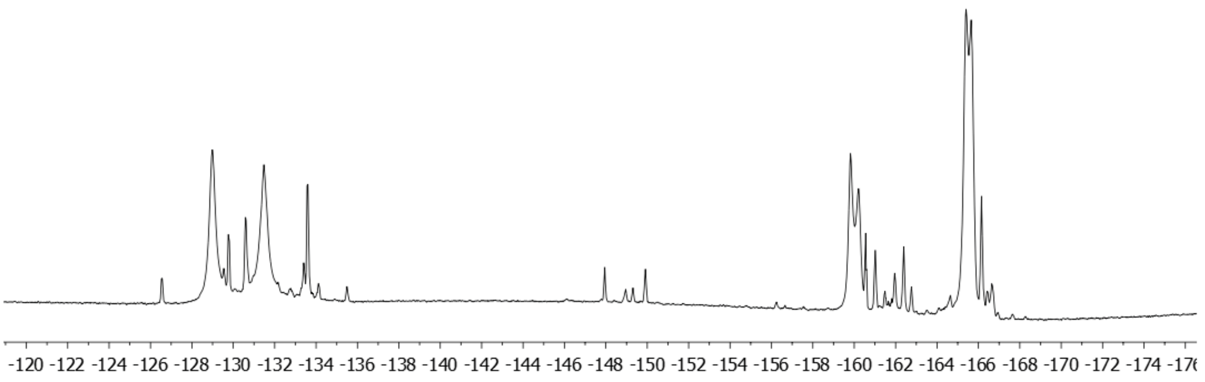

^{19}F *NMR (470 MHz, 299 K, CD_2Cl_2) spectrum of compound* ***8***.

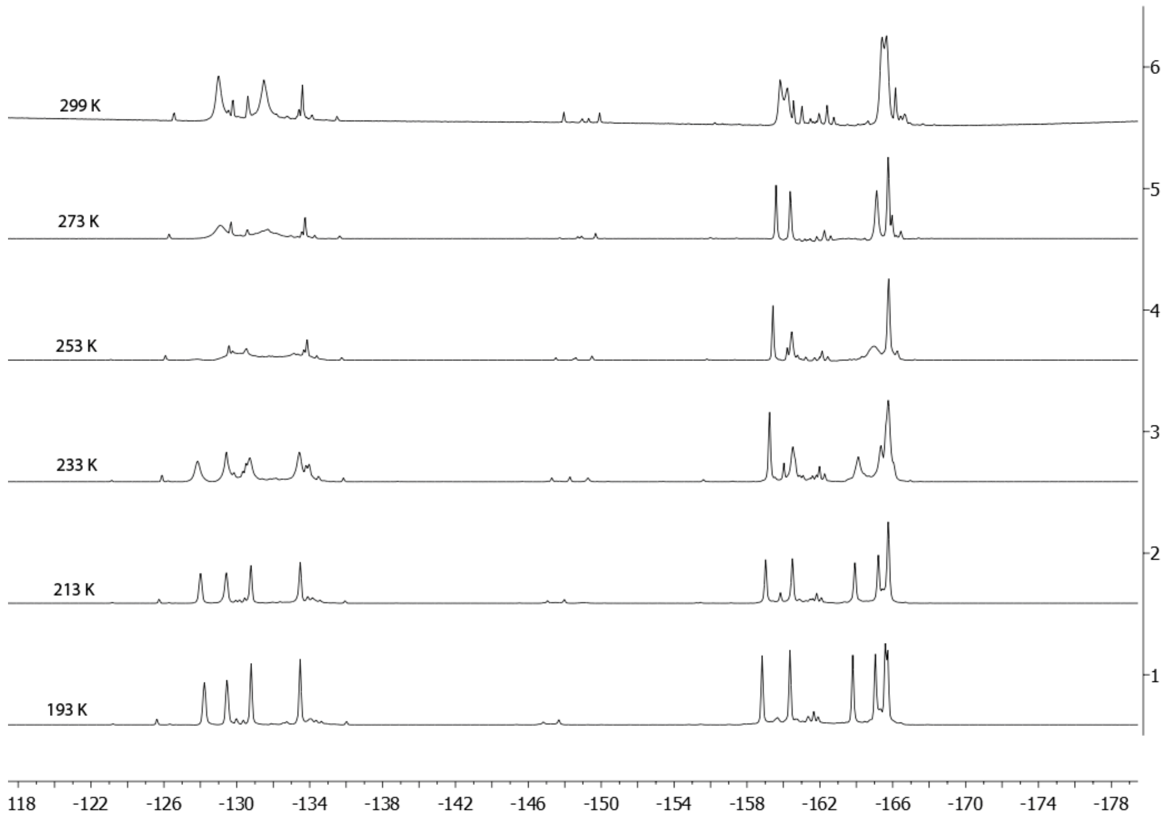

^{19}F NMR (470 MHz, CD_2Cl_2) spectra of compound **8** at variable temperatures.

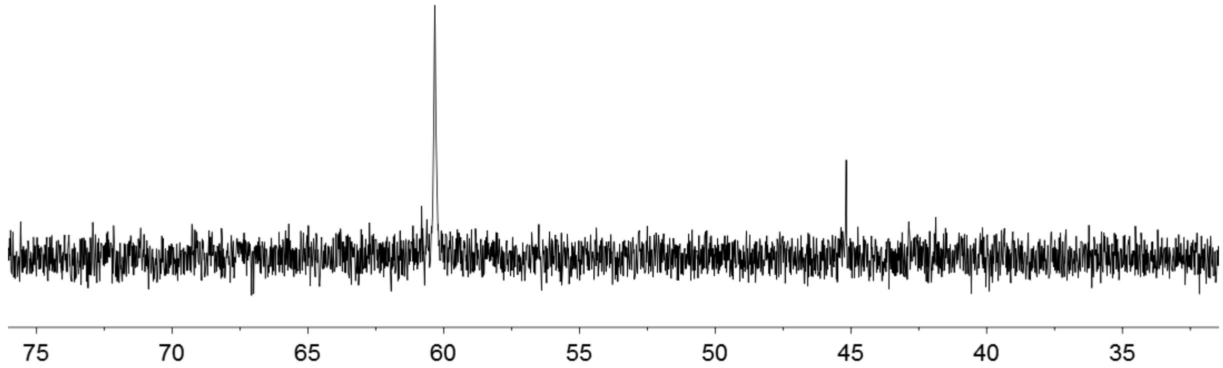

$^{31}P\{^1H\}$ NMR (243 MHz, 299 K, CD_2Cl_2) spectrum of compound **8**.

Preparation of compound *cis*-**9**:

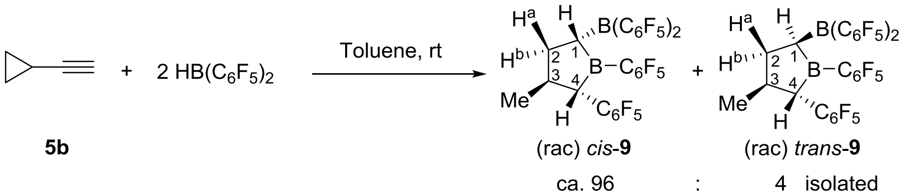

A solution of compound **5b** (33.0 mg, 0.50 mmol) in toluene (1.0 mL) was added to a suspension of bis(pentafluorophenyl)borane (345 mg, 1.00 mmol) and toluene (3.0 mL). After stirring the reaction mixture at room temperature for 1 hour, the solution was separated from the resulting suspension by cannula filtration. Then all volatiles of the filtrate were removed in vacuo to give a yellow oil, which was dissolved in pentane (2.5 mL) and stored at -35 °C overnight. The precipitated pale yellow solid was isolated by filtration and washed with cold pentane (2 × 0.5 mL). Removal of all volatiles in vacuo gave a pale yellow solid (253 mg, 0.34 mmol, 67%).

Melting point: 109 °C.

Anal. Calc. for $C_{29}H_8B_2F_{20}$: C, 45.95; H, 1.06. Found: C, 45.74; H, 1.07.

A solution of the yellow solid in C_6D_6 showed a mixture of compounds *cis*-**9** and *trans*-**9** [ratio ca. 96 : 4 (^{1}H)]. Compounds *cis*-**9**:

^{1}H NMR (500 MHz, 299 K, C_6D_6): δ 3.18 (m, 1H, 1-CH), 2.53 (m, 1H, 4-CH), 2.24 (m, 1H, 2-CH_2), 2.09 (m, 1H, 3-CH), 1.55 (m, 1H, 2-CH_2), 0.80 (d, $^3J_{HH}$ = 6.2 Hz, 3H, CH_3).

^{13}C{^{1}H} NMR (126 MHz, 299 K, C_6D_6): δ 50.6 (br, 1-CH), 46.4 (br, 4-CH), 42.8 (3-CH), 35.9 (2-CH_2), 19.3 (CH_3), [C_6F_5 not listed].

^{1}H, ^{13}C GHSQC (500 MHz / 126 MHz, 299 K, C_6D_6): δ^{1}H/ δ^{13}C 3.18 / 50.6 (1-CH), 2.53 / 46.4 (4-CH), 2.09 / 42.8 (3-CH), 1.55, 2.24 / 35.9 (2-CH_2), 0.80 / 19.3 (CH_3).

^{19}F NMR (470 MHz, 299 K, C_6D_6): δ -127.3 (m, 2F, *o*), -144.2 (tm, $^3J_{FF}$ = 21.3 Hz, 1F, *p*), -160.2 (m, 2F, *m*)(BC_6F_5)[Δδ$^{19}F_{m,p}$ = 16.0]; -130.6 (m, 4F, *o*), -146.7 (tm, $^3J_{FF}$ = 20.9 Hz, 2F, *p*), -160.6 (m, 4F, *m*)($B(C_6F_5)_2$)[Δδ$^{19}F_{m,p}$ = 13.9]; -142.7 (m, 2F, *o*), -156.5 (t, $^3J_{FF}$ = 21.3 Hz, 1F, *p*), -162.1 (m, 2F, *m*)(C_6F_5)[Δδ $^{19}F_{m,p}$ = 5.6].

^{11}B{^{1}H} NMR (160 MHz, 299 K, C_6D_6): δ 78.0 ($ν_{1/2}$ ~ 1500 Hz), 67.6 ($ν_{1/2}$ ~ 1500 Hz).

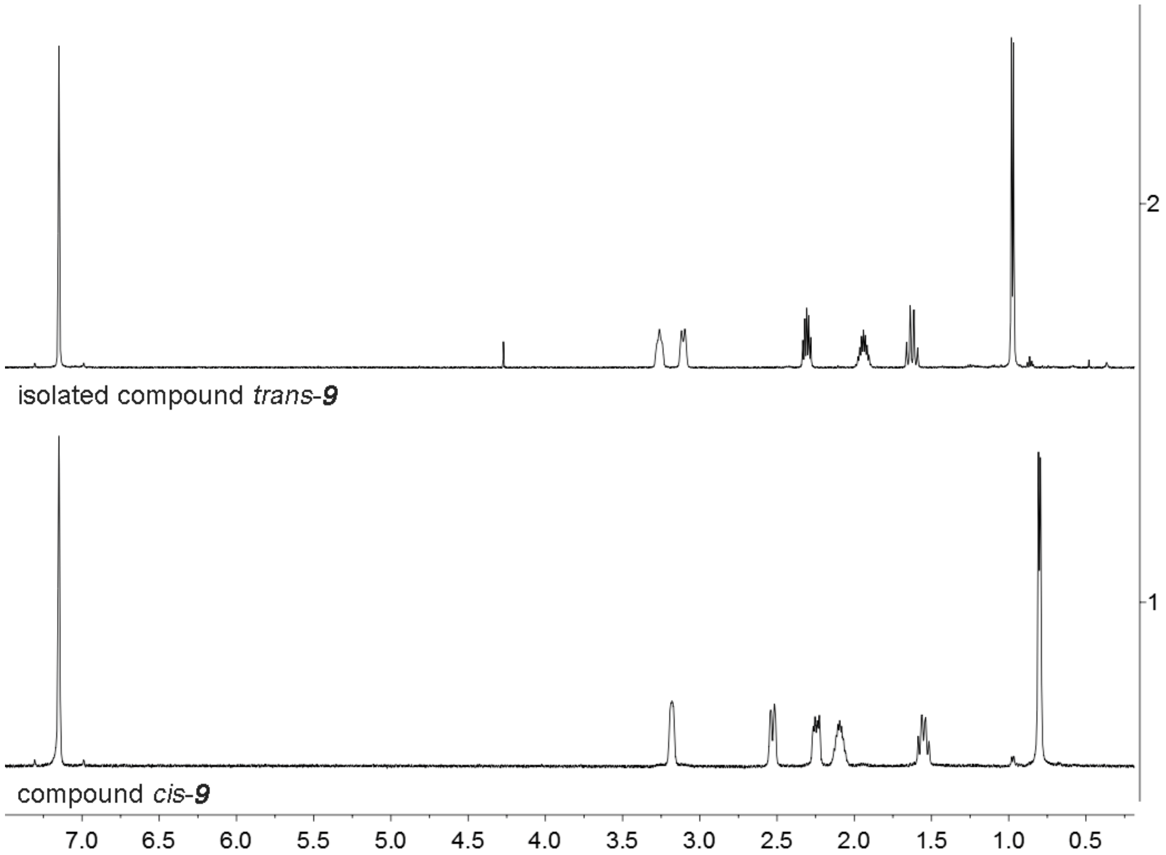

*^{1}H NMR (500 MHz, 299 K, C_6D_6) spectra of (1) compound cis-***9** (admixed with *trans*-**9**) and (2) isolated compound *trans*-**9** (see below).

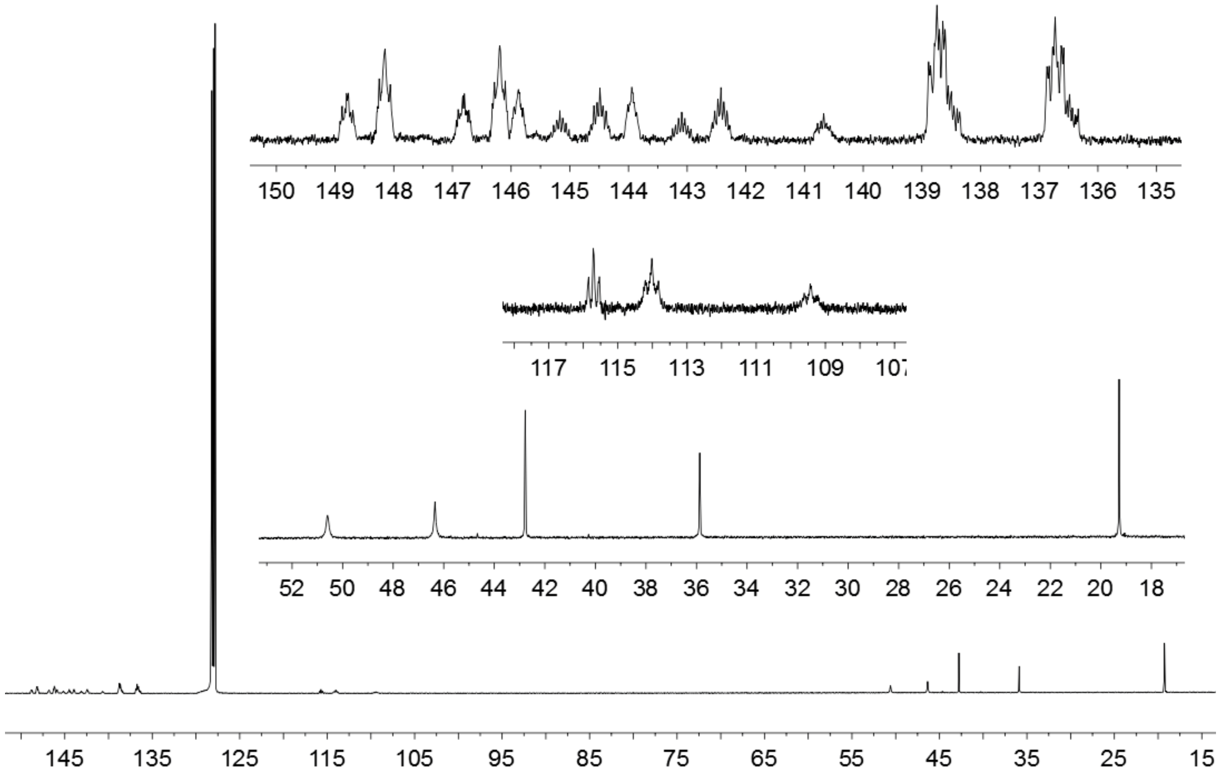

*^{13}C{^{1}H} NMR (126 MHz, 299 K, C_6D_6) spectrum of compound cis-***9**.

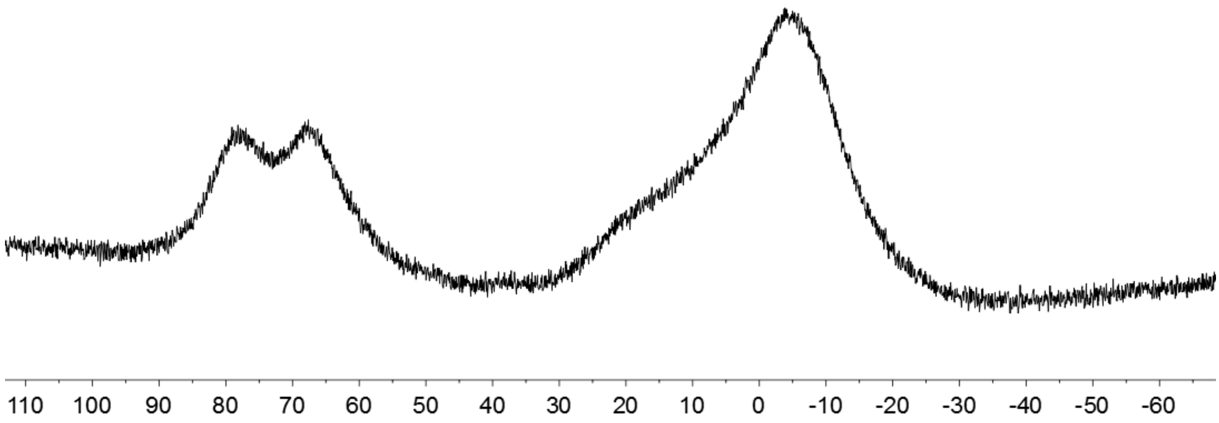

*$^{11}B\{^{1}H\}$ NMR (160 MHz, 299 K, C_6D_6) spectrum of compound cis-***9***.*

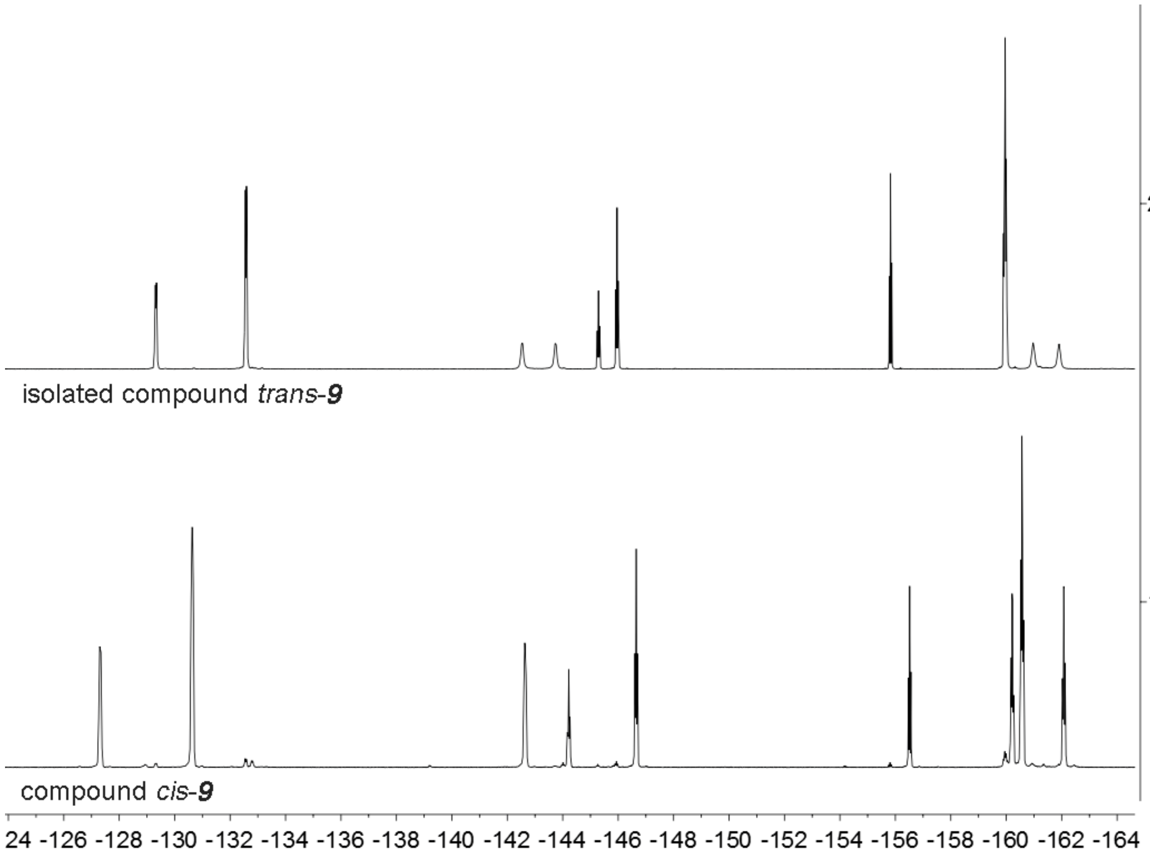

*^{19}F NMR (470 MHz, 299 K, C_6D_6) spectra of (1) compound cis-***9** (admixed with *trans*-**9**) and (2) isolated compound *trans*-**9** (see below).

Crystals of compound *cis*-**9** suitable for the X-ray crystal structure analysis were obtained from a solution of the yellow solid in pentane at -35 °C.

X-ray crystal structure analysis of compound *cis*-9: A colorless needle-like specimen of $C_{29}H_8B_2F_{20}$, approximate dimensions 0.030 mm x 0.070 mm x 0.250 mm, was used for the X-ray crystallographic analysis. The X-ray intensity data were measured. A total of 1402 frames were collected. The total exposure time was 23.52 hours. The frames were integrated with the Bruker SAINT software package using a wide-frame algorithm. The integration of the data using a monoclinic unit cell yielded a total of 19753 reflections to a maximum θ angle of 68.05° (0.83 Å resolution), of which 4950 were independent (average redundancy 3.991, completeness = 98.2%, R_{int} = 13.46%, R_{sig} = 11.84%) and 2616 (52.85%) were greater than 2σ(F^2). The final cell constants of a = 16.4031(16) Å, b = 6.7783(6) Å, c = 25.0981(19) Å, β = 98.450(5)°, volume = 2760.2(4) $Å^3$, are based upon the refinement of the XYZ-centroids of 1944 reflections above 20 σ(I) with 5.446° < 2θ < 133.6°. Data were corrected for absorption effects using the multi-scan method (SADABS). The ratio of minimum to maximum apparent transmission was 0.719. The calculated minimum and maximum transmission coefficients (based on crystal size) are 0.6570 and 0.9470. The structure was solved and refined using the Bruker SHELXTL Software Package, using the space group $P2_1/c$, with Z = 4 for the formula unit, $C_{29}H_8B_2F_{20}$. The final anisotropic full-matrix least-squares refinement on F^2 with 561 variables converged at R1 = 5.95%, for the observed data and wR2 = 15.64% for all data. The goodness-of-fit was 0.977. The largest peak in the final difference electron density synthesis was 0.295 $e^-/Å^3$ and the largest hole was -0.292 $e^-/Å^3$ with an RMS deviation of 0.068 $e^-/Å^3$. On the basis of the final model, the calculated density was 1.824 g/cm^3 and F(000), 1488 e^-.

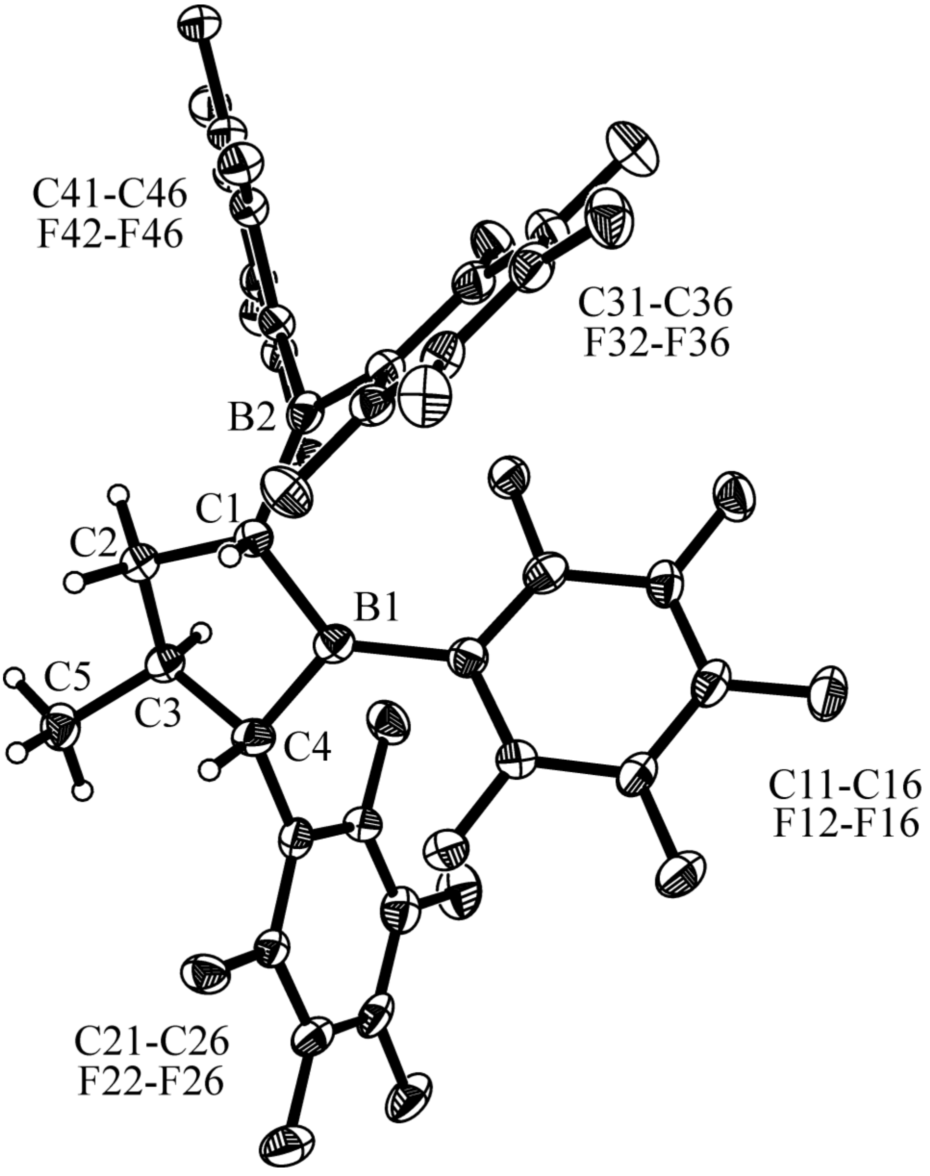
C41-C46
F42-F46
C31-C36
F32-F36
B2
C1
C2
B1
C5
C3
C4
C11-C16
F12-F16
C21-C26
F22-F26

Preparation of compound *trans*-**9**:

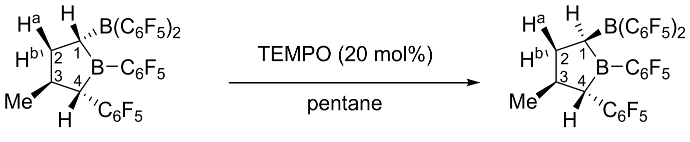

cis-**9** *trans*-**9**

TEMPO (16.6 mg, 0.11 mmol) was added to a solution of compound *cis*-**9** (400 mg, 0.53 mmol) in pentane (15 mL). After stirring the reaction mixture at r.t. for 4 days, the resulting suspension was concentrated to about 2.0 mL, and stored in the fridge (-35 °C) overnight. The precipitated white powder was isolated via cannula filtration, and washed with cold pentane (2 × 1.0 mL). Removal of all volatiles under reduced pressure gave product *trans*-**9** (296 mg, 0.39 mmol, 74%) as a white solid.

Melting point: 132 °C.

Anal. Calc. for $C_{29}H_8B_2F_{20}$: C, 45.95; H, 1.06. Found: C, 45.45; H, 0.95.

^{1}H NMR (500 MHz, 299 K, C_6D_6): δ 3.26 (m, 1H, 1-CH), 3.10 (m, 1H, 4-CH), 2.31 (m, 1H, 2-CH_2), 1.94 (m, 1H, 3-CH), 1.63 (m, 1H, 2-CH_2), 0.98 (d, $^3J_{HH}$ = 6.4 Hz, 3H, CH_3).

^{13}C{^{1}H} NMR (126 MHz, 299 K, C_6D_6): δ 56.4 (br, 1-CH), 45.8 (br, 4-CH), 44.7 (3-CH), 40.3 (2-CH_2), 19.1 (CH_3), [C_6F_5 not listed].

^{1}H, ^{13}C GHSQC (500 MHz / 126 MHz, 299 K, C_6D_6): δ^{1}H/ δ^{13}C 3.26 / 56.4 (1-CH), 3.10 / 45.8 (4-CH), 2.31, 1.63 / 40.3 (2-CH_2), 1.94 / 44.7 (3-CH), 0.98 / 19.1 (CH_3).

^{19}F NMR (470 MHz, 299 K, C_6D_6): δ -129.3 (m, 2F, *o*), -145.3 (tm, $^3J_{FF}$ = 20.8 Hz, 1F, *p*), -160.0 (m, 2F, *m*)(BC_6F_5)[Δδ$^{19}F_{m,p}$ = 14.7]; -132.6 (m, 4F, *o*), -146.0 (tm, $^3J_{FF}$ = 20.8 Hz, 2F, *p*), -160.0 (m, 4F, *m*)($B(C_6F_5)_2$)[Δδ$^{19}F_{m,p}$ = 14.0]; -142.5 (br, *o*), -143.8 (br, *o'*), -155.8 (t, $^3J_{FF}$ = 21.4 Hz, *p*), -161.0 (m, *m'*), -161.9 (m, *m*)(each 1F, C_6F_5)[Δδ $^{19}F_{m,p}$ = 5.2, 6.1].

^{11}B{^{1}H} NMR (160 MHz, 299 K, C_6D_6): δ 79.6 ($ν_{1/2}$ ~ 1500 Hz), 72.9 ($ν_{1/2}$ ~ 1500 Hz).

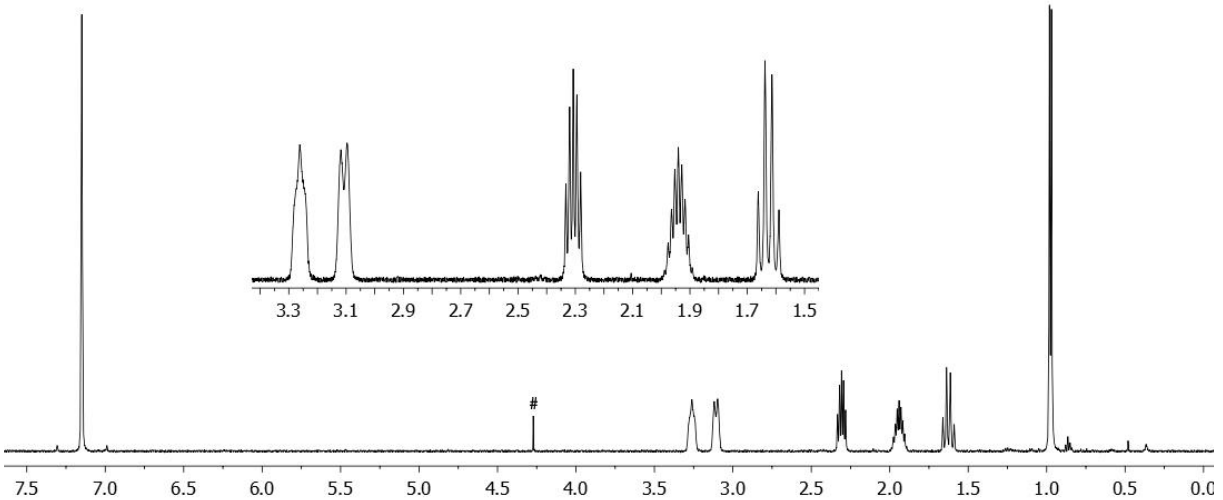

^{1}H NMR (500 MHz, 299 K, C_6D_6*) spectrum of compound trans*-**9**. (# *CH_2Cl_2*).

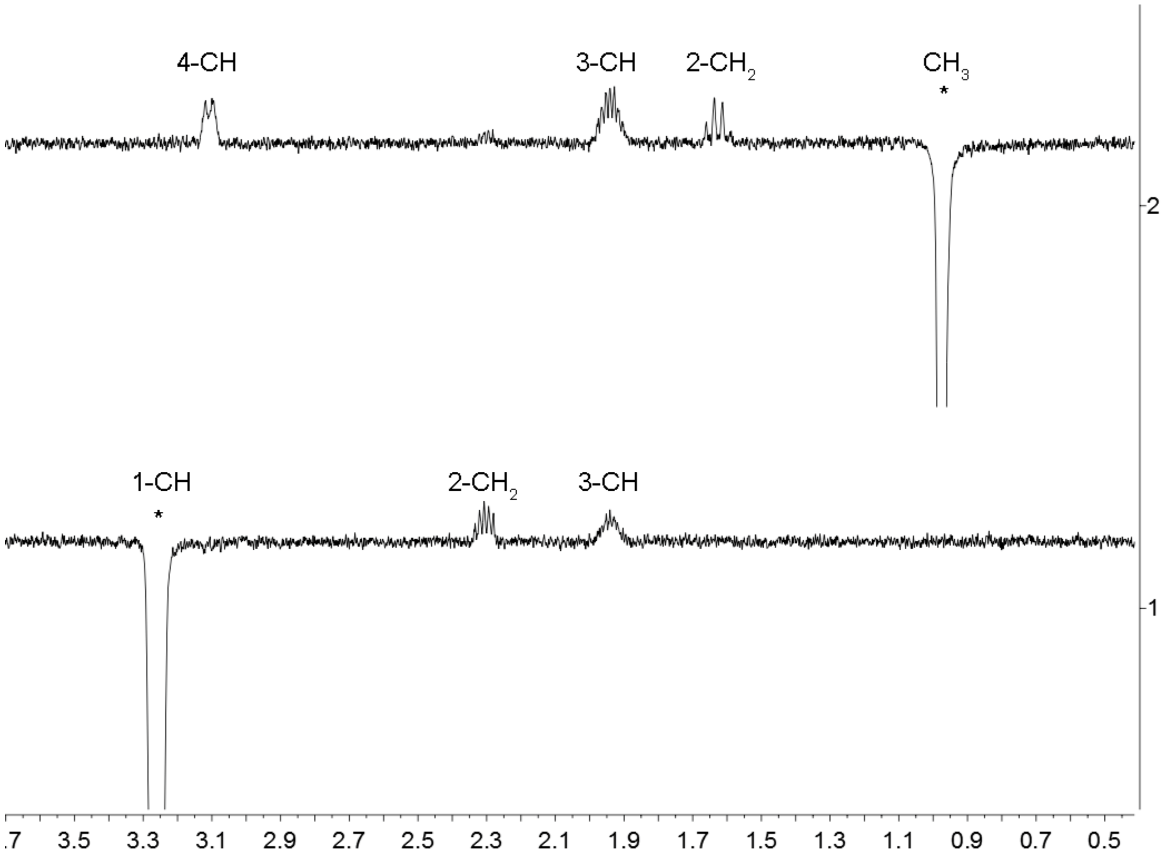

$^1H\{^1H\}$ NOEDIF NMR (500 MHz, 299 K, C_6D_6) spectra of compound trans-**9** [* irradiation point]

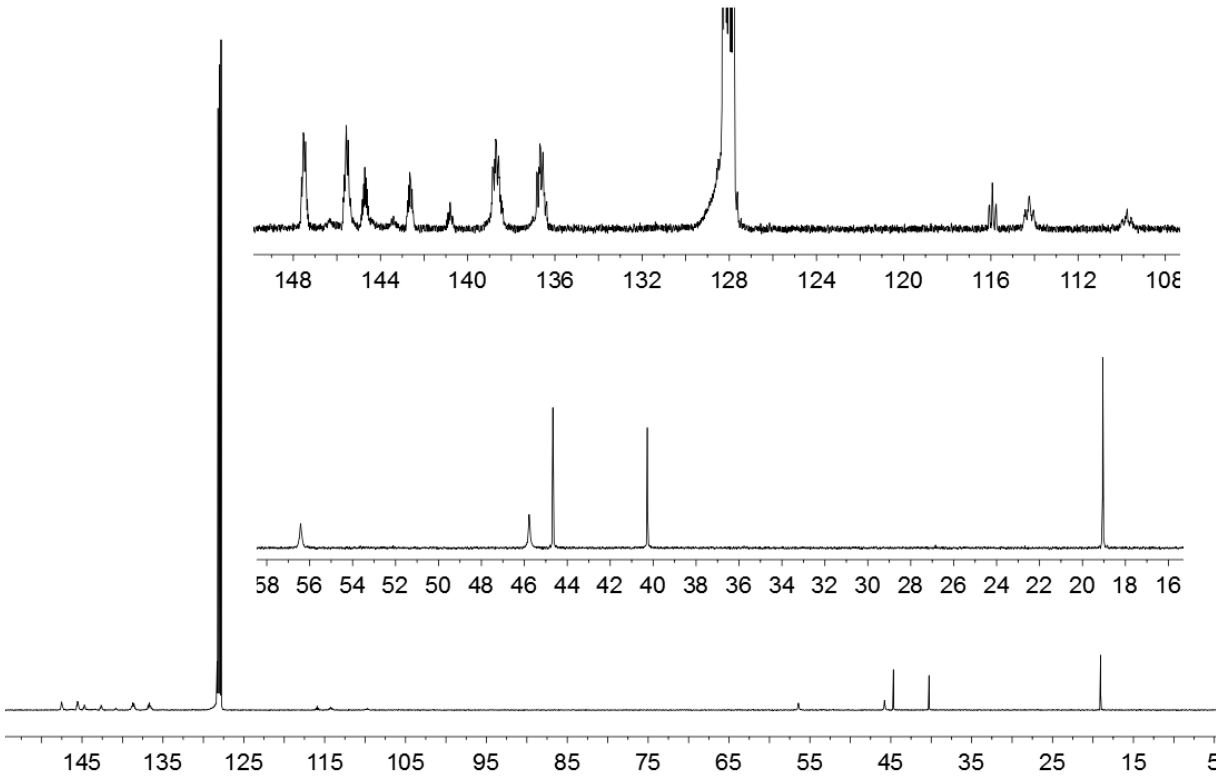

$^{13}C\{^1H\}$ NMR (126 MHz, 299 K, C_6D_6) spectrum of compound trans-**9**.

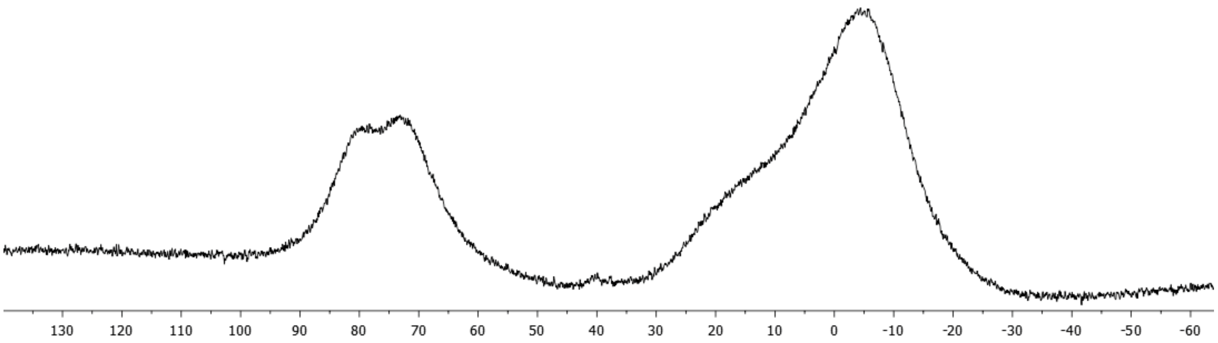

$^{11}B\{^{1}H\}$ NMR (160 MHz, 299 K, C_6D_6*) spectrum of compound trans-***9***.*

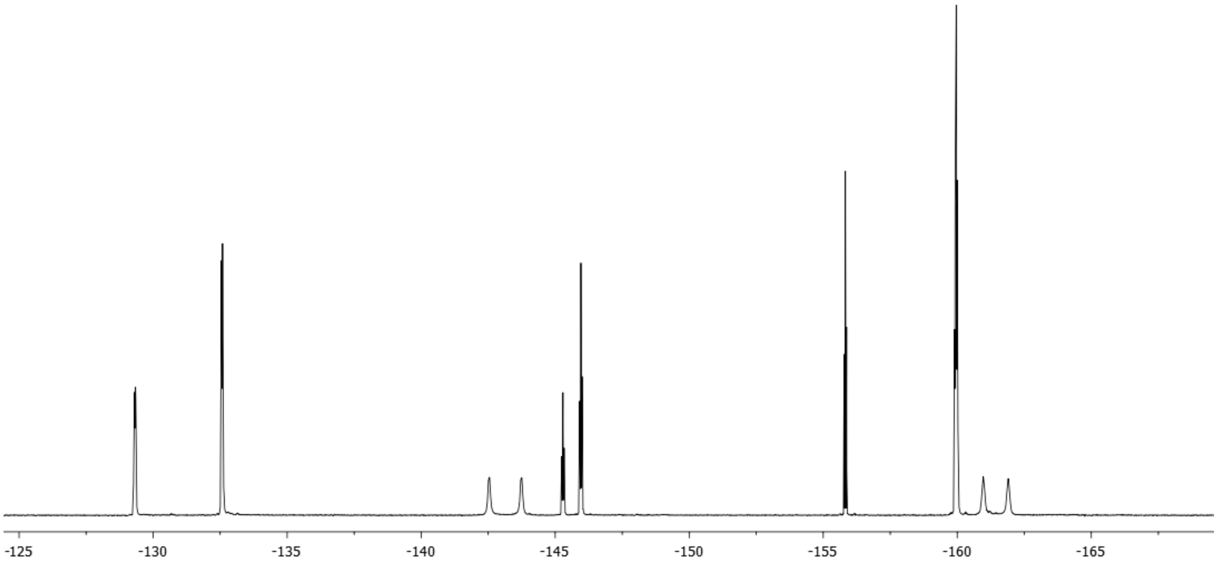

^{19}F NMR (470 MHz, 299 K, C_6D_6*) spectrum of compound trans-***9***.*

Crystals suitable for the X-ray crystal structure analysis were obtained from a solution of compound *trans*-**9** in pentane (1.5 mL) and CH_2Cl_2 (0.5 mL) at -35 °C.

X-ray crystal structure analysis of compound *trans*-9: A colorless prism-like specimen of $C_{29}H_8B_2F_{20}$, approximate dimensions 0.110 mm x 0.196 mm x 0.344 mm, was used for the X-ray crystallographic analysis. The X-ray intensity data were measured. A total of 1861 frames were collected. The total exposure time was 12.92 hours. The frames were integrated with the Bruker SAINT software package using a narrow-frame algorithm. The integration of the data using a monoclinic unit cell yielded a total of 58235 reflections to a maximum θ angle of 27.58° (0.77 Å resolution), of which 6337 were independent (average redundancy 9.190, completeness = 99.6%, R_{int} = 3.72%, R_{sig} = 2.14%) and 5229 (82.52%) were greater than 2σ(F^2). The final cell constants of a = 10.7742(5) Å, b = 9.7124(4) Å, c = 26.4759(11) Å, β = 97.3470(10)°, volume = 2747.8(2) $Å^3$, are based upon the refinement of the XYZ-centroids of 9489 reflections above 20 σ(I) with 5.213° < 2θ < 55.12°. Data were corrected for absorption effects using the multi-scan method (SADABS). The ratio of minimum to maximum

apparent transmission was 0.955. The calculated minimum and maximum transmission coefficients (based on crystal size) are 0.9340 and 0.9780. The final anisotropic full-matrix least-squares refinement on F^2 with 461 variables converged at R1 = 3.59%, for the observed data and wR2 = 8.85% for all data. The goodness-of-fit was 1.044. The largest peak in the final difference electron density synthesis was 0.293 $e^-/Å^3$ and the largest hole was -0.245 $e^-/Å^3$ with an RMS deviation of 0.055 $e^-/Å^3$. On the basis of the final model, the calculated density was 1.832 g/cm^3 and F(000), 1488 e^-.

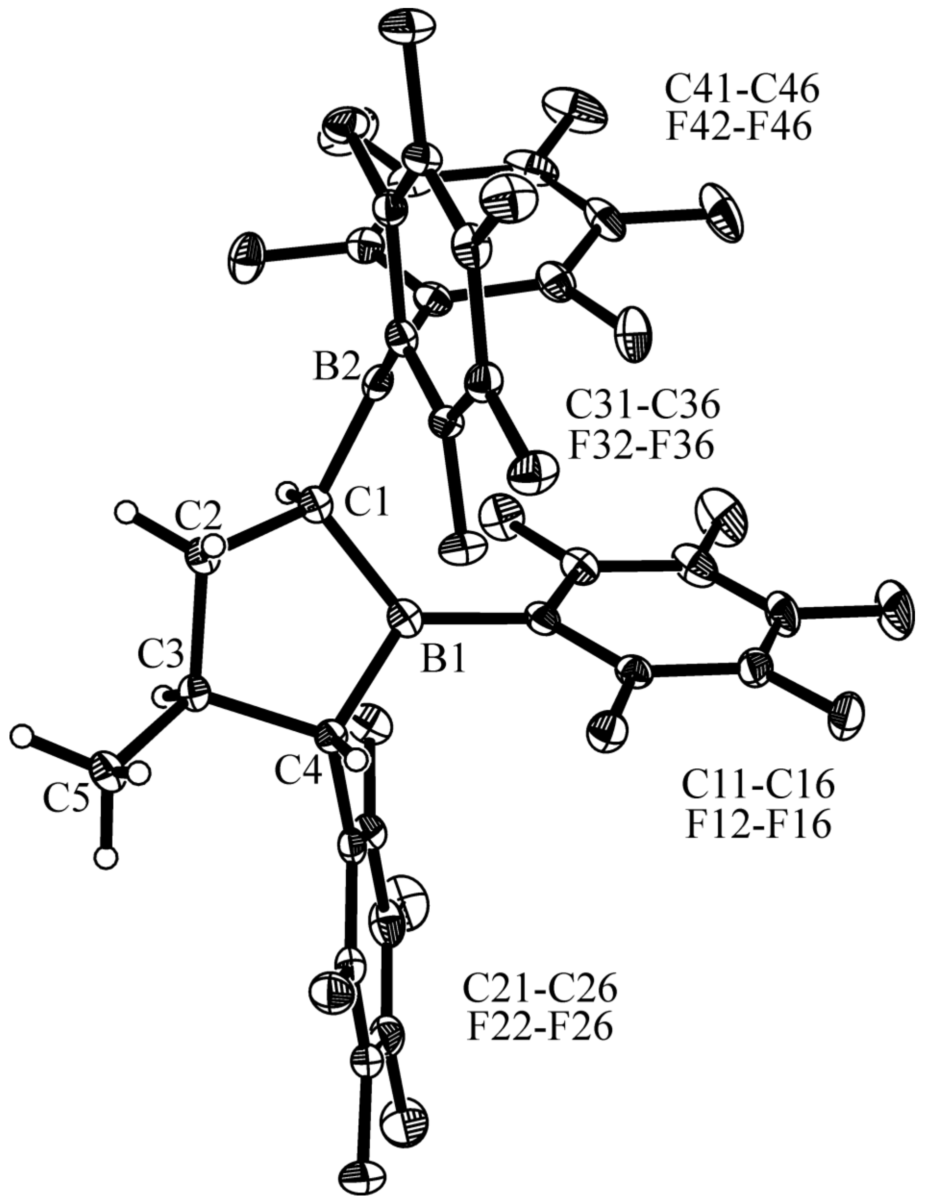

Preparation of compound *cis*-**12**:

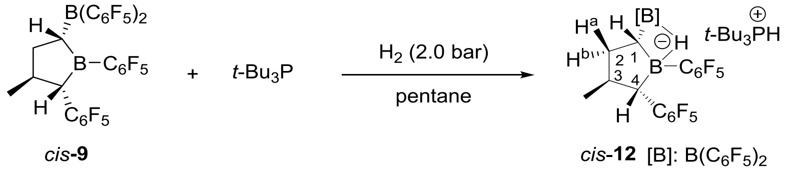

A solution of compound *cis*-**9** (cis/trans ≈ 96/4, vide supra) (113.7 mg, 0.15 mmol) and *tri-tert*-butylphosphane (30.3 mg, 0.15 mmol) in pentane (5.0 mL) was exposed to dihydrogen (2.0 bar) at room temperature and then stirred overnight. The formed white precipitate was collected by cannula filtration and washed with pentane (3 x 1 mL). After removal of all volatiles in vacuo, a white solid was obtained (101.4 mg, 0.11 mmol, 71%).

Melting point: 179 °C.

Anal. Calc. for $C_{41}H_{37}B_2F_{20}P$: C, 51.17; H, 3.88. Found: C, 50.96; H, 3.76.

A solution of the white solid in CD_2Cl_2 showed two compounds, which were assigned as *cis*-**12** and *trans*-**12** [ratio ca. 5 : 1 (^{19}F)]. *Compound cis-**12***:

^1H NMR (600 MHz, 299 K, CD_2Cl_2): δ 5.08 (d, $^1J_{PH}$ = 428.2 Hz, 1H, PH), 2.32 (m, 1H, 4-CH), 2.07, 1.66 (each m, each 1H, 2-CH_2), 1.68 (m, 1H, 3-CH), 1.68 (d, $^2J_{PH}$ = 15.8 Hz, 27H, t-Bu), 1.22 (m, 1H, 1-CH), 0.71 (d, $^3J_{HH}$ = 5.8 Hz, 3H, CH_3).

$^{13}C\{^1H\}$ NMR (151 MHz, 299 K, CD_2Cl_2): δ 43.3 (4-CH), 40.5 (2-CH_2), 38.1 (d, $^1J_{PC}$ = 26.8 Hz, t-Bu), 35.5 (t, J = 3.3 Hz, 3-CH), 30.4 (t-Bu), 20.8 (CH_3), 12.0 (br, 1-CH), [C_6F_5 not listed].

^1H, ^{13}C GHSQC (600 MHz / 151 MHz, 299 K, CD_2Cl_2): $δ^1H$/ $δ^{13}C$ 2.32 / 43.3 (4-CH), 2.07, 1.66 / 40.5 (2-CH_2), 1.68 / 35.5 (3-CH), 1.68 / 30.4 (t-Bu), 1.22 / 12.0 (1-CH), 0.71 / 20.8 (CH_3).

^{19}F NMR (564 MHz, 299 K, CD_2Cl_2): δ -128.7 (br, 1F), -130.3 (m, 2F), -132.9 (br, 1F), -133.2 (br m, 1F), -143.7 (m, 2F)(*o*-C_6F_5), -161.58 (t, $^3J_{FF}$ = 20.7 Hz), -161.60 (t, $^3J_{FF}$ = 19.8 Hz), -161.9 (t, $^3J_{FF}$ = 20.2 Hz), -165.2 (t, $^3J_{FF}$ = 22.0)(each 1F, *p*-C_6F_5), -165.9 (br, 1F), -166.1 (br, 1F), -166.3 (m, 2F), -166.7 (br, 2F), -166.9 (br m, 2F)(*m*-C_6F_5).

$^{11}B\{^1H\}$ NMR (192 MHz, 299 K, CD_2Cl_2): δ -14.5 ($ν_{1/2}$ ~ 350 Hz), -19.7 ($ν_{1/2}$ ~ 250 Hz).

^{11}B NMR (192 MHz, 299 K, CD_2Cl_2): δ -14.5 ($ν_{1/2}$ ~ 350 Hz), -19.7 ($ν_{1/2}$ ~ 250 Hz).

$^{31}P\{^1H\}$ NMR (243 MHz, 299 K, CD_2Cl_2): δ 60.63 ($ν_{1/2}$ ~ 7 Hz), 60.59 ($ν_{1/2}$ ~ 15 Hz)

^{31}P NMR (243 MHz, 299 K, CD_2Cl_2): δ 60.6 (dm, $^1J_{PH}$ ~ 428 Hz).

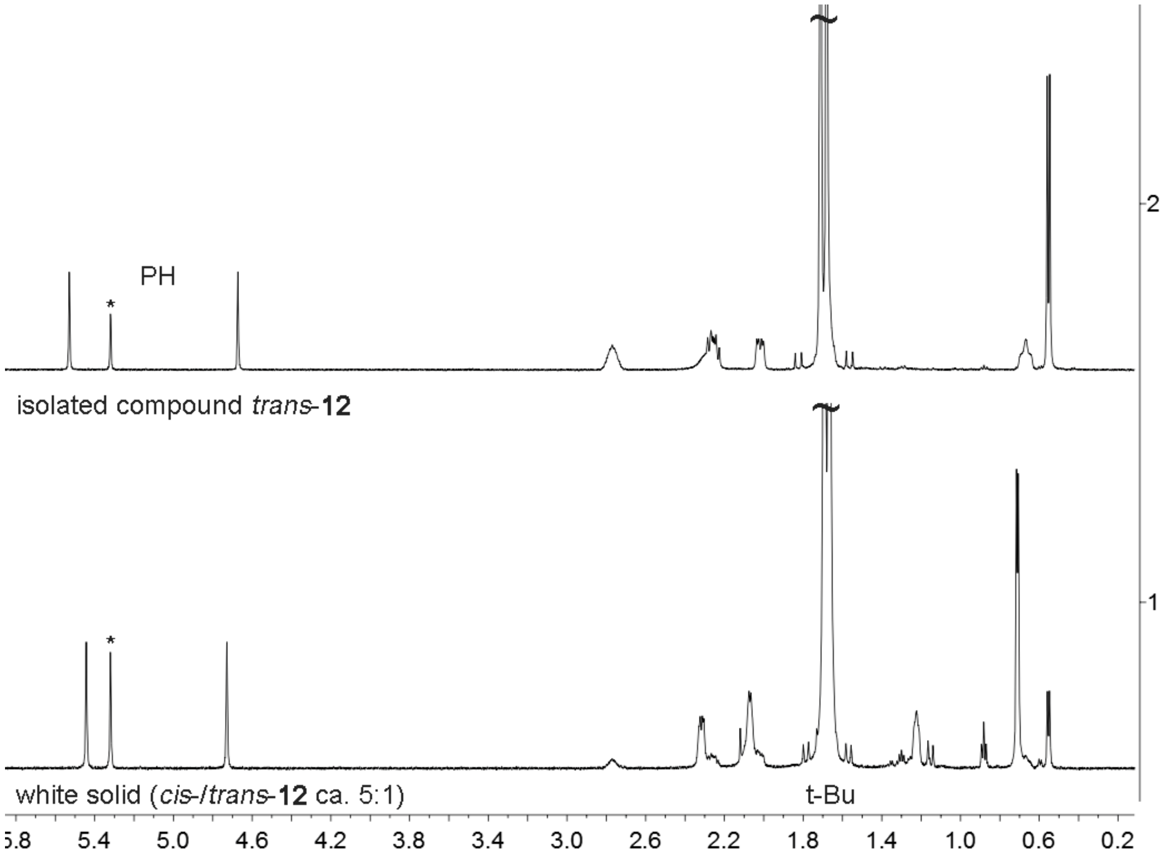

(1) ^{1}H NMR (600 MHz, 299 K, CD_2Cl_2**) spectrum of the white solid (cis-**12** / trans-**12** ca. 5 : 1) and (2) ^{1}H NMR (500 MHz, 299 K,* CD_2Cl_2**) spectrum of isolated compound trans-**12**.*

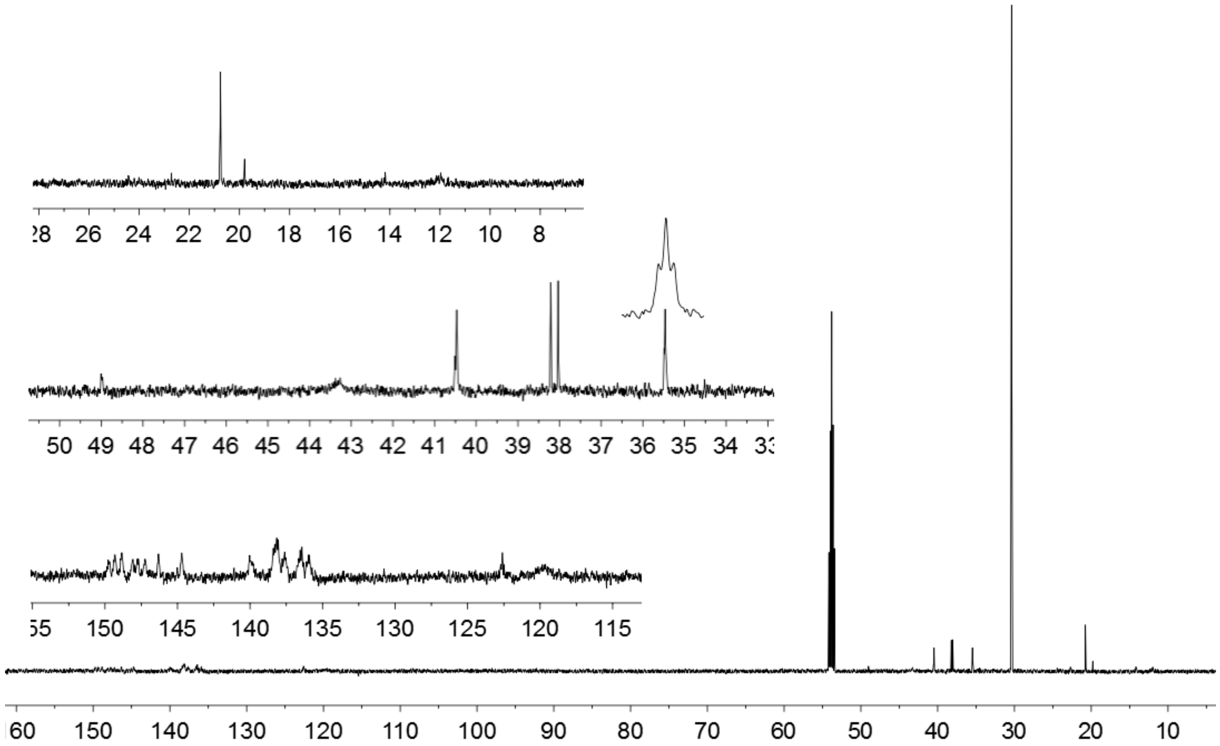

^{13}C{^{1}H} NMR (150 MHz, 299 K, CD_2Cl_2*) spectrum of the white solid (cis-**12** / trans-**12** ca. 5 : 1)*

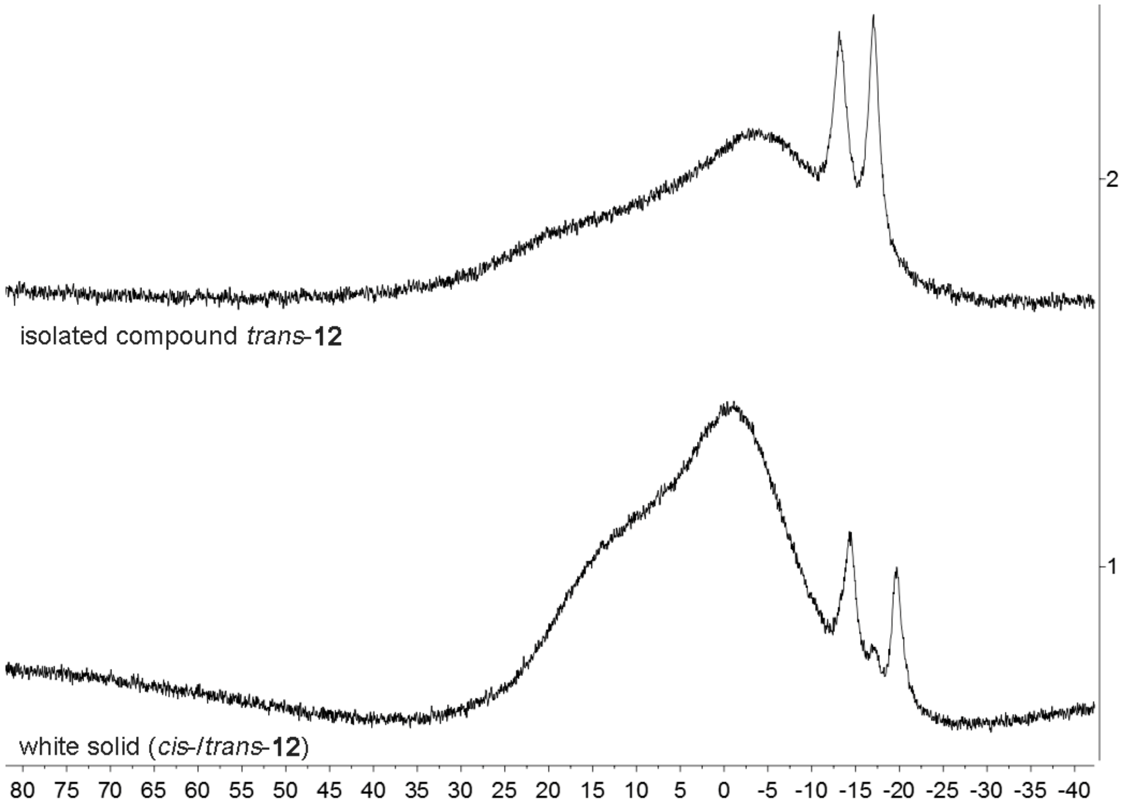

*(1) $^{11}B\{^{1}H\}$ NMR (192 MHz, 299 K, CD_2Cl_2) spectrum of the white solid (cis-**12** / trans-**12** ca. 5 : 1) and (2) $^{11}B\{^{1}H\}$ NMR (160 MHz, 299 K, CD_2Cl_2) spectrum of isolated compound trans-**12**.*

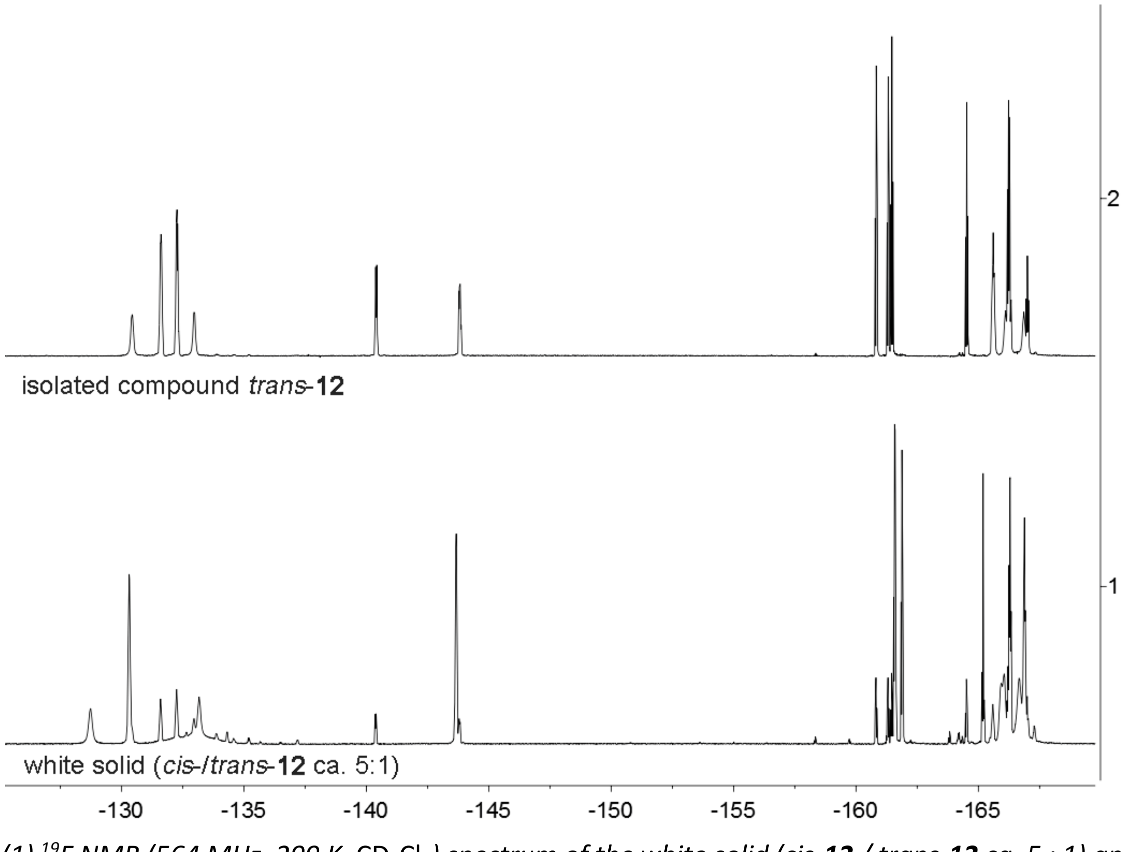

*(1) ^{19}F NMR (564 MHz, 299 K, CD_2Cl_2) spectrum of the white solid (cis-**12** / trans-**12** ca. 5 : 1) and (2) ^{19}F NMR (470 MHz, 299 K, CD_2Cl_2) spectrum of isolated compound trans-**12**.*

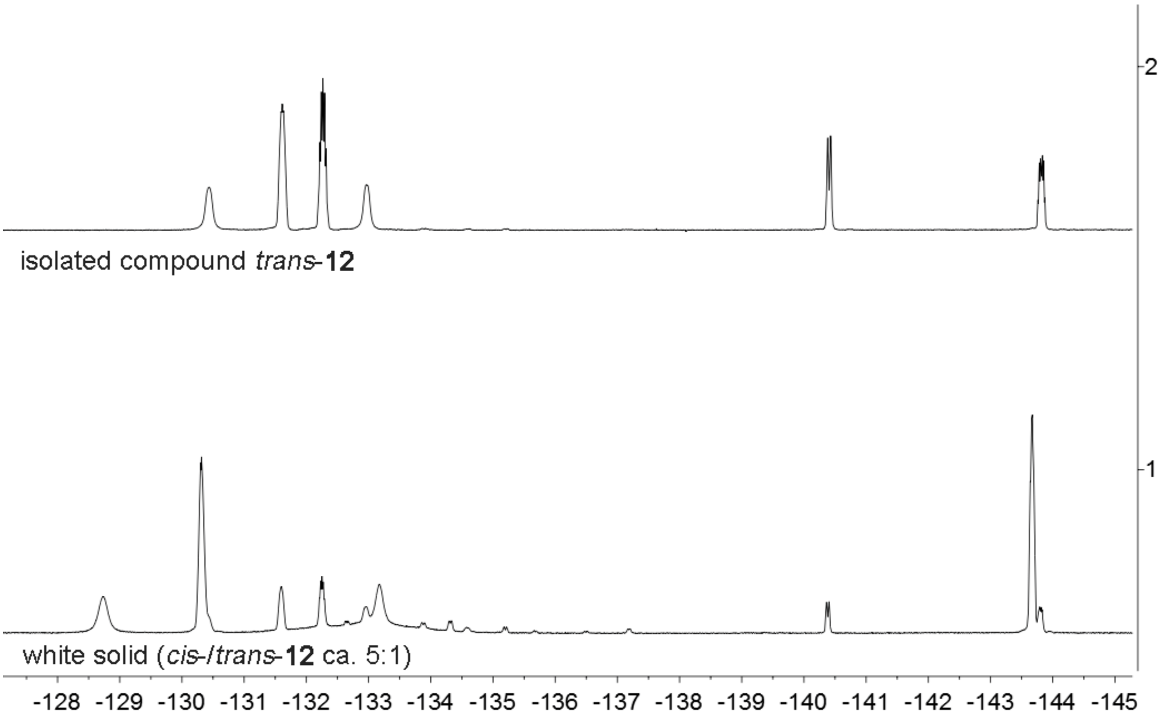

o-C_6F_5 range: (1) ^{19}F NMR (564 MHz, 299 K, CD_2Cl_2*) spectrum of the white solid (cis-**12** / trans-**12** ca. 5 : 1) and (2) ^{19}F NMR (470 MHz, 299 K,* CD_2Cl_2*) spectrum of isolated compound trans-**12**.*

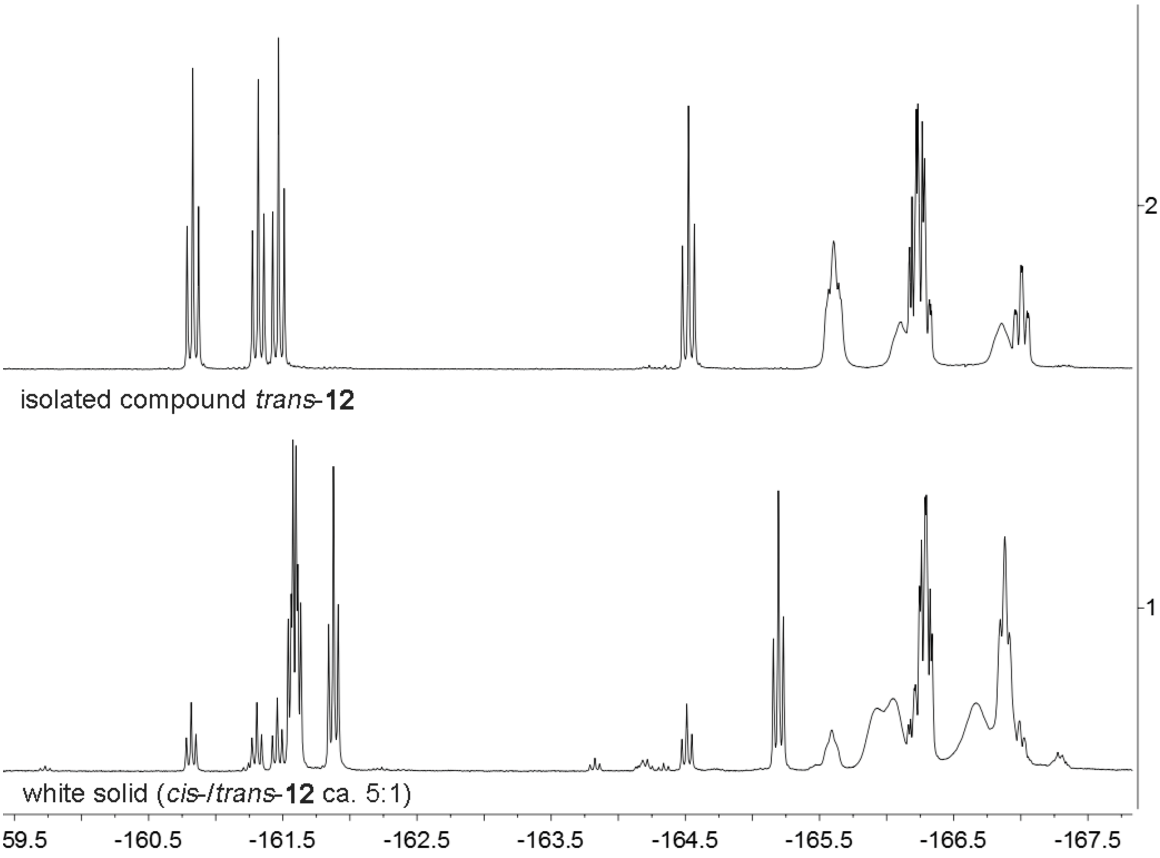

p,m-C_6F_5 range: (1) ^{19}F NMR (564 MHz, 299 K, CD_2Cl_2*) spectrum of the white solid (cis-**12** / trans-**12** ca. 5 : 1) and (2) ^{19}F NMR (470 MHz, 299 K,* CD_2Cl_2*) spectrum of isolated compound trans-**12**.*

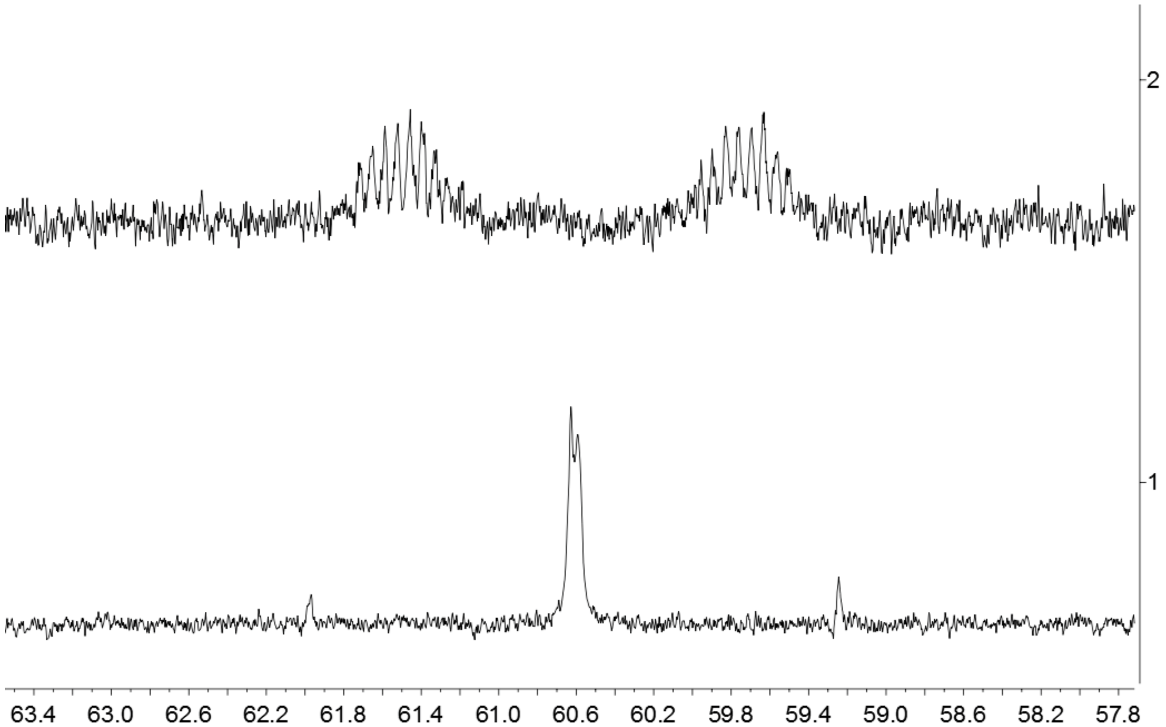

*(1) $^{31}P\{^1H\}$ and (2) ^{31}P NMR (243 MHz, 299 K, CD_2Cl_2) spectrum of the white solid (cis-**12** / trans-**12** ca. 5 : 1)*

Single crystals of compound *cis*-**12** suitable for the X-ray crystal structure analysis were obtained by slow diffusion of *n*-pentane into a solution of the white solid in dichloromethane at room temperature.

[Comment: 87% *cis*-**12** and 13% *trans*-**12** were found in the single crystal]

X-ray crystal structure analysis of compound *cis*-12: A colorless prism-like specimen of $C_{41}H_{37}B_2F_{20}P \cdot CH_2Cl_2$, approximate dimensions 0.149 mm x 0.177 mm x 0.269 mm, was used for the X-ray crystallographic analysis. The X-ray intensity data were measured. A total of 975 frames were collected. The total exposure time was 10.83 hours. The frames were integrated with the Bruker SAINT software package using a narrow-frame algorithm. The integration of the data using a monoclinic unit cell yielded a total of 8391 reflections to a maximum θ angle of 25.35° (0.83 Å resolution), of which 8391 were independent (average redundancy 1.000, completeness = 99.8%, R_{int} = 4.73%, R_{sig} = 2.09%) and 7094 (84.54%) were greater than $2\sigma(F^2)$. The final cell constants of a = 13.0097(5) Å, b = 21.1601(9) Å, c = 17.7134(7) Å, β = 110.0350(10)°, volume = 4581.2(3) $Å^3$, are based upon the refinement of the XYZ-centroids of 9922 reflections above 20 σ(I) with 5.092° < 2θ < 55.07°. Data were corrected for absorption effects using the multi-scan method (SADABS). The ratio of minimum to maximum apparent transmission was 0.934. The calculated minimum and maximum transmission coefficients (based on crystal size) are 0.9260 and 0.9580. The final anisotropic full-matrix least-squares refinement on F^2 with 893 variables converged at R1 = 6.41%, for the observed

data and wR2 = 16.41% for all data. The goodness-of-fit was 1.059. The largest peak in the final difference electron density synthesis was 0.992 $e^-/Å^3$ and the largest hole was -0.558 $e^-/Å^3$ with an RMS deviation of 0.069 $e^-/Å^3$. On the basis of the final model, the calculated density was 1.518 g/cm^3 and F(000), 2120 e^-.

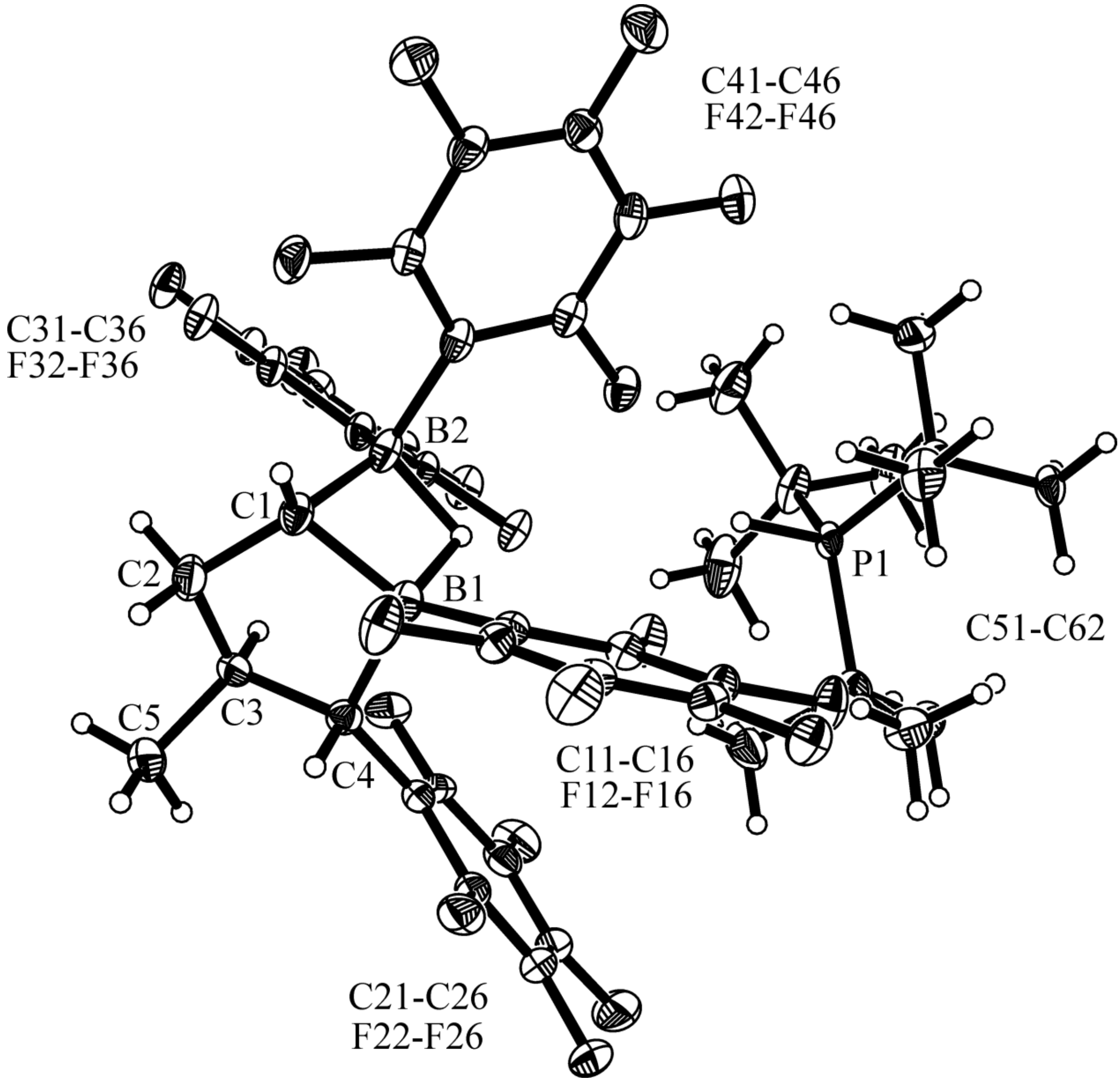

Preparation of compound *cis*-**13**:

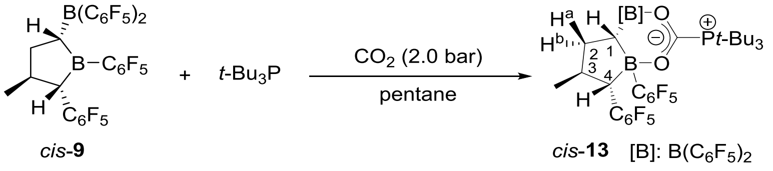

A solution of compound *cis*-**9** (cis/trans ≈ 96/4, vide supra)(113.7 mg, 0.15 mmol) and *tri-tert*-butylphosphane (30.3 mg, 0.15 mmol) in pentane (5.0 mL) was exposed to a CO_2 atmosphere (2.0 bar) and then stirred overnight at room temperature. The formed white precipitate was collected by cannula filtration and washed with pentane (3 x 1 mL). After removal of all volatiles in vacuo, a white solid was obtained (108.3 mg, 0.11 mmol, 73%).

Melting point: 99 °C.

IR (KBr, selective wavenumber): ṽ [cm-1] = 1598 (s, C-O asym. stretch).

Anal. Calc. for $C_{42}H_{35}B_2F_{20}O_2P$: C, 50.23; H, 3.51. Found: C, 50.07; H, 3.36.

A solution of the white solid in THF-d_8 showed a mixture of compounds: the major one was assigned as *cis*-**13**, a minor one as *trans*-**13** [ratio ca. 97 : 3 (^{19}F)].

^{1}H NMR (600 MHz, 233 K, THF-d_8): δ: 2.71 (br, 1H, 3-CH), 2.49 (m, 1H, 4-CH), 2.04 (m, 2H, 2-CH_2), 1.79 (d, $^3J_{PH}$ = 15.6 Hz, 27H, t-Bu), 1.70 (m, 1H, 1-CH), 1.07 (d, $^3J_{HH}$ = 6.2 Hz, 3H, CH_3).

^{13}C{^{1}H} NMR (151 MHz, 233 K, THF-d_8): δ 175.5 (d, $^1J_{PC}$ = 88.0 Hz, C=O), 45.0 (br, 4-CH), 43.4 (d, $^1J_{PC}$ = 16.3 Hz, t-Bu), 38.4 (br, 2-CH_2), 35.4 (br, 3-CH), 30.4 (br, t-Bu), 22.2 (CH_3), 19.5 (br, 1-CH), [C_6F_5 not listed].

^{1}H, ^{13}C GHSQC (600 MHz / 151 MHz, 233 K, CD_2Cl_2): δ^{1}H/ δ^{13}C 2.71 / 35.4 (3-CH), 2.49 / 45.0 (4-CH), 2.04 / 38.4 (2-CH_2), 1.79 / 30.4 (t-Bu), 1.70 / 19.5 (1-CH), 1.07 / 22.2 (CH_3).

^{19}F NMR (564 MHz, 233 K, THF-d_8): δ -130.1 (br), -132.4 (br), -134.7 (m), -141.8 (m)(each 2F, *o*), -159.8 (t, $^3J_{FF}$ = 20.8 Hz), -161.2 (t, $^3J_{FF}$ = 20.7 Hz), -162.6 (t, $^3J_{FF}$ = 20.6 Hz), -163.5 (t, $^3J_{FF}$ = 21.7 Hz)(each 1F, *p*), -165.8 (br, 2F), -166.2 (m, 3F), -167.0 (m, 3F)(*m*)(C_6F_5).

^{11}B{^{1}H} NMR (192 MHz, 233 K, THF-d_8): δ n.o. (overlap with "glass hill").

^{31}P{^{1}H} NMR (243 MHz, 233 K, THF-d_8): δ 60.5 ($ν_{1/2}$ ~ 5 Hz), [54.7 ($ν_{1/2}$ ~ 5 Hz, PH ($^1J_{PH}$ ~ 460 Hz)]

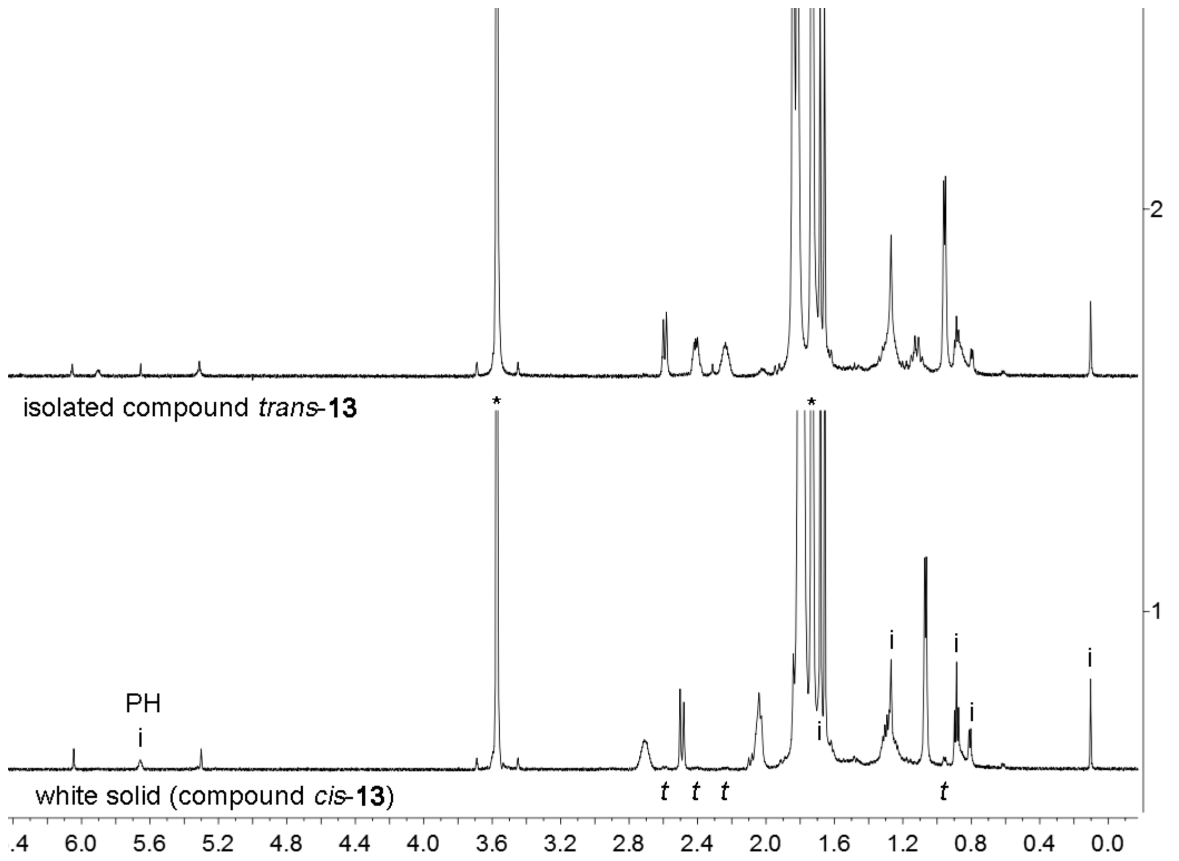

^{1}H NMR (600 MHz, 233 K, THF-d_8) spectra of (1) compound cis-**13** (admixed with trans-**13**; obtained white solid) and (2) isolated compound trans-**13** (see below) [i not identified components]*

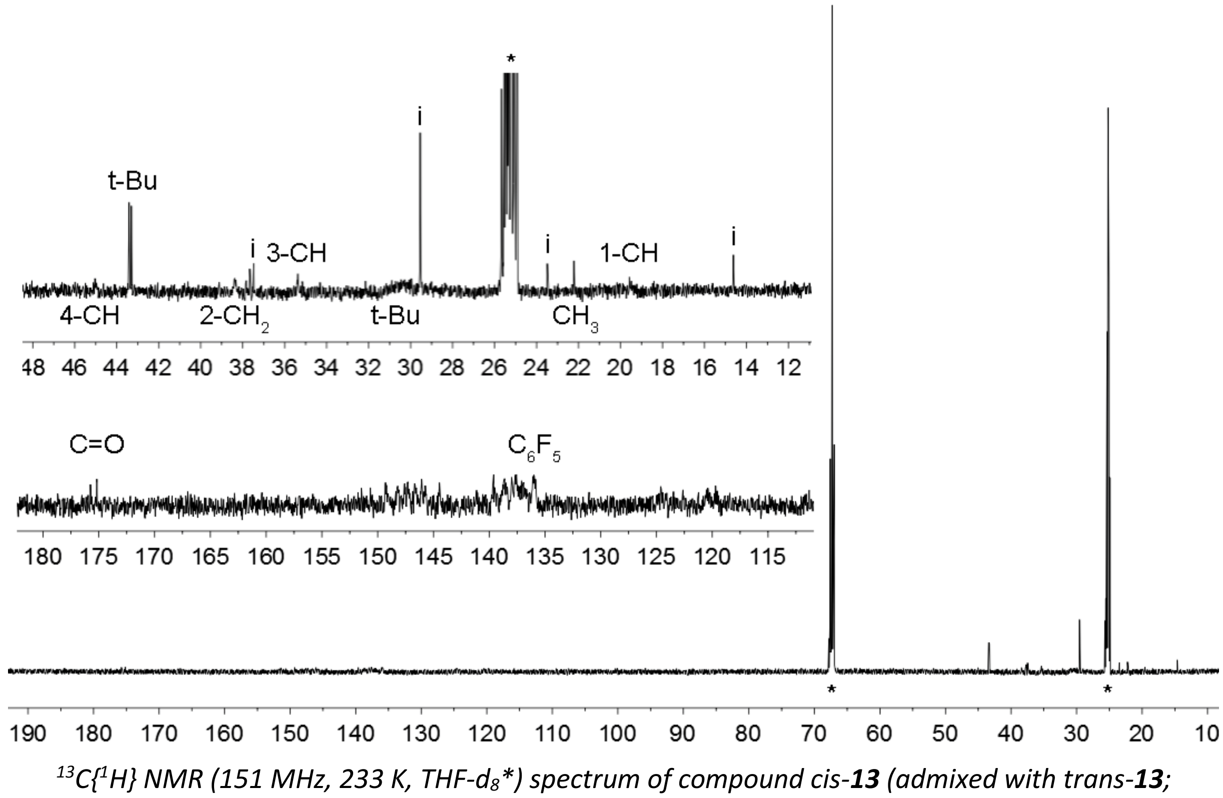

^{13}C{^{1}H} NMR (151 MHz, 233 K, THF-d_8) spectrum of compound cis-**13** (admixed with trans-**13**; obtained white solid) [i not identified components]*

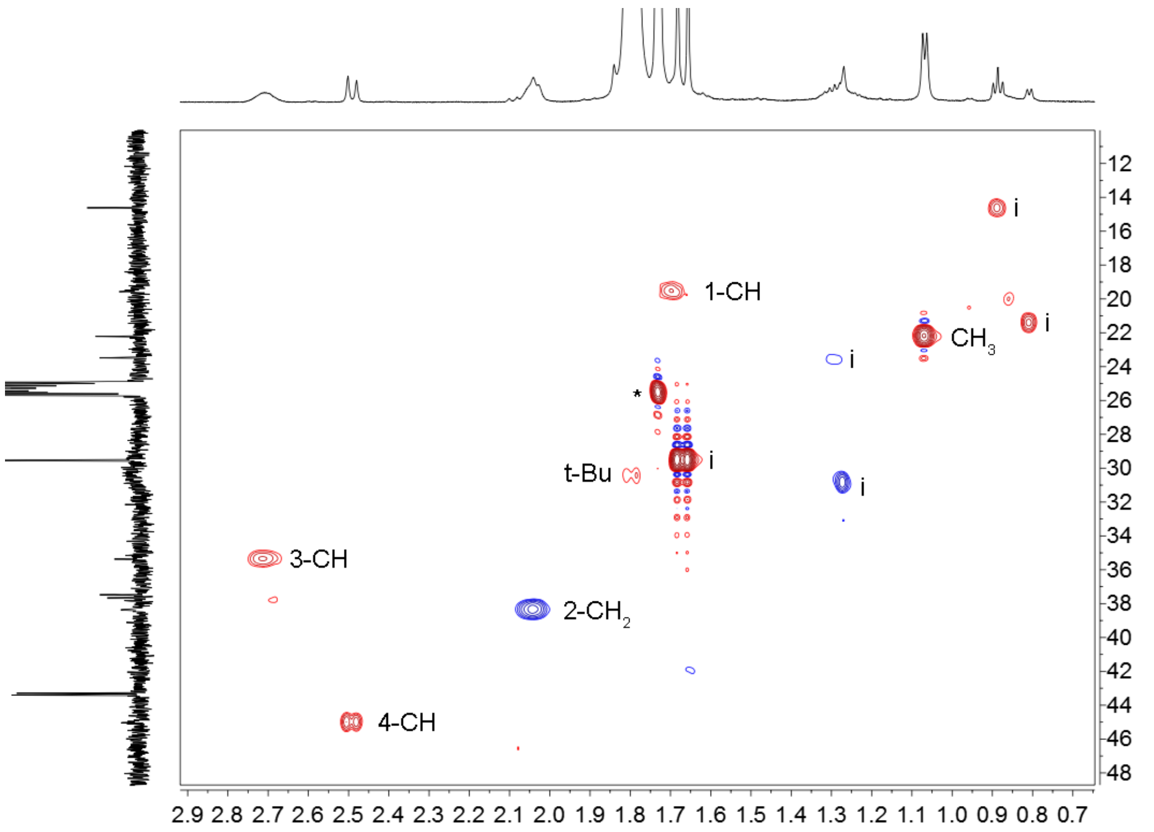

^{1}H, ^{13}C GHSQC (600 MHz / 151 MHz, 233 K, THF-d_8*) spectrum of compound cis-**13** (admixed with trans-13; obtained white solid) [i not identified components]

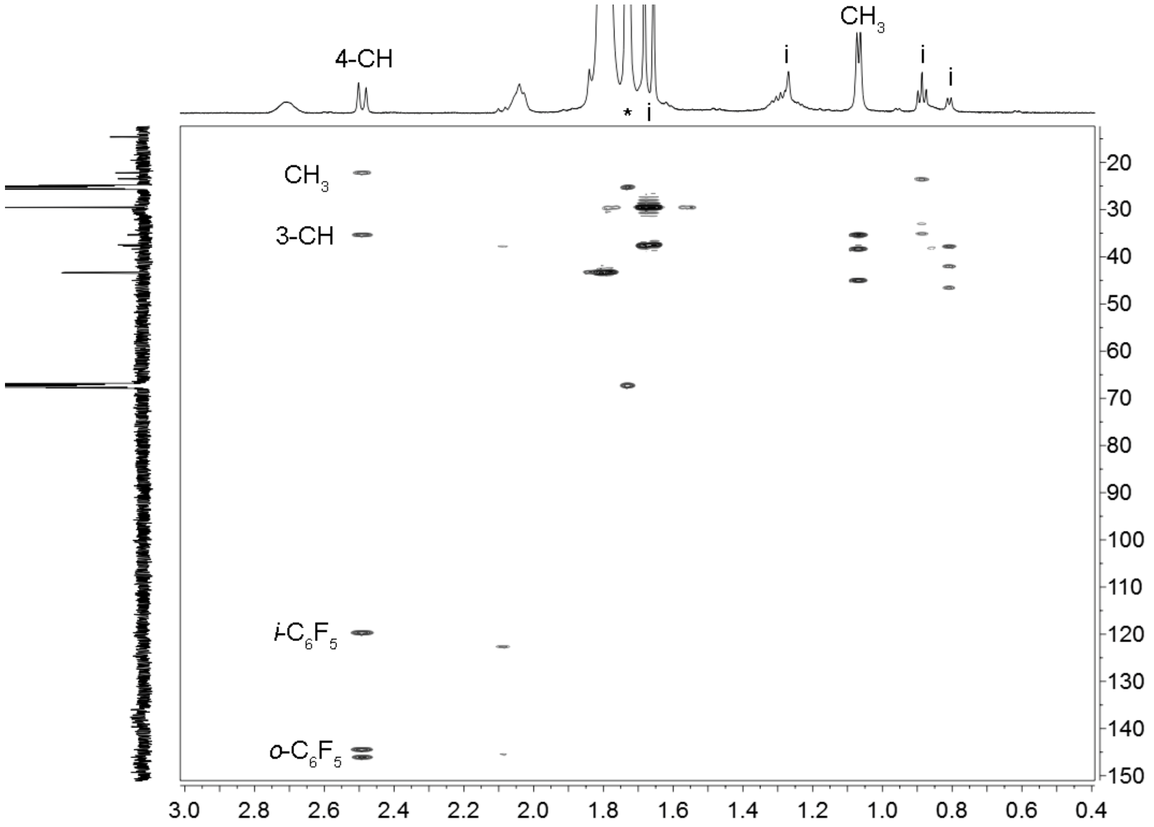

^{1}H, ^{13}C GHMBC (600 MHz / 151 MHz, 233 K, THF-d_8*) spectrum of compound cis-**13** (admixed with trans-13; obtained white solid) [i not identified components]

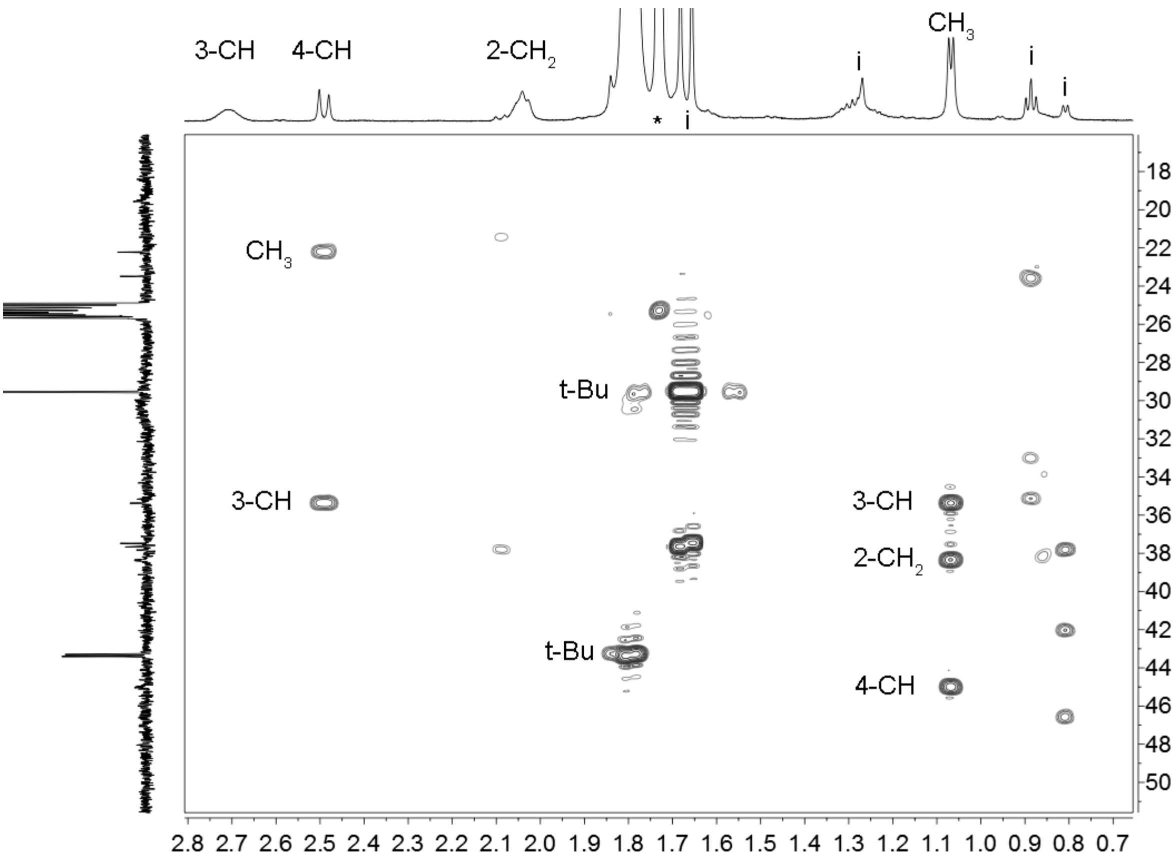

^{1}H, ^{13}C GHMBC (600 MHz / 151 MHz, 233 K, THF-d_8) spectrum of compound cis-**13** (admixed with trans-13; obtained white solid) [i not identified components]*

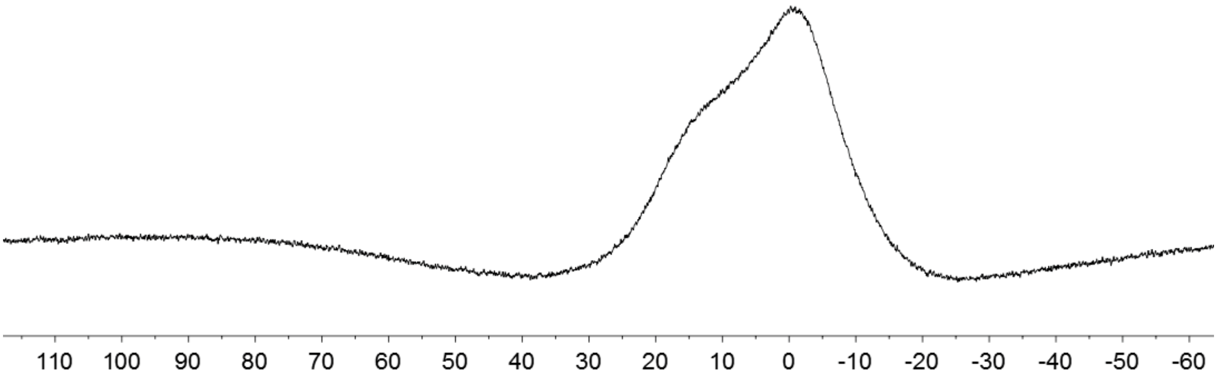

*^{11}B{^{1}H} NMR (192 MHz, 233 K, THF-d_8) spectrum of compound cis-**13** (admixed with trans-**13**; obtained white solid)*

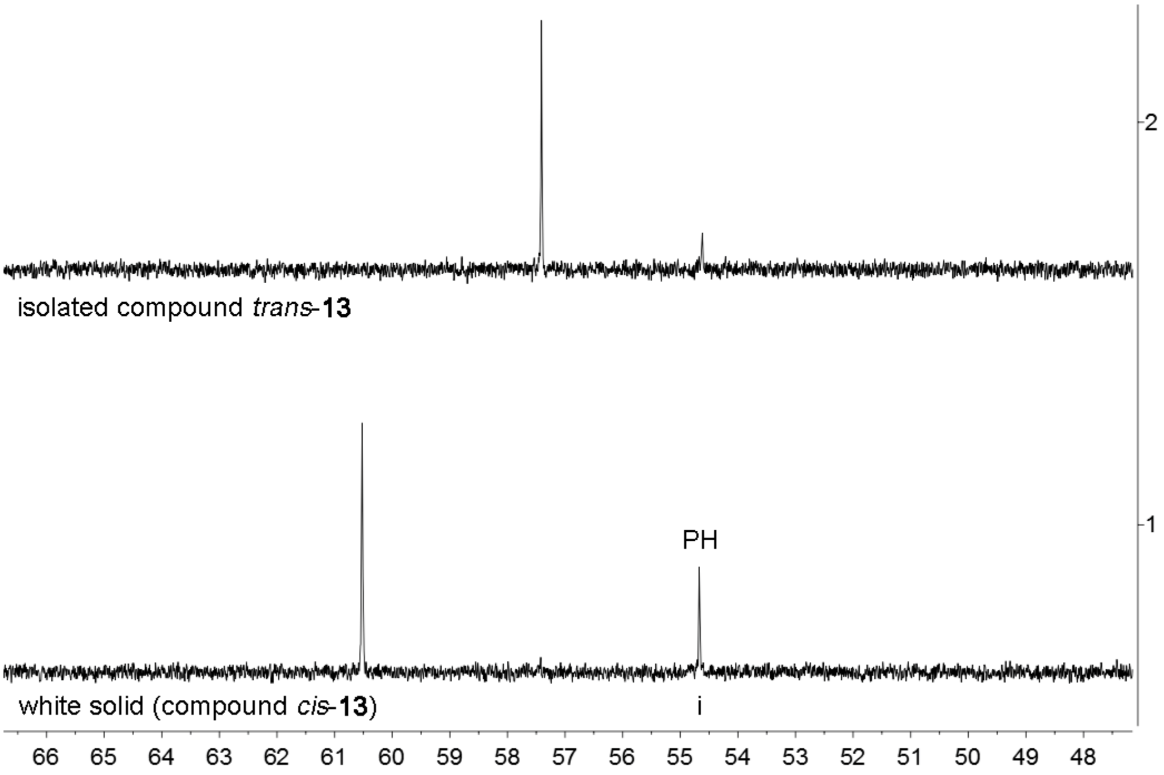

*^{31}P{^{1}H} NMR (243 MHz, 233 K, THF-d$_8$) spectra of (1) compound cis-**13** (admixed with trans-**13**; obtained white solid) and (2) isolated compound trans-**13** (see below) [i not identified component]*

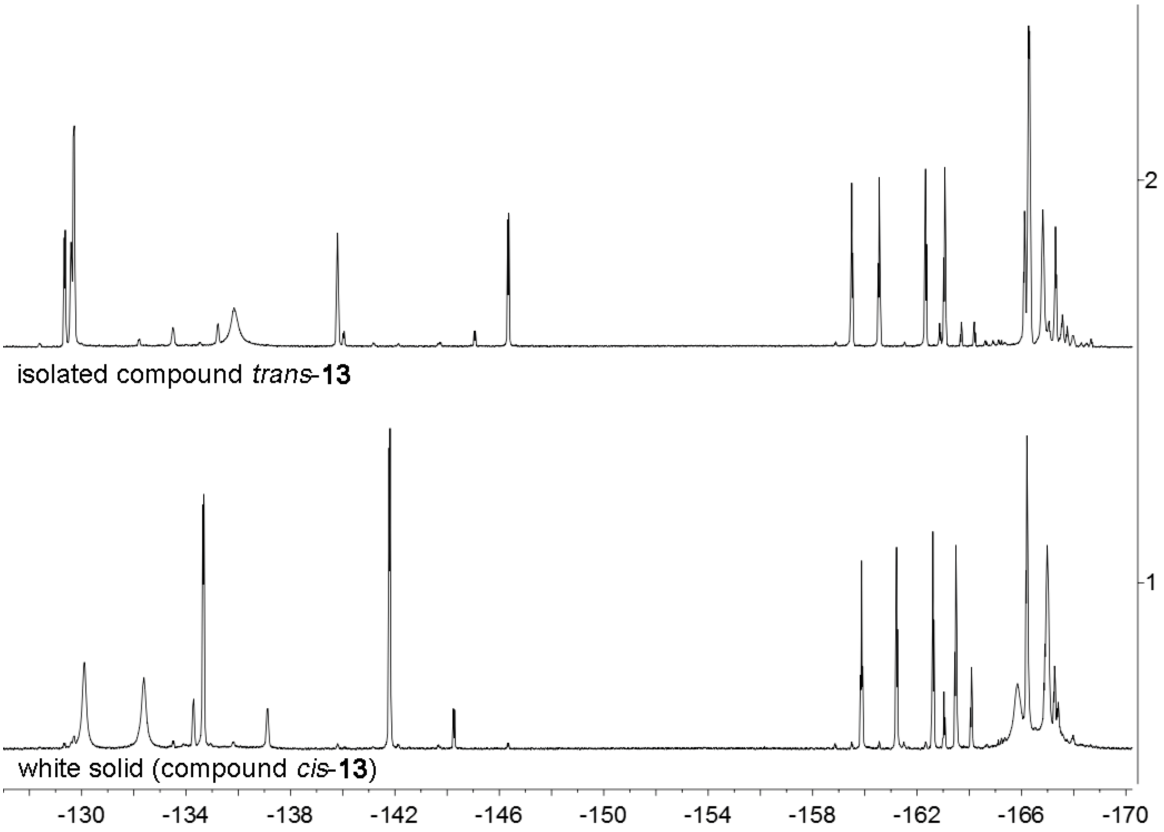

*^{19}F NMR (564 MHz, 233 K, THF-d$_8$) spectra of (1) compound cis-**13** (admixed with trans-**13**; obtained white solid) and (2) isolated compound trans-**13** (see below) [admixed with not identified components]*

Single crystals of compound *cis*-**13** suitable for the X-ray crystal structure analysis were obtained by slow diffusion of *n*-pentane into a solution of the obtained white solid in dichloromethane at -35 °C.

X-ray crystal structure analysis of compound *cis*-13: A colorless prism-like specimen of $C_{42}H_{35}B_2F_{20}O_2P \cdot 2 \times CH_2Cl_2$, approximate dimensions 0.139 mm x 0.205 mm x 0.217 mm, was used for the X-ray crystallographic analysis. The X-ray intensity data were measured. A total of 570 frames were collected. The total exposure time was 4.75 hours. The frames were integrated with the Bruker SAINT software package using a narrow-frame algorithm. The integration of the data using a monoclinic unit cell yielded a total of 107562 reflections to a maximum θ angle of 27.52° (0.77 Å resolution), of which 10980 were independent (average redundancy 9.796, completeness = 99.7%, R_{int} = 5.80%, R_{sig} = 2.63%) and 8994 (81.91%) were greater than $2\sigma(F^2)$. The final cell constants of a = 12.8388(5) Å, b = 20.0253(6) Å, c = 18.7529(6) Å, β = 97.3930(10)°, volume = 4781.3(3) Å^3, are based upon the refinement of the XYZ-centroids of 9879 reflections above 20 σ(I) with 4.620° < 2θ < 54.99°. Data were corrected for absorption effects using the multi-scan method (SADABS). The ratio of minimum to maximum apparent transmission was 0.964. The calculated minimum and maximum transmission coefficients (based on crystal size) are 0.9190 and 0.9470. The structure was solved and refined using the Bruker SHELXTL Software Package, using the space group $P2_1/n$, with Z = 4 for the formula unit, $C_{42}H_{35}B_2F_{20}O_2P \cdot 2 \times CH_2Cl_2$. The final anisotropic full-matrix least-squares refinement on F^2 with 696 variables converged at R1 = 4.22%, for the observed data and wR2 = 10.67% for all data. The goodness-of-fit was 1.053. The largest peak in the final difference electron density synthesis was 0.630 e$^-$/Å^3 and the largest hole was -1.262 e$^-$/Å^3 with an RMS deviation of 0.068 e$^-$/Å^3. On the basis of the final model, the calculated density was 1.631 g/cm^3 and F(000), 2368 e$^-$.

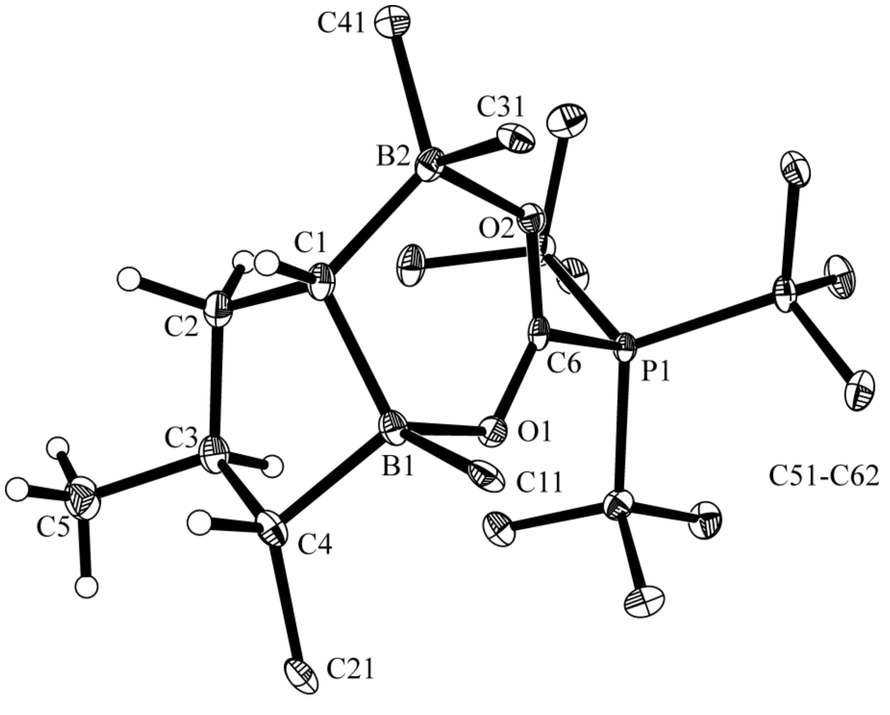

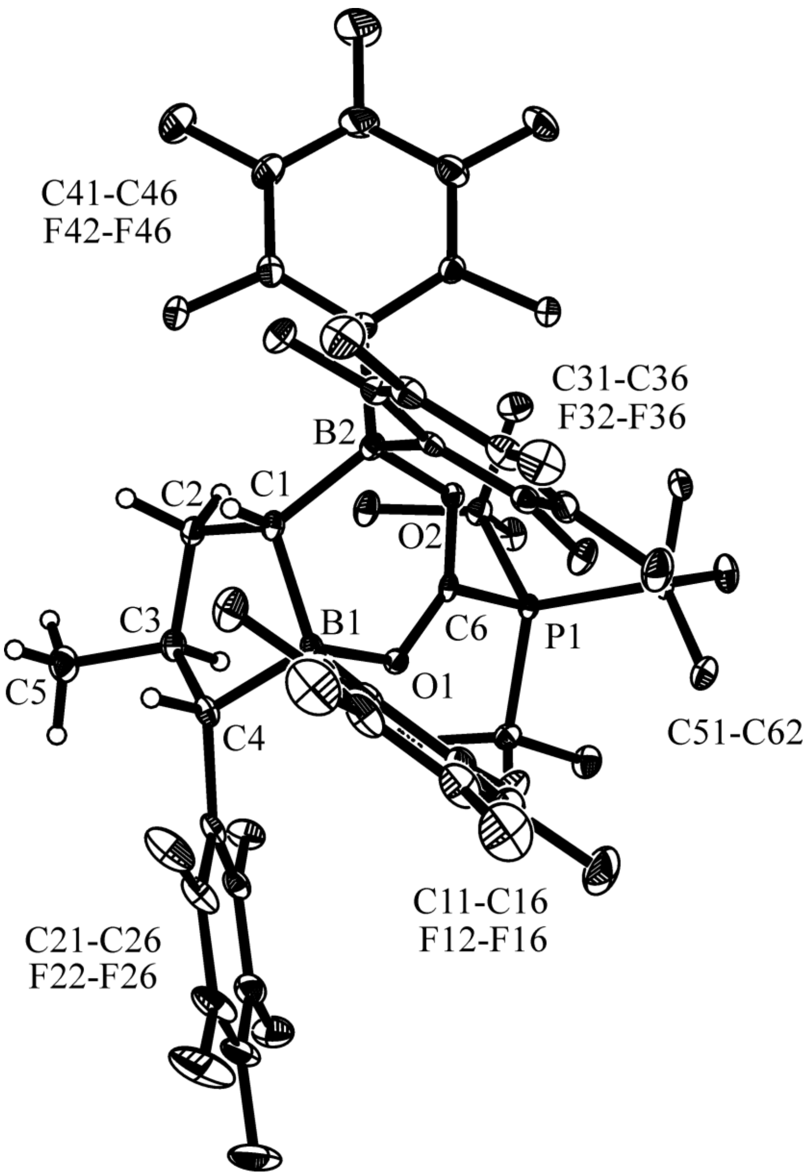
C41-C46
F42-F46
C31-C36
F32-F36
B2
C2
C1
O2
C3
B1
C6
P1
C5
O1
C4
C51-C62
C11-C16
F12-F16
C21-C26
F22-F26

Preparation of compound *trans*-**12**:

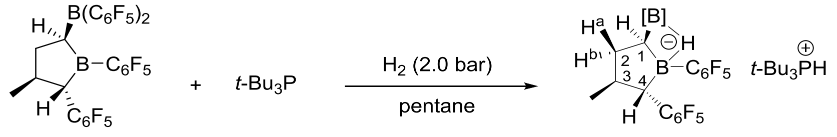

trans-**9** *trans*-**12** [B]: $B(C_6F_5)_2$

A solution of compound *trans*-**9** (75.8 mg, 0.10 mmol) and *tri-tert*-butylphosphane (20.2 mg, 0.10 mmol) in pentane (4.0 mL) was was exposed to a dihydrogen atmosphere (2.0 bar) at room temperature and stirred overnight. The formed white precipitate was collected by cannula filtration and washed with *n*-pentane (2 x 1 mL). Removal of all volatiles in vacuo gave compound *trans*-**12** (59.2 mg, 0.062 mmol, 62%) as a white solid.

Melting point: 173 °C.

Anal. Calc. for $C_{41}H_{37}B_2F_{20}P$: C, 51.17; H, 3.88. Found: C, 51.09; H, 3.67.

^{1}H NMR (500 MHz, 299 K, CD_2Cl_2): δ 5.10 (d, $^{1}J_{PH}$ = 428.2 Hz, 1H, PH), 2.77 (m, 1H, 3-CH), 2.29 (m, 1H, BH), 2.26 (m, 1H, 2-CH_2), 2.02 (m, 1H, 4-CH), 1.70 (d, $^{3}J_{PH}$ = 15.8 Hz, 27H, t-Bu), 1.67 (m, 1H, 1-CH), 0.67 (m, 1H, 2-CH_2), 0.55 (d, $^{3}J_{HH}$ = 6.4 Hz, 3H, CH_3).

^{13}C{^{1}H} NMR (126 MHz, 299 K, CD_2Cl_2): δ 49.0 (d, J = 4.6 Hz, 3-CH), 40.5 (2-CH_2), 38.1 (d, $^{1}J_{PC}$ = 26.7 Hz, t-Bu), 37.8 (br, 4-CH), 30.4 (t-Bu), 19.8 (CH_3), 14.2 (br, 1-CH), [C_6F_5 not listed].

^{1}H, ^{13}C GHSQC (500 MHz / 126 MHz, 299 K, CD_2Cl_2): δ^{1}H/ δ^{13}C 2.77 / 49.0 (3-CH), 2.26, 0.67 / 40.5 (2-CH_2), 2.02 / 37.8 (4-CH), 1.70 / 30.4 (t-Bu), 1.67 / 14.2 (1-CH), 0.55 / 19.8 (CH_3).

^{19}F NMR (470 MHz, 299 K, CD_2Cl_2): δ -130.4 (br, 1F), -131.6 (m, 2F), -132.3 (m, 2F), -133.0 (br m, 1F), -140.4 (m, 1F), -143.8 (m, 1F)(*o*-C_6F_5), -160.8 (t, $^{3}J_{FF}$ = 20.2 Hz), -161.3 (t, $^{3}J_{FF}$ = 20.2 Hz), -161.5 (t, $^{3}J_{FF}$ = 20.2 Hz), -164.5 (t, $^{3}J_{FF}$ = 20.9 Hz)(each 1F, *p*-C_6F_5), -165.6 (br m, 2F), -166.1 (br m, 1F), -166.3 (m, 3F), -166.9 (br m, 1F), -167.0 (m, 1F)(*m*-C_6F_5).

^{11}B{^{1}H} NMR (160 MHz, 299 K, CD_2Cl_2): δ -13.3 ($\nu_{1/2}$ ~ 260 Hz), -17.1 ($\nu_{1/2}$ ~ 200 Hz).

^{11}B NMR (160 MHz, 299 K, CD_2Cl_2): δ -13.3 ($\nu_{1/2}$ ~ 260 Hz), -17.1 ($\nu_{1/2}$ ~ 200 Hz).

^{31}P{^{1}H} NMR (202 MHz, 299 K, CD_2Cl_2): δ 60.7 ($\nu_{1/2}$ ~ 2 Hz).

^{31}P NMR (202 MHz, 299 K, CD_2Cl_2): δ 60.7 (dm, $^{1}J_{PH}$ ~ 428 Hz).

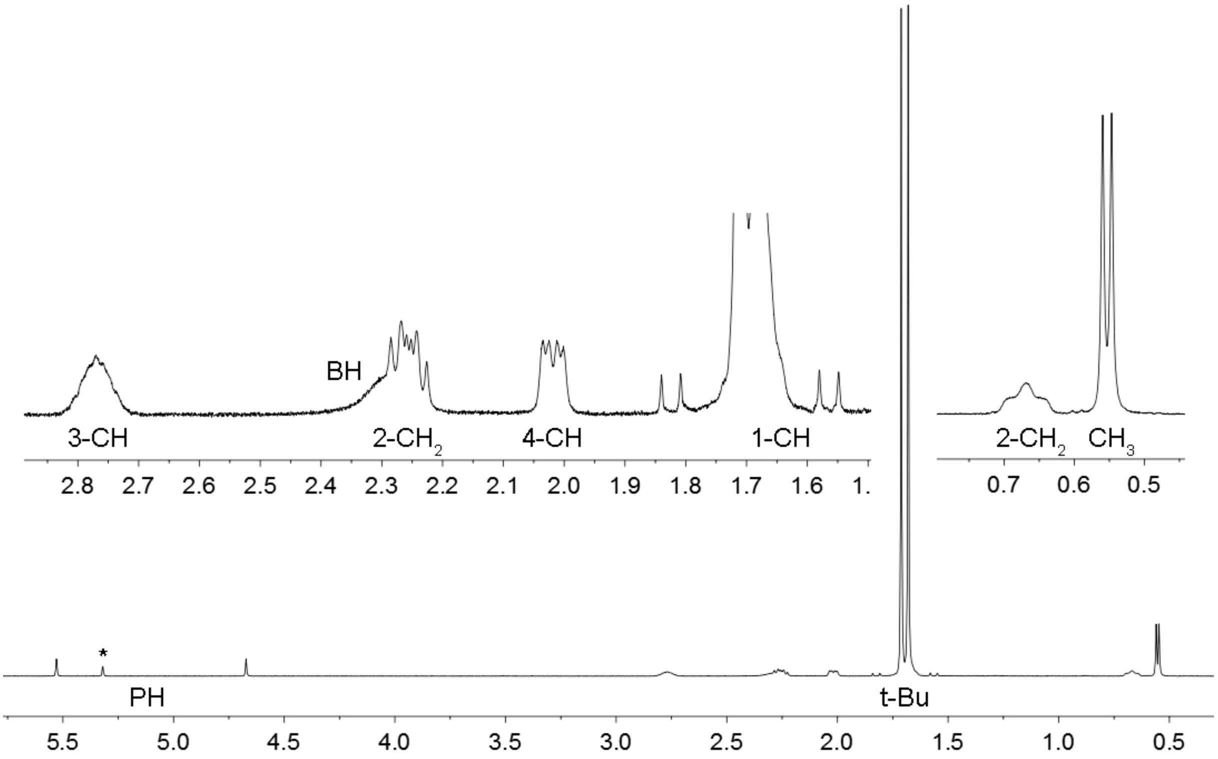

^{1}H NMR (500 MHz, 299 K, CD_2Cl_2**) spectrum of compound trans-***12***.*

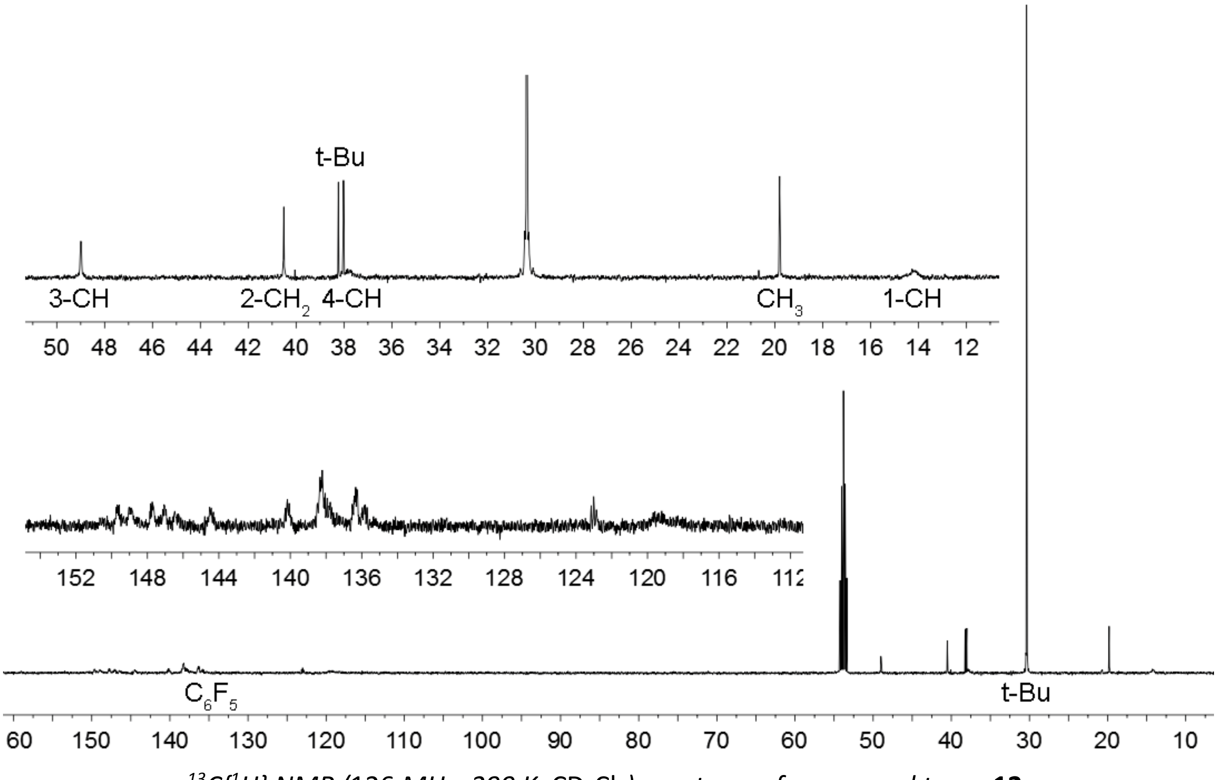

*^{13}C{^{1}H} NMR (*126 *MHz, 299 K,* CD_2Cl_2*) spectrum of compound trans-***12***.*

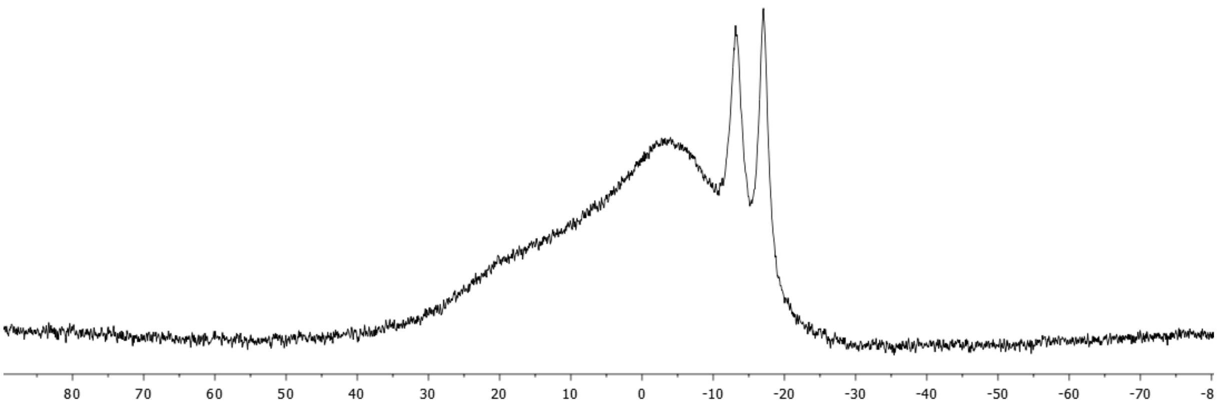

$^{11}B\{^{1}H\}$ NMR (160 MHz, 299 K, CD_2Cl_2*) spectrum of compound trans-***12***.*

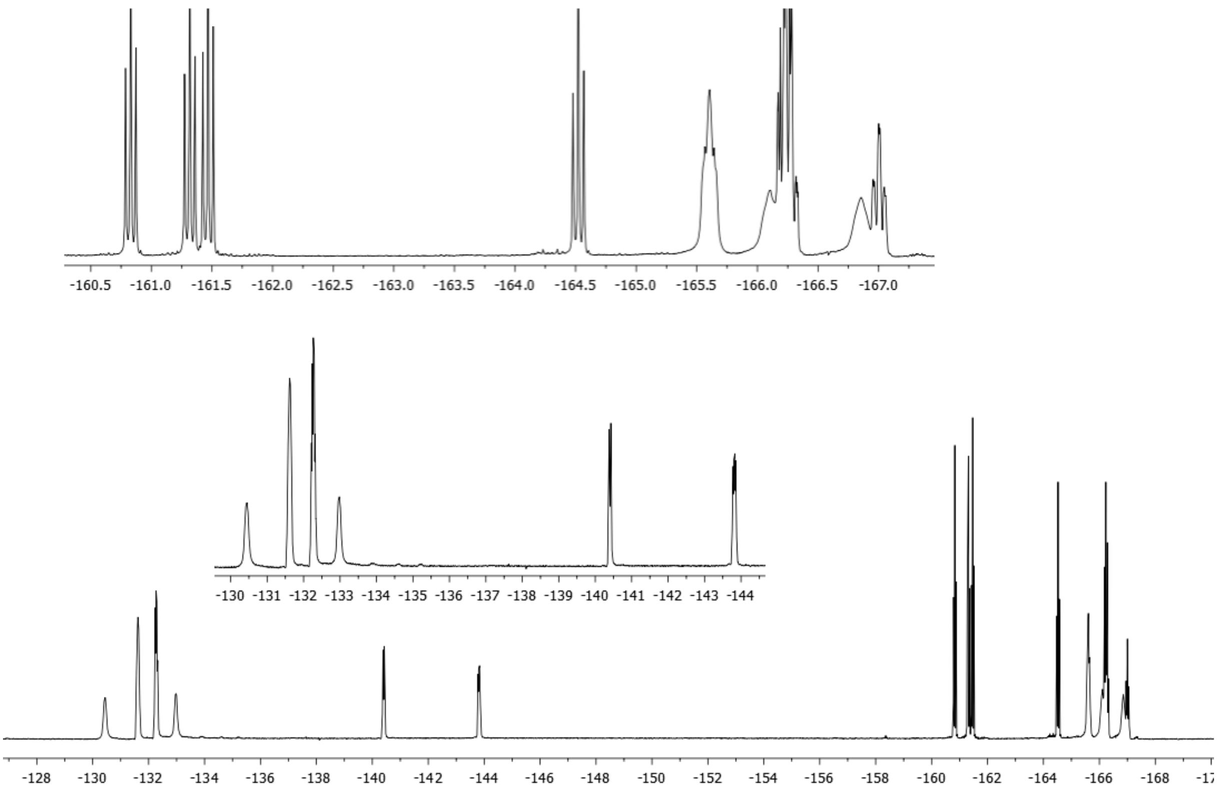

^{19}F NMR (470 MHz, 299 K, CD_2Cl_2*) spectrum of compound trans-***12***.*

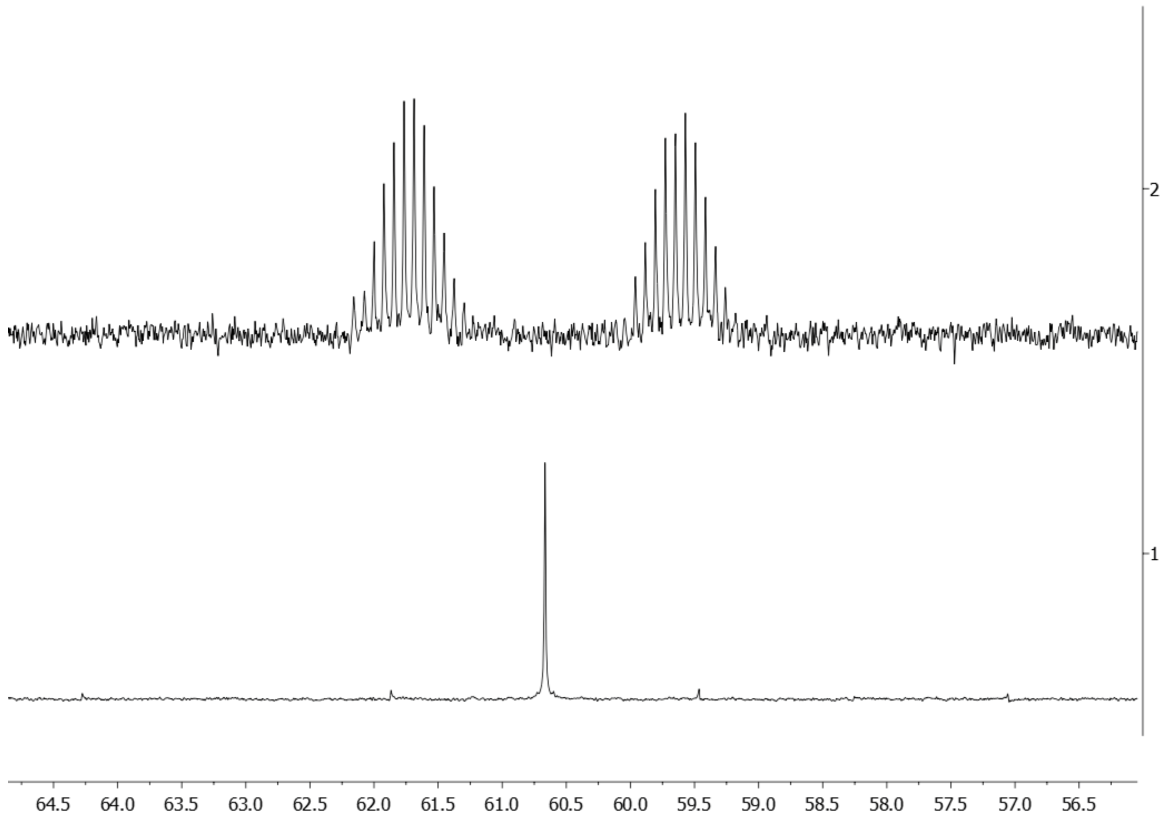

*(1) $^{31}P\{^{1}H\}$ and (2) ^{31}P NMR (*202 *MHz, 299 K,* CD_2Cl_2*) spectra of compound trans-***12***.*

Single crystals suitable for the X-ray crystal structure analysis were obtained by slow diffusion of pentane into a solution of compound *trans*-**12** in dichloromethane at room temperature.

X-ray crystal structure analysis of compound *trans*-12: A colorless plate-like specimen of $C_{41}H_{37}B_2F_{20}P$, approximate dimensions 0.058 mm x 0.205 mm x 0.268 mm, was used for the X-ray crystallographic analysis. The X-ray intensity data were measured. A total of 426 frames were collected. The total exposure time was 5.92 hours. The frames were integrated with the Bruker SAINT software package using a narrow-frame algorithm. The integration of the data using a monoclinic unit cell yielded a total of 8463 reflections to a maximum θ angle of 25.35° (0.83 Å resolution), of which 8463 were independent (average redundancy 1.000, completeness = 99.7%, R_{int} = 8.22%, R_{sig} = 5.36%) and 5979 (70.65%) were greater than 2σ(F^2). The final cell constants of a = 12.8848(7) Å, b = 21.5150(10) Å, c = 17.7497(8) Å, β = 109.7960(10)°, volume = 4629.7(4) Å^3, are based upon the refinement of the XYZ-centroids of 9906 reflections above 20 σ(I) with 5.062° < 2θ < 52.61°. Data were corrected for absorption effects using the multi-scan method (SADABS). The ratio of minimum to maximum apparent transmission was 0.922. The calculated minimum and maximum transmission coefficients (based on crystal size) are 0.9560 and 0.9900. The structure was solved and refined using the Bruker SHELXTL Software Package, using the space group $P2_1/n$, with Z = 4 for the formula unit,

$C_{41}H_{37}B_2F_{20}P$. The final anisotropic full-matrix least-squares refinement on F^2 with 716 variables converged at R1 = 5.89%, for the observed data and wR2 = 15.50% for all data. The goodness-of-fit was 1.047. The largest peak in the final difference electron density synthesis was 0.519 $e^-/Å^3$ and the largest hole was -0.289 $e^-/Å^3$ with an RMS deviation of 0.073 $e^-/Å^3$. On the basis of the final model, the calculated density was 1.381 g/cm^3 and F(000), 1952 e^-.

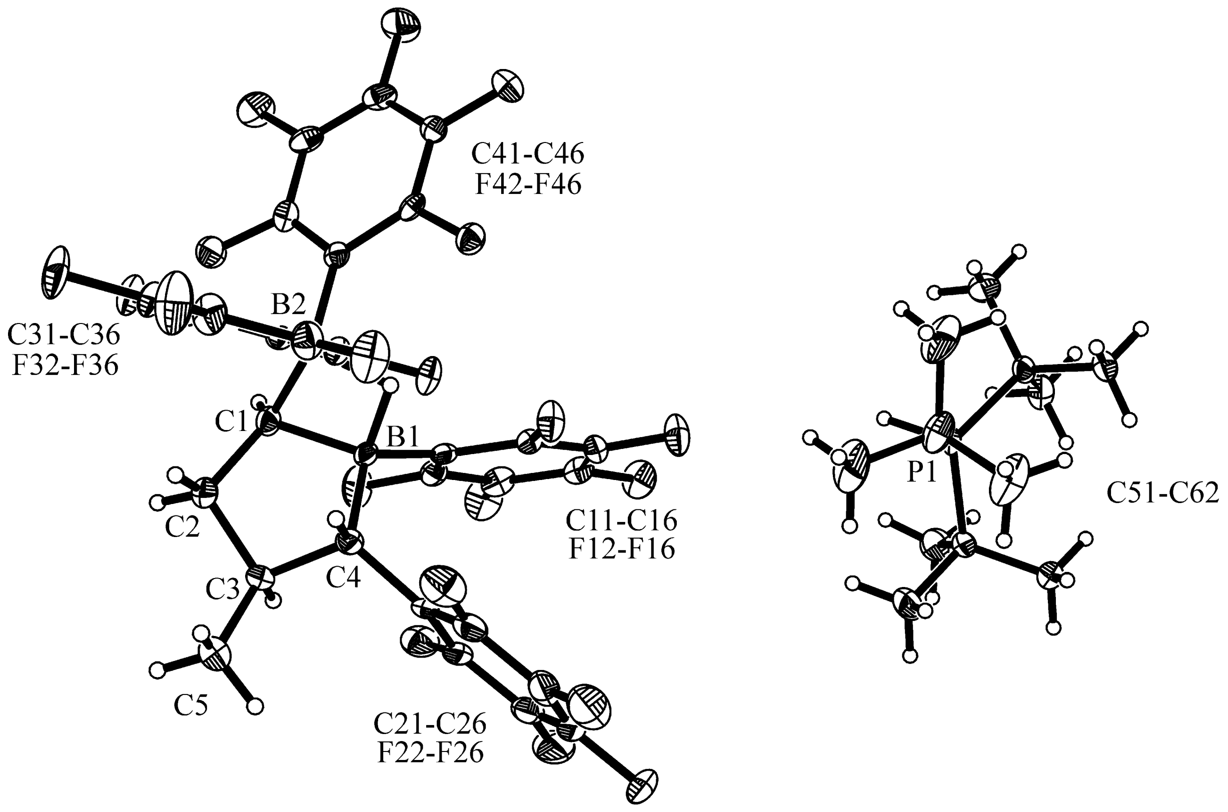

Preparation of compound *trans*-**13**:

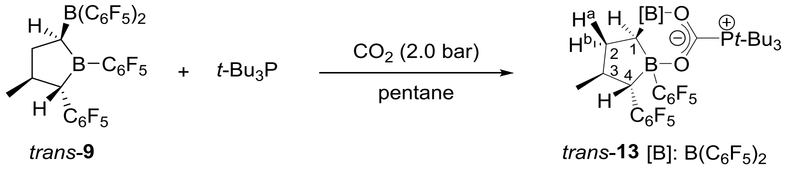

1st Experiment: A solution of compound *trans*-**9** (75.8 mg, 0.10 mmol) and *tri-tert*-butylphosphane (20.2 mg, 0.10 mmol) in pentane (5.0 mL) was exposed to CO_2 (2.0 bar) at room temperature and then stirred overnight. The formed white precipitate was collected by cannula filtration and washed with pentane (3 x 1 mL). After removal of all volatiles in vacuo, compound *trans*-**13** (76.2 mg, 0.076 mmol, 76%) was obtained as a white solid.

Melting point: 124 °C.

IR (KBr, selective wavenumber): $\tilde{\nu}$ [cm^{-}1] = 1598 (s, C-O asym. stretch).

Anal. Calc. for $C_{42}H_{35}B_2F_{20}O_2P$: C, 50.23; H, 3.51. Found: C, 49.96; H, 3.27.

The solubility of the white solid is poor in benzene-d_6, toluene-d_8, CD_2Cl_2, $CDCl_3$, but a solution of the white solid in THF-d_8 showed a mixture of compounds. The major component was assigned as compound *trans*-**13**.

^{1}H NMR (600 MHz, 233 K, THF-d_8): δ 2.59 (m, 1H, 4-CH), 2.41, 1.12 (each m, each 1H, 2-CH_2), 2.24 (m, 1H, 3-CH), 1.83 (d, $^3J_{PH}$ = 15.3 Hz, 27H, t-Bu), 1.72 (m, 1H, 1-CH), 0.96 (d, $^3J_{HH}$ = 6.4 Hz, 3H, CH_3).

^{13}C{^{1}H} NMR (151 MHz, 233 K, THF-d_8): δ 173.4 (d, $^1J_{PC}$ = 90.0 Hz, C=O), 44.8 (3-CH), 43.4 (d, $^1J_{PC}$ =16.9 Hz, t-Bu), 43.1 (4-CH), 41.5 (br, 2-CH_2), 30.6 (br, t-Bu), 24.7 (br, 1-CH), 20.6 (CH_3), [C_6F_5 not listed].

^{1}H, ^{13}C GHSQC (600 MHz / 150 MHz, 233 K, THF-d_8): ^{1}H/ δ^{13}C 2.59 / 43.1 (4-CH), 2.41, 1.12 / 41.5 (2-CH_2), 2.24 / 44.8 (3-CH), 1.83 / 30.6 (t-Bu), 1.72 / 24.7 (1-CH), 0.96 / 20.6 (CH_3).

^{19}F NMR (564 MHz, 233 K, THF-d_8): δ (major 91 mol%) -129.4 (1F), -129.6 (1F), -129.7 (2F), -135.9 (2F), -139.8 (1F), -146.3 (1F)(each br m, *o*), -159.6 (t, $^3J_{FF}$ = 20.8 Hz), -160.6 (t, $^3J_{FF}$ = 20.6 Hz), -162.3 (t, $^3J_{FF}$ = 20.8 Hz), -163.1 (t, $^3J_{FF}$ = 21.5 Hz)(each 1F, *p*), -166.1 (1F), -166.3 (4F), -166.8 (2F), -167.3 (1F)(each br m, *m*)(C_6F_5).

^{11}B{^{1}H} NMR (192 MHz, 233 K, THF-d_8): δ n.o. (overlap with the "glass hill").

^{31}P{^{1}H} NMR (243 MHz, 233 K, THF-d_8): δ 57.4 ($\nu_{1/2}$ ~ 3 Hz, 89 mol%)[admixed with δ: 54.6 ($\nu_{1/2}$ ~ 6 Hz, 11 mol%)]

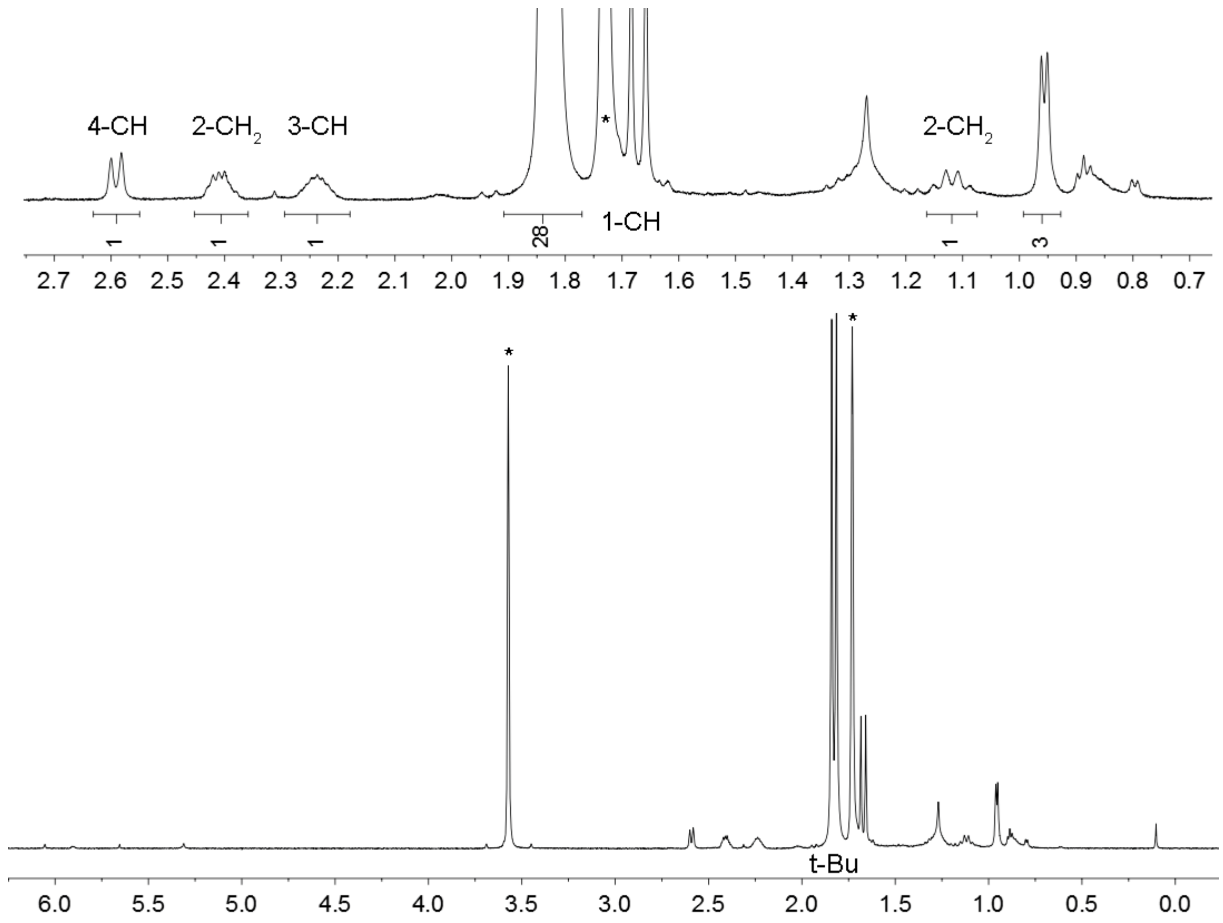

^{1}H NMR (600 MHz, 233 K, THF-d_8) spectrum of compound trans-***13***.

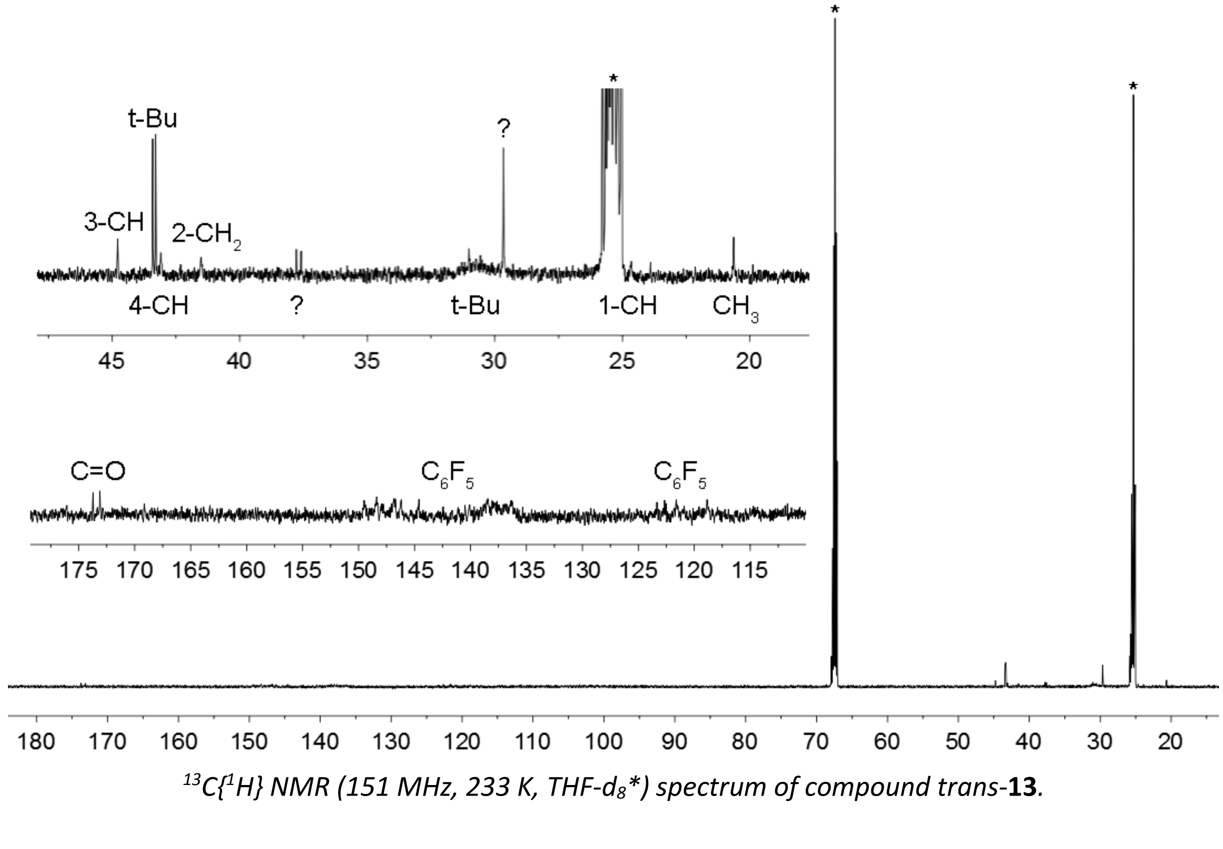

^{13}C{^{1}H} NMR (151 MHz, 233 K, THF-d_8) spectrum of compound trans-***13***.

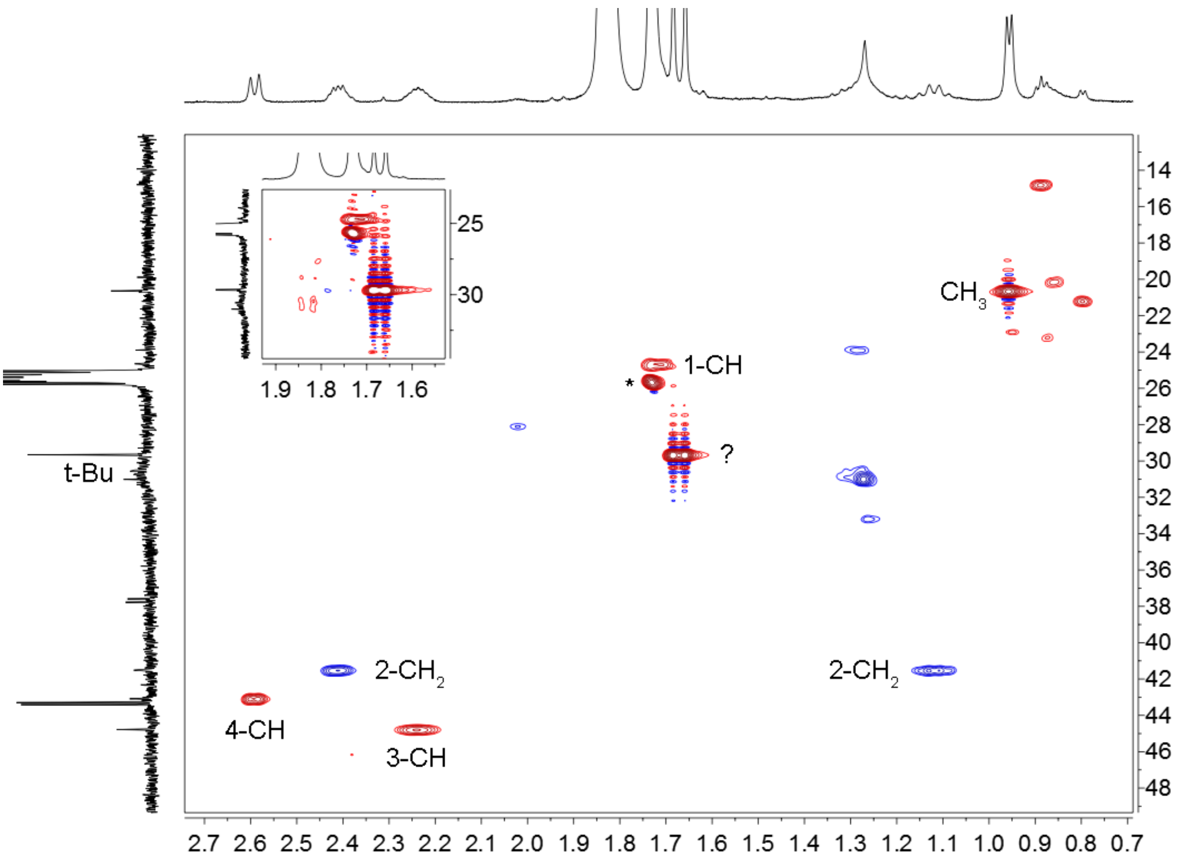

^{1}H, ^{13}C GHSQC (600 MHz / 151 MHz, 233 K, THF-d_8*) spectrum of compound trans-**13**.

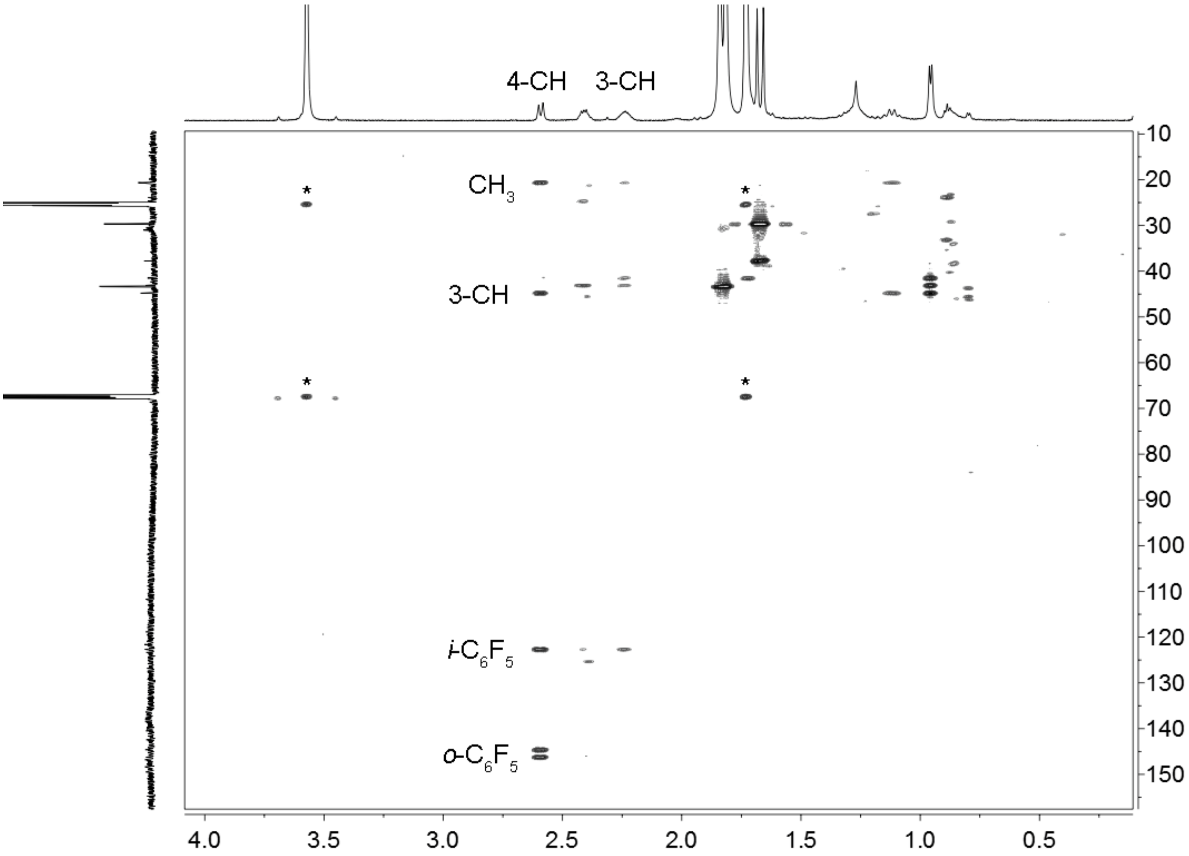

^{1}H, ^{13}C GHMBC (600 MHz / 151 MHz, 233 K, THF-d_8*) spectrum of compound trans-**13**.

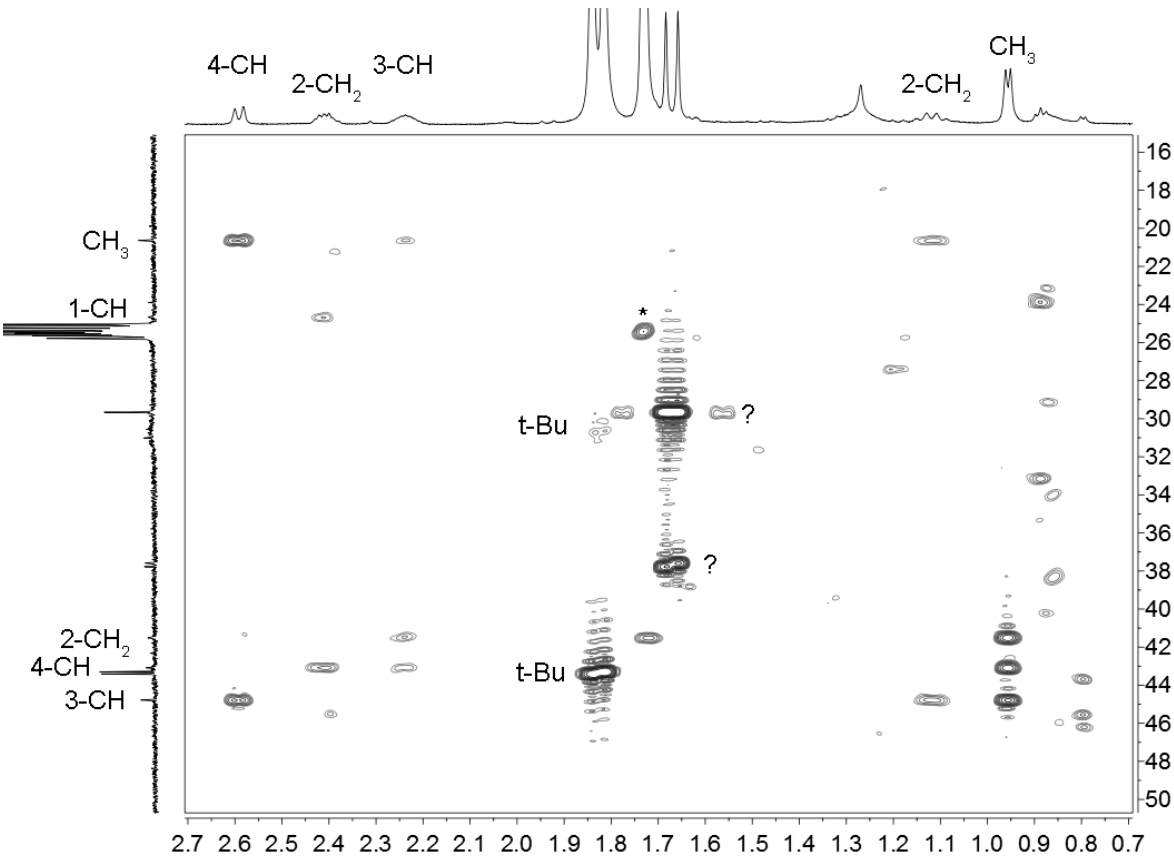

*^{1}H, ^{13}C GHMBC (600 MHz / 151 MHz, 233 K, THF-d_8) spectrum of compound trans-**13**.*

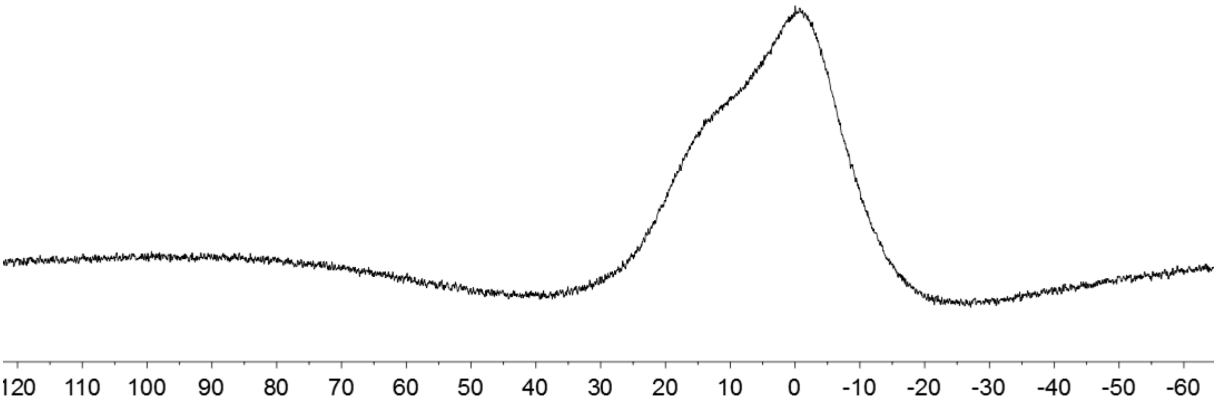

*$^{11}B\{^{1}H\}$ NMR (192 MHz, 233 K, THF-d_8) spectrum of compound trans-**13**.*

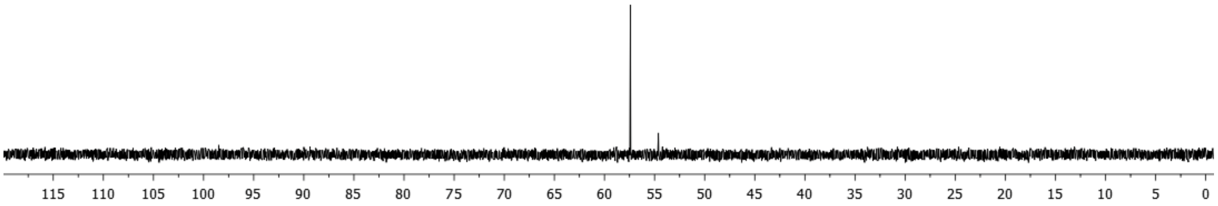

*$^{31}P\{^{1}H\}$ NMR (243 MHz, 233 K, THF-d_8) spectrum of compound trans-**13**.*

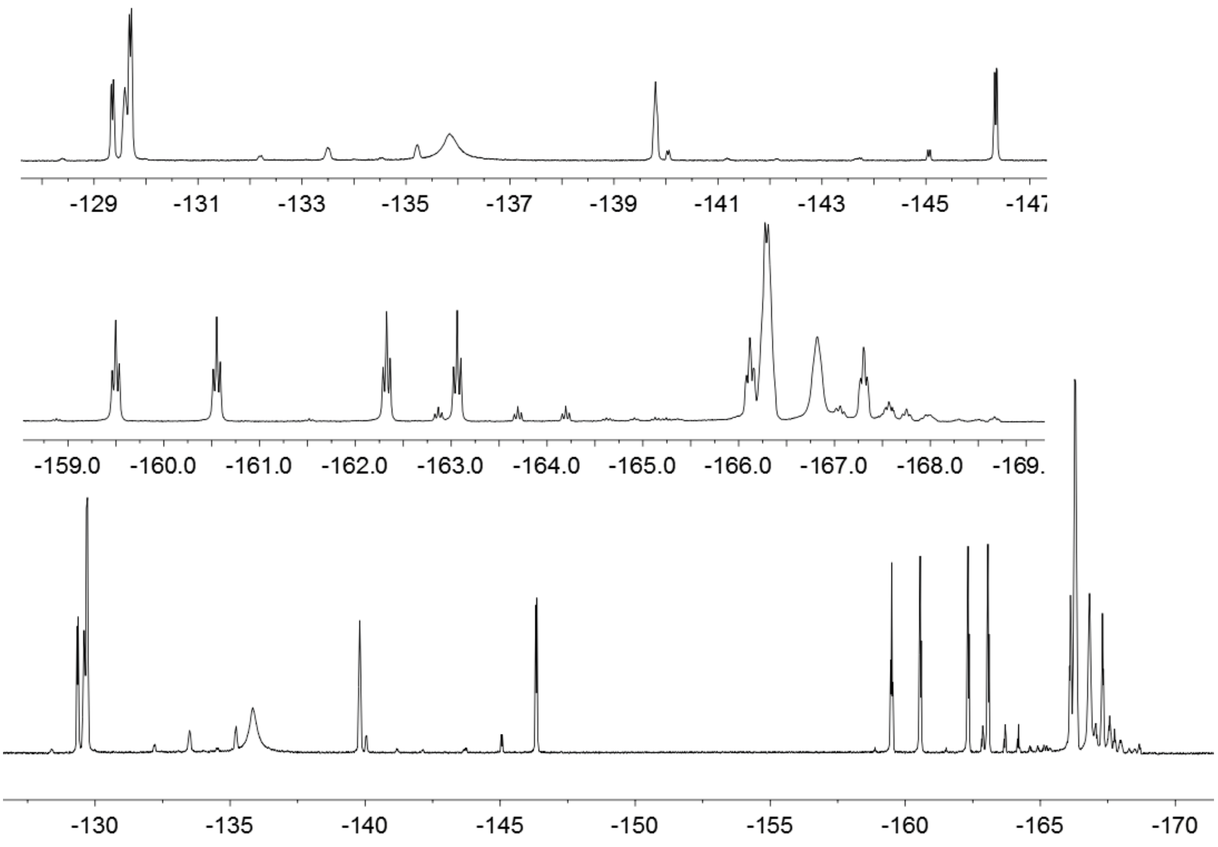

*^{19}F NMR (564 MHz, 233 K, THF-d_8) spectrum of compound trans-***13**.

2nd Experiment: Single crystals of compound *trans*-**13** suitable for the X-ray crystal structure analysis were obtained directly from a reaction solution of compound *trans*-**9** (37.9 mg) and *tri-tert*-butylphosphane (10.1 mg) and dichloromethane (1.0 mL) in a CO_2 atmosphere (2.0 bar) at room temperature.

X-ray crystal structure analysis of compound *trans*-13: formula $C_{42}H_{35}B_2F_{20}O_2P$, M = 1004.29, colourless crystal, 0.05 x 0.03 x 0.01 mm, a = 10.0179(3), b = 11.2774(5), c = 19.6359(11) Å, α = 89.016(3), β = 87.552(3), γ = 73.104(2)°, V = 2120.7(2) Å^3, ρ_{calc} = 1.573 gcm^{-3}, μ = 0.191 mm^{-1}, empirical absorption correction (0.990 ≤ T ≤ 0.998), Z = 2, triclinic, space group $P\bar{1}$ (No. 2), λ = 0.71073 Å, T = 223(2) K, ω and ϕ scans, 14903 reflections collected (±h, ±k, ±l), 7051 independent (R_{int} = 0.111) and 3663 observed reflections [I>2σ(I)], 616 refined parameters, R = 0.134, wR^2 = 0.274, max. (min.) residual electron density 0.42 (-0.41) e.Å^{-3}, hydrogen atoms were calculated and refined as riding atoms.

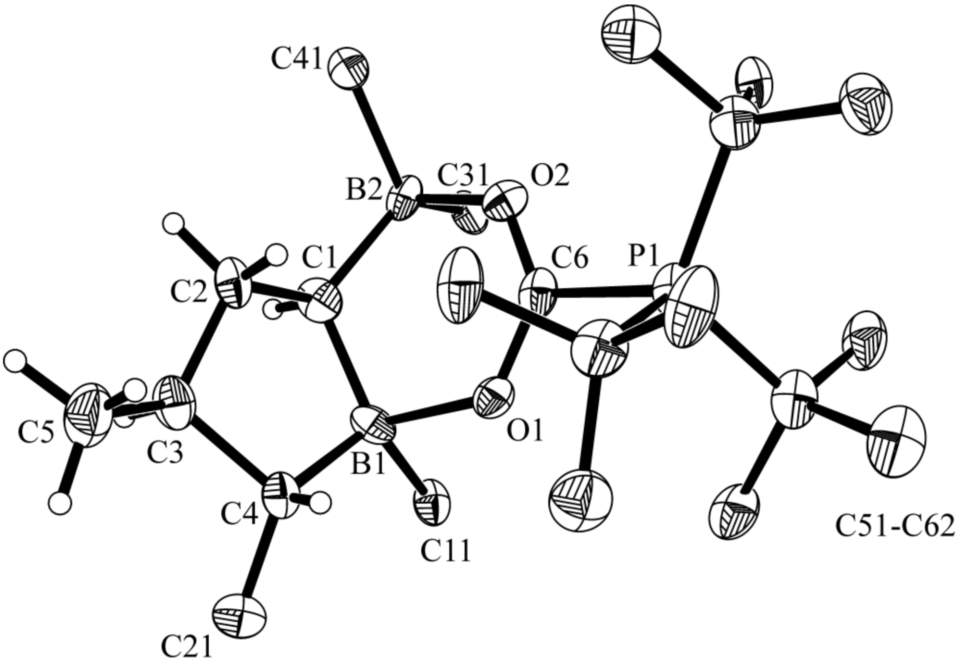
C41
B2
C31
O2
C1
C6
P1
C2
C5
C3
B1
O1
C4
C11
C51-C62
C21

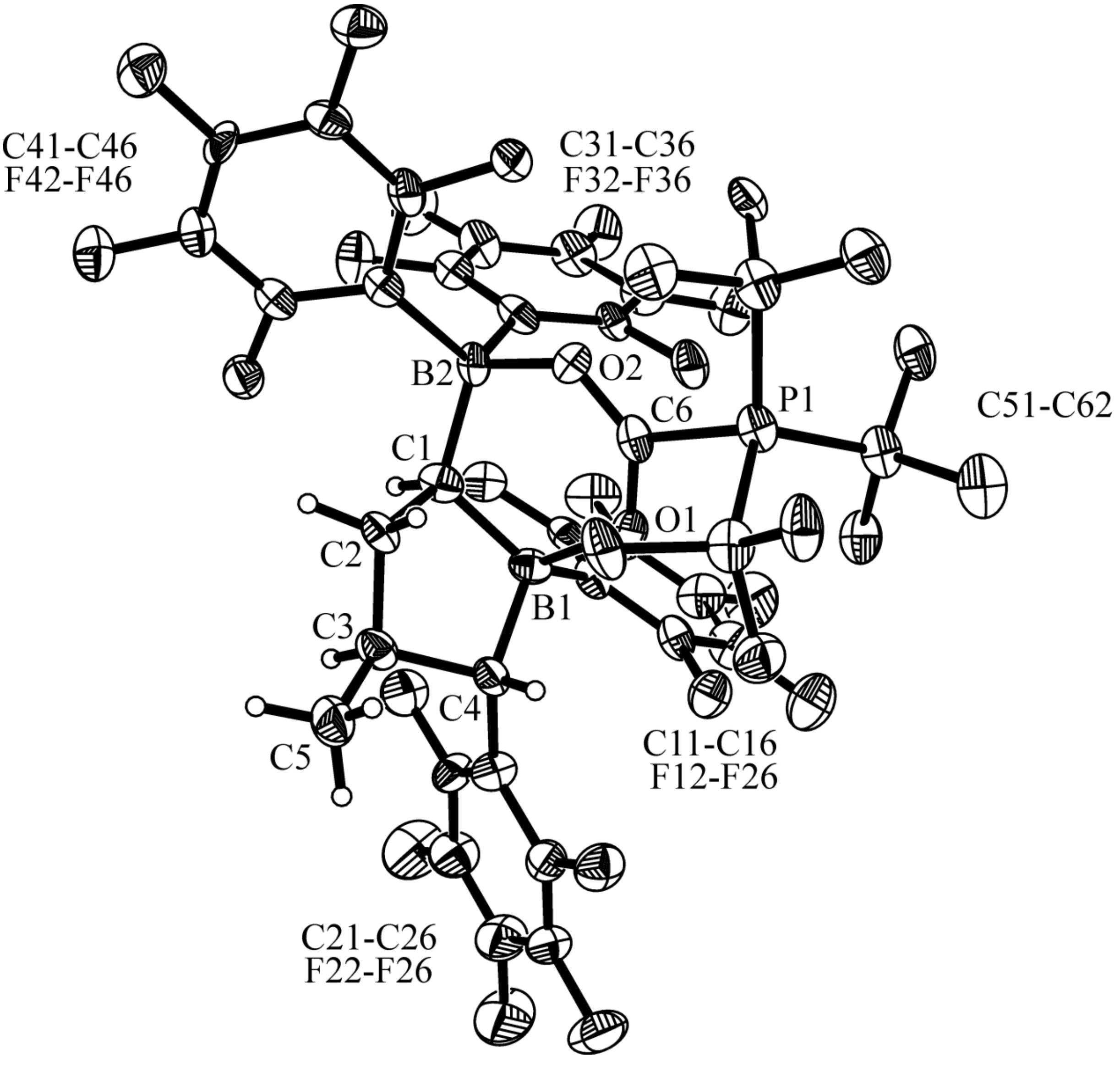
C41-C46
F42-F46
C31-C36
F32-F36
B2
O2
P1
C51-C62
C6
C1
O1
C2
B1
C3
C4
C5
C11-C16
F12-F26
C21-C26
F22-F26

Control experiments:

1st Experiment: *Hydroboration of cyclopropyl acetylene (**5b**) with $HB(C_6F_5)_2$ (in situ, NMR scale)*

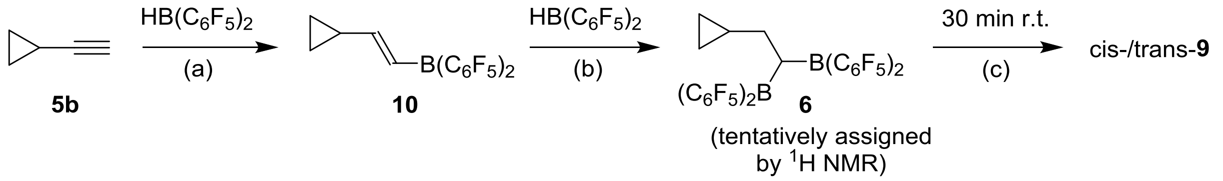

a) Bis(pentafluorophenyl)borane (34.5 mg, 0.10 mmol), cyclopropyl acetylene (**5b**) (6.6 mg, 0.10 mmol) and C_6D_6 (0.8 mL) were successively added to a J Young NMR tube to give a suspension. After shaking for 1 minute at room temperature, the obtained clear yellow solution was characterized by NMR experiments immediately.

[The NMR data of compound **10** were consistent to those reported in the literature: F. Türkyilmaz, G. Kehr, J. Li, C. G. Daniliuc, M. Tesch, A. Studer and G. Erker, *Angew. Chem., Int. Ed.*, 2016, **55**, 1470.]

Compound 10:

^{1}H NMR (600 MHz, 299 K, C_6D_6): δ: 6.91 (d, $^{3}J_{HH}$ = 16.9 Hz, 1H, BCH), 6.35 (dd, $^{3}J_{HH}$ = 16.9 Hz, $^{3}J_{HH}$ = 9.8 Hz, 1H, =CH), 1.32 (m, 1H, CH), 0.60, 0.38 (each m, each 2H, CH_2).

^{13}C{^{1}H} NMR (151 MHz, 299 K, C_6D_6): δ: 178.0 (=CH), 147.5 (dm, $^{1}J_{FC}$ = 253 Hz, C_6F_5), 142.9 (dm, $^{1}J_{FC}$ = 248 Hz, C_6F_5), 137.5 (dm, $^{1}J_{FC}$ = 255 Hz, C_6F_5), 133.0 (br, BCH), 114.1 (br, *i*-C_6F_5), 19.9 (CH), 11.7 (CH_2).

^{1}H, ^{13}C GHSQC (600 MHz / 150 MHz, 299 K, C_6D_6): δ^{1}H/δ^{13}C 6.91 / 133.0 (BCH), 6.35 / 178.0 (=CH), 1.32 / 19.9 (CH), 0.60, 0.38 / 11.7 (CH_2).

^{19}F NMR (564 MHz, 299 K, C_6D_6): -130.3 (m, 2F, *o*-C_6F_5), -149.2 (t, $^{3}J_{FF}$ = 20.8 Hz, 1F, *p*-C_6F_5), -161.5 (m, 2F, *m*-C_6F_5), [Δδ $^{19}F_{m,p}$ = 12.3].

^{11}B{^{1}H} NMR (192 MHz, 299 K, C_6D_6): δ: 56.9 ($\nu_{1/2}$ ~ 650 Hz).

^{11}B NMR (192 MHz, 299 K, C_6D_6): δ: 56.9 ($\nu_{1/2}$ ~ 650 Hz).

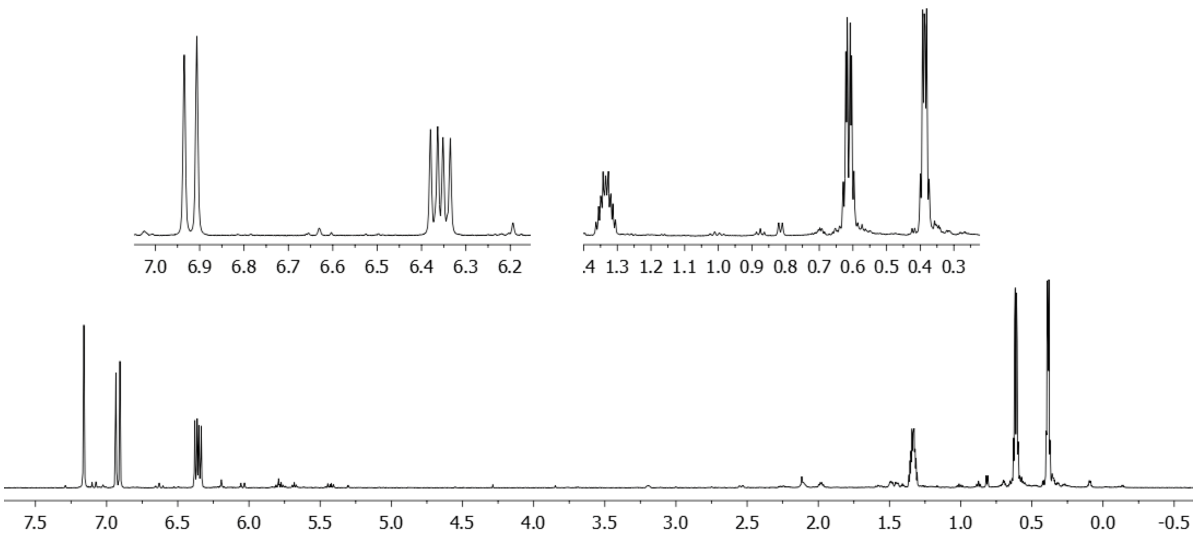

^{1}H NMR (600 MHz, 299 K, C_6D_6) spectrum of compound **10**.

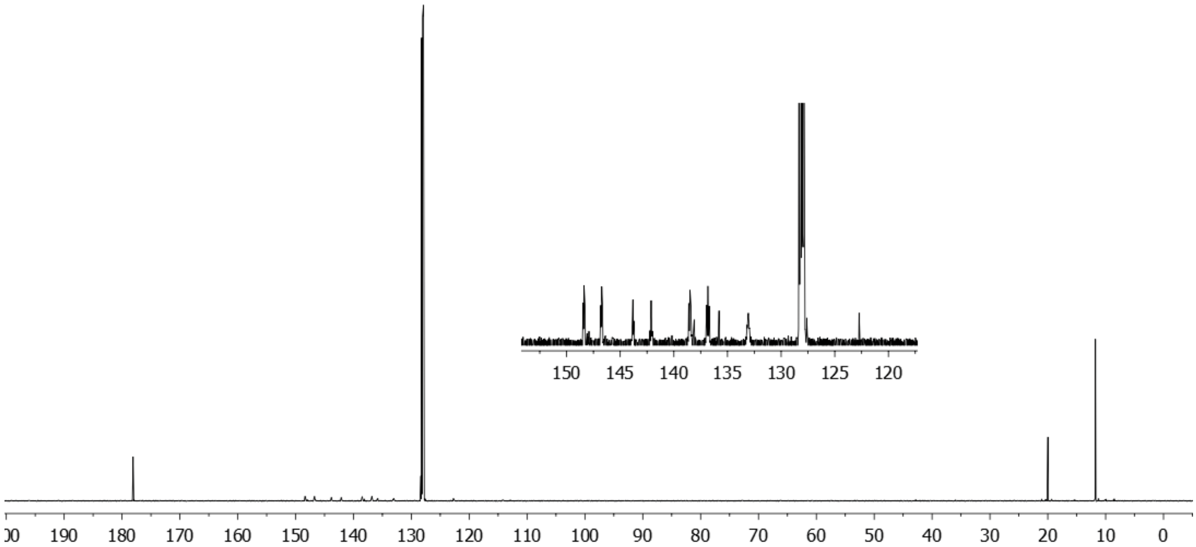

$^{13}C\{^{1}H\}$ NMR (150 MHz, 299 K, C_6D_6) spectrum of compound **10**.

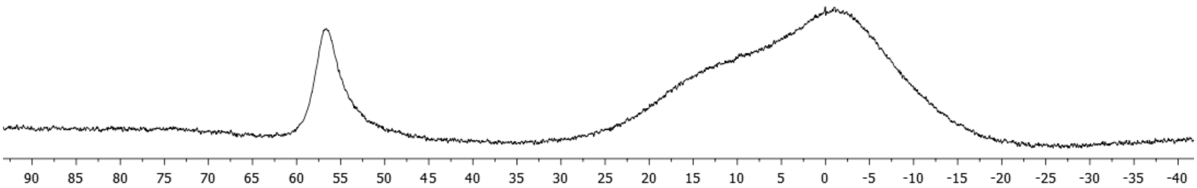

$^{11}B\{^{1}H\}$ NMR (192 MHz, 299 K, C_6D_6) spectrum of compound **10**.

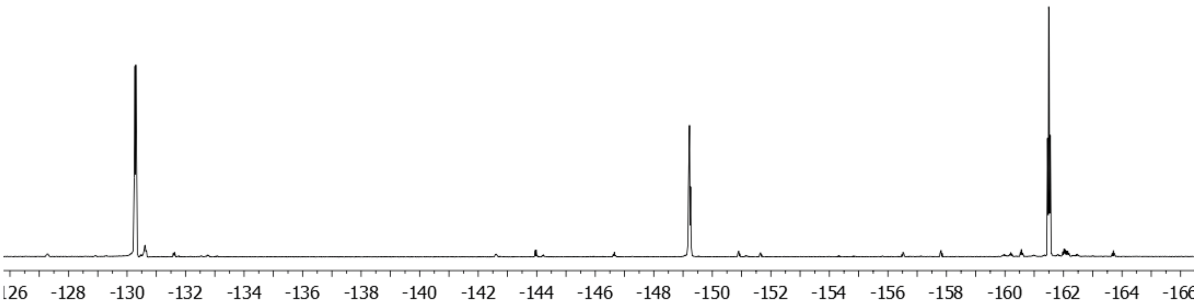

^{19}F NMR (564 MHz, 299 K, C_6D_6) spectrum of compound **10**.

b) After the NMR characterization of compound **10**, a second equivalent of $HB(C_6F_5)_2$ (34.5 mg, 0.10 mmol) was added to the clear yellow solution in the J Young NMR tube. The resulting reaction mixture was characterized by NMR experiments immediately.

Comment: A mixture of compounds **6b** : *trans*-**9** : *cis*-**9** [ca. 59 : 5 : 36 (^{1}H)] was detected by ^{1}H NMR spectroscopy. After 30 min. at r.t. a mixture of *trans*-**9** : *cis*-**9** [ca. 12 : 88 (^{1}H)] was observed.

Compound 6b (tentative assignment):

^{1}H NMR (600 MHz, 299 K, C_6D_6): δ 3.76 (1H), 1.99 (2H), 0.56 (1H), 0.36 (2H), -0.12 (2H).

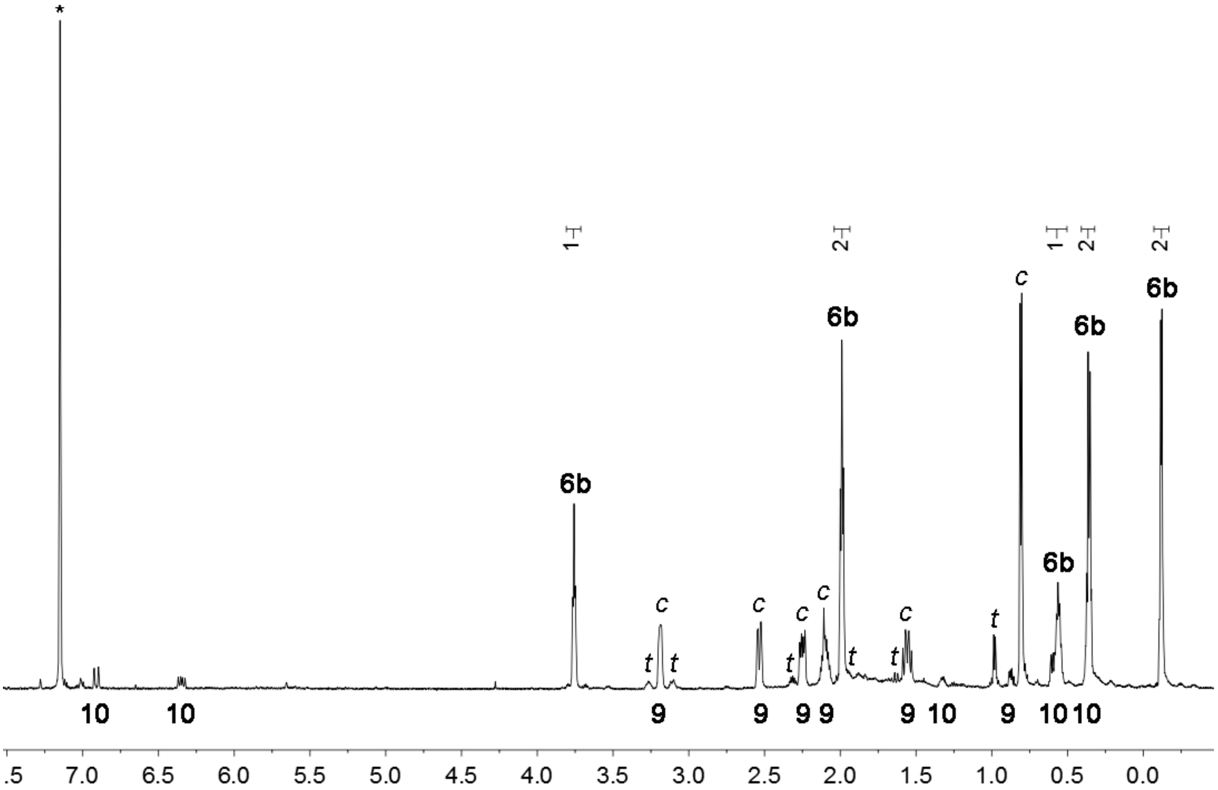

^{1}H NMR (600 MHz, 299 K, C_6D_6) spectrum immediately (ca. 5 min) after the addition of $HB(C_6F_5)_2$ to the reaction mixture of the hydroboration of **5b** (generation of compound **10**).*

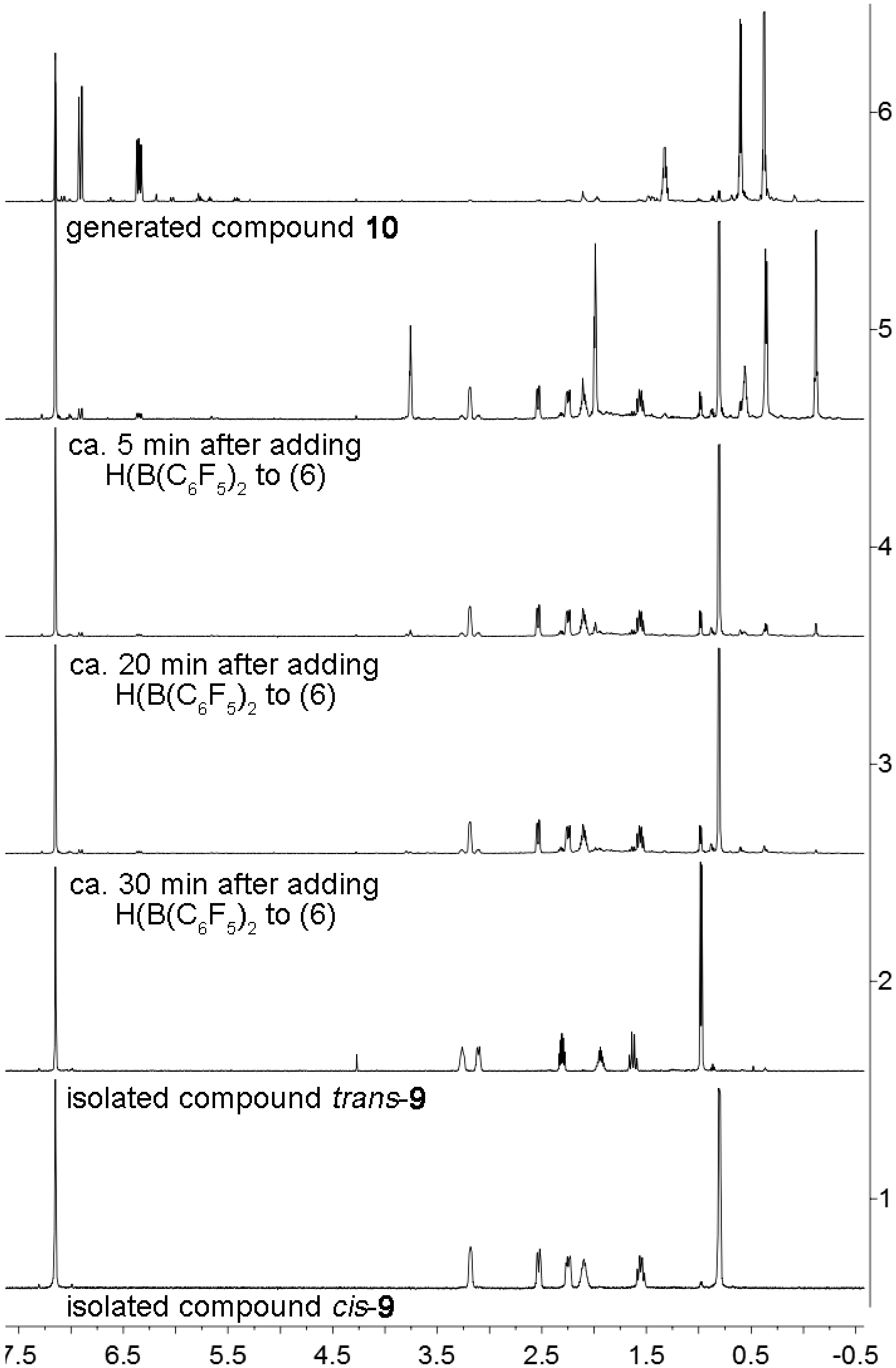

*^{1}H NMR (500 MHz, 299 K, C_6D_6) spectra of (1) isolated compound cis-**9**. (admixed with trans-**9**, see above) and (2) isolated compound trans-**9** (see above); ^{1}H NMR (600 MHz, 299 K, C_6D_6) spectra of (3) to (5) the addition of a second equivalent of $HB(C_6F_5)_2$ to the reaction mixture of compound **10** (see spectrum (6) and see 1st Experiment, part b) and (6) the reaction mixture of the generation of compound **10** (see 1st Experiment, part a).*

2nd Experiment: *Hydroboration of* 2-methyl-1-buten-3-yne ***(5c)*** *with* $HB(C_6F_5)_2$

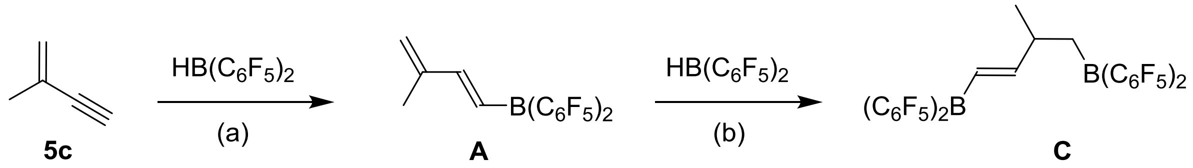

a) 2-Methyl-1-buten-3-yne (**5c**, 6.6 mg, 0.10 mmol) in C_6D_6 (0.5 mL) was added to a suspension of $HB(C_6F_5)_2$ (34.5 mg, 0.10 mmol) and C_6D_6 (0.3 mL) in a J Young NMR tube. After shaking for 1 minute at room temperature, the obtained clear yellow solution was characterized by NMR experiments immediately.

[The NMR data of compound **A** were consistent with those reported in the literature: P. Moquist, G.-Q. Chen, C. Mück-Lichtenfeld, K. Bussmann, C. G. Daniliuc, G. Kehr and G. Erker *Chem. Sci.*, **2015**, *6*, 816.]

Compound A:

^{1}H NMR (500 MHz, 299 K, C_6D_6): δ: 7.21, 6.91 (each d, $^3J_{HH}$ = 17.4 Hz, each 1H, HC=CH), 5.14, 5.11 (each m, each 1H, $=CH_2$), 1.70 (m, 3H, CH_3).

^{19}F NMR (470 MHz, 299 K, C_6D_6): -129.9 (m, 2F, *o*-C_6F_5), -148.4 (t, $^3J_{FF}$ = 20.9 Hz, 1F, *p*-C_6F_5), -161.3 (m, 2F, *m*-C_6F_5), [$\Delta\delta^{19}F_{m,p}$ = 12.9].

^{11}B{^{1}H} NMR (160 MHz, 299 K, C_6D_6): δ: 59.0 ($\nu_{1/2}$ ~ 700 Hz).

^{11}B NMR (160 MHz, 299 K, C_6D_6): δ: 59.0 ($\nu_{1/2}$ ~ 700 Hz).

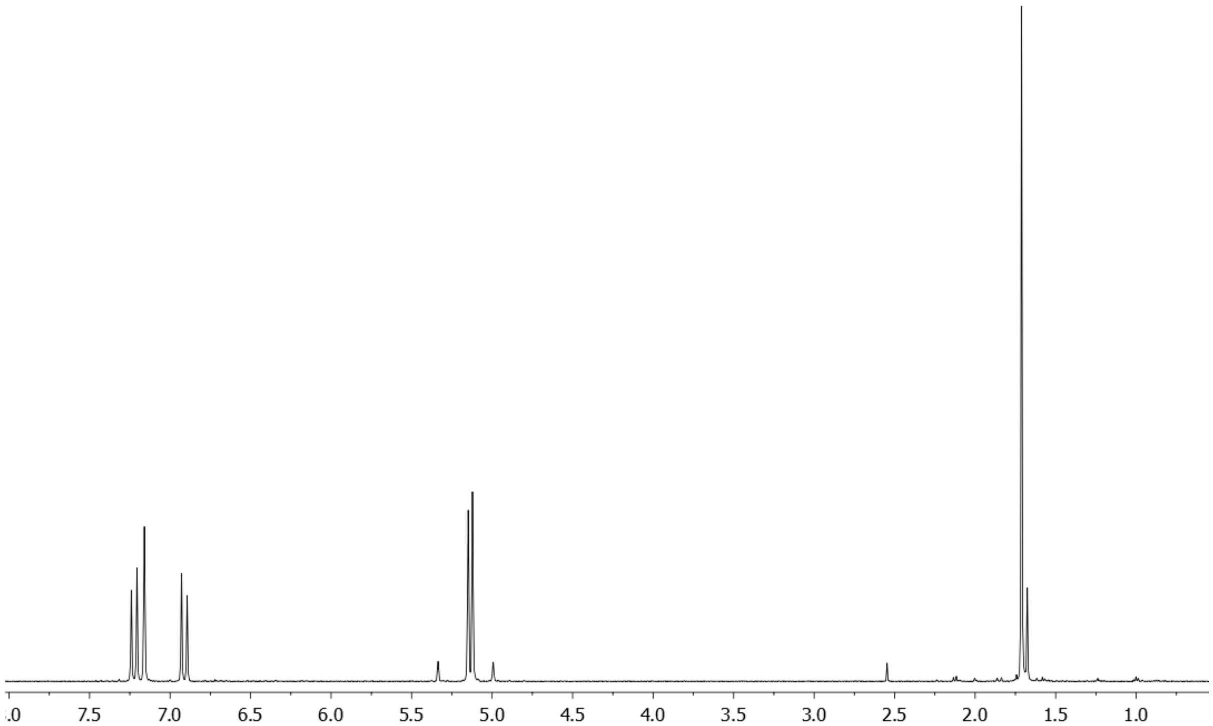

^{1}H NMR (500 MHz, 299 K, C_6D_6*) spectrum of compound **A**.*

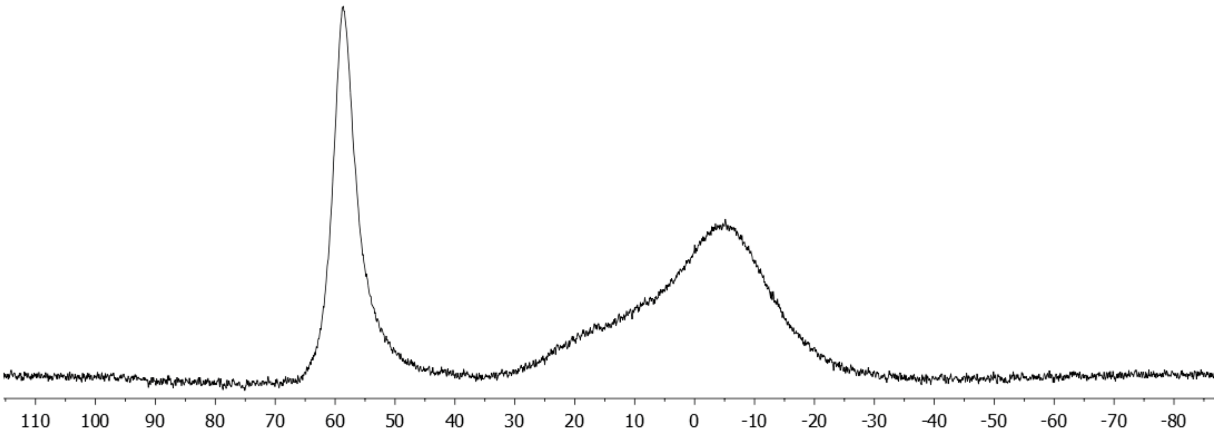

$^{11}B\{^1H\}$ NMR (160 MHz, 299 K, C_6D_6*) spectrum of compound* ***A****.*

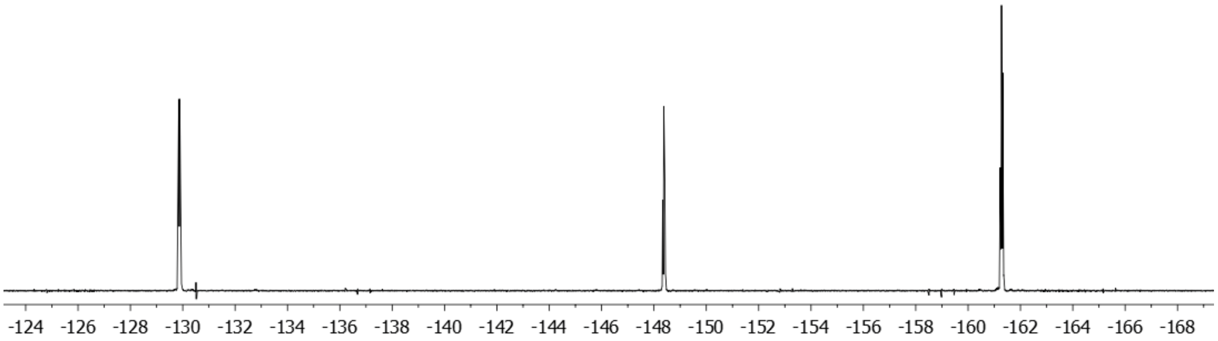

^{19}F NMR (470 MHz, 299 K, C_6D_6*) spectrum of compound* ***A****.*

b) After the NMR characterization of compound **A**, a second equivalent of $HB(C_6F_5)_2$ (34.5 mg, 0.10 mmol) was added to the clear yellow solution in the J Young NMR tube. The resulting reaction mixture was characterized by NMR experiments immediately.

Compound C:

^{1}H NMR (500 MHz, 299 K, C_6D_6): δ: 6.80 (dd, $^3J_{HH}$ = 17.3 Hz, $^3J_{HH}$ = 7.0 Hz, 1H, =CH), 6.69 (d, $^3J_{HH}$ = 17.3 Hz, 1H, BCH), 2.74 (m, 1H, CH), 2.09, 1.89 (each m, each 1H, BCH_2), 0.99 (d, $^3J_{HH}$ = 6.7 Hz, 3H, CH_3).

^{13}C{^{1}H} NMR (126 MHz, 299 K, C_6D_6): δ: 175.1 (=CH), 133.0 (br, BCH), 39.6 (CH), 38.2 (br, CH_2), 22.3 (CH_3), [C_6F_5 not listed].

^{19}F NMR (470 MHz, 299 K, C_6D_6): -130.0 (m, 2F, *o*), -147.5 (tt, $^3J_{FF}$ = 20.8 Hz, $^4J_{FF}$ = 4.8 Hz, 1F, *p*), -161.2 (m, 2F, *m*)(C_6F_5)[$\Delta\delta^{19}F_{m,p}$ = 13.7]; -130.4 (m, 2F, *o*), -145.9 (tt, $^3J_{FF}$ = 20.9 Hz, $^4J_{FF}$ = 3.6 Hz, 1F, *p*), -160.5 (m, 2F, *m*)(C_6F_5)[$\Delta\delta\ ^{19}F_{m,p}$ = 14.6].

^{11}B{^{1}H} NMR (160 MHz, 299 K, C_6D_6): δ: 72.9 ($\nu_{1/2}$ ~ 1500 Hz), 59.1 ($\nu_{1/2}$ ~ 1500 Hz).

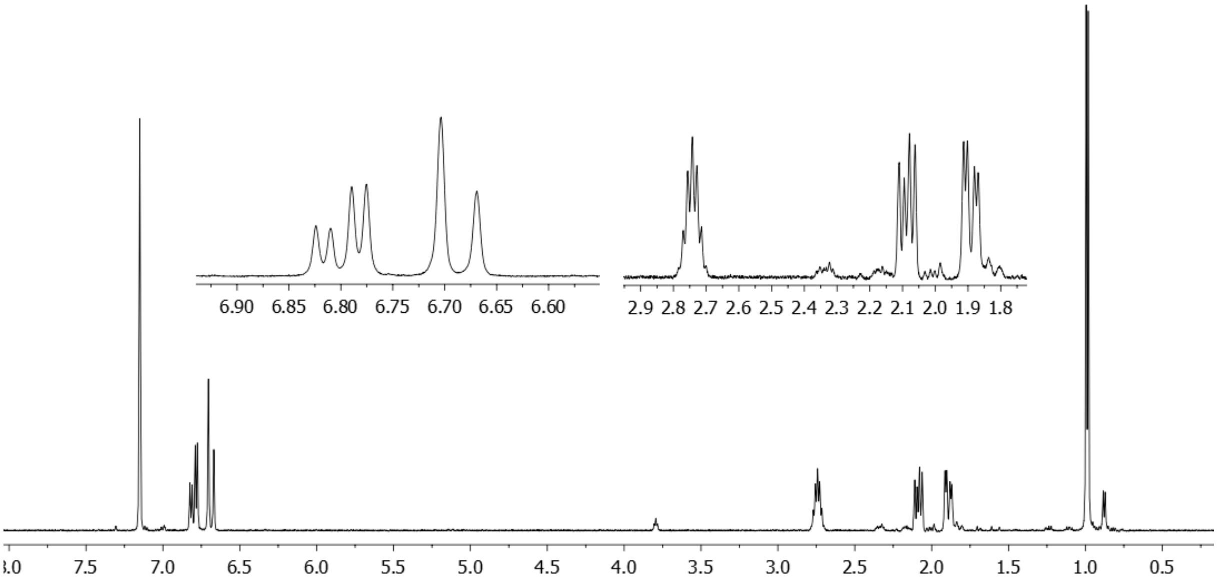

^1H NMR (500 MHz, 299 K, C_6D_6*) spectrum of compound* ***C****.*

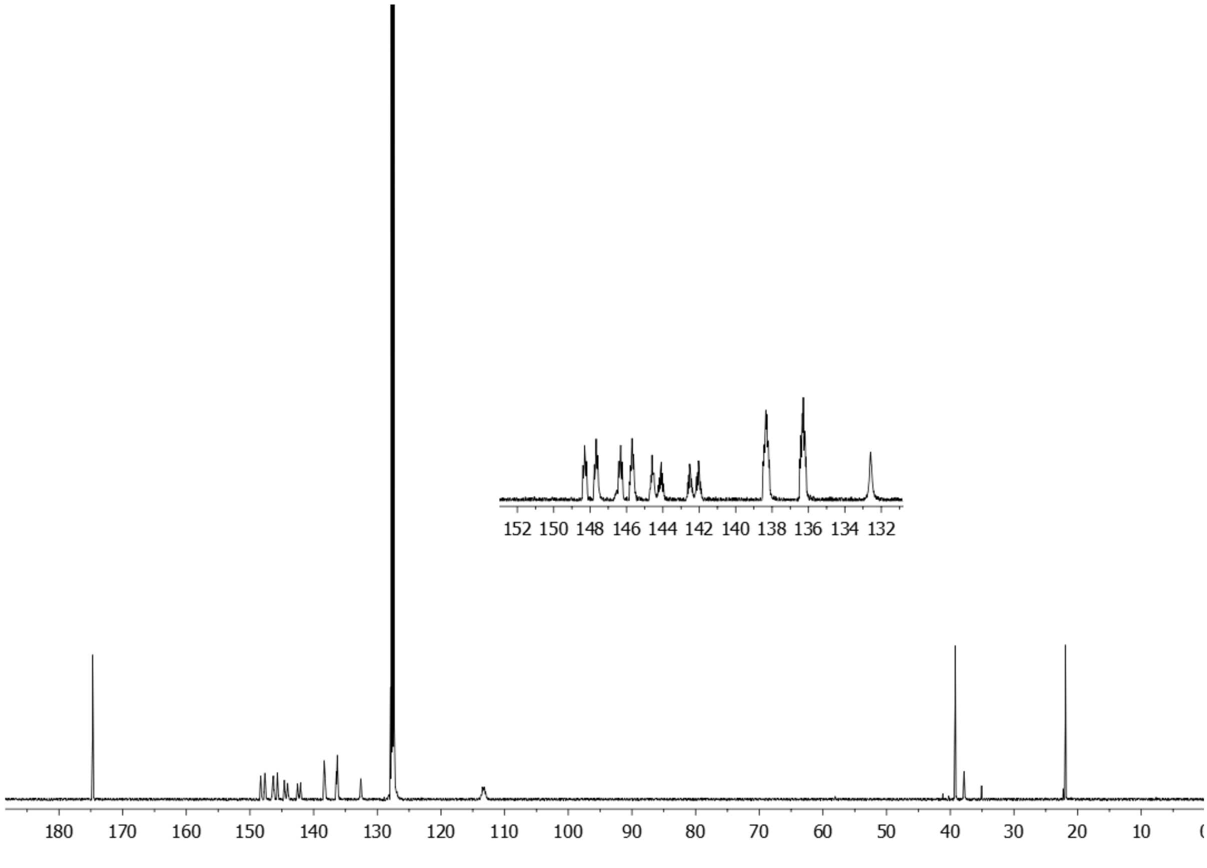

$^{13}C\{^1H\}$ NMR (125 MHz, 299 K, C_6D_6*) spectrum of compound* ***C****.*

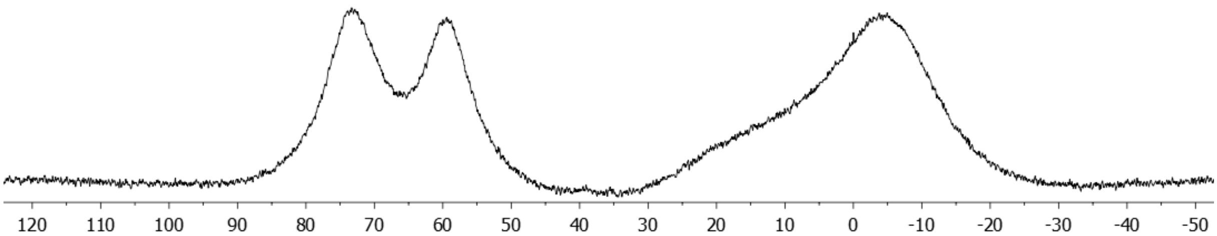

$^{11}B\{^1H\}$ NMR (192 MHz, 299 K, C_6D_6*) spectrum of compound* ***C****.*

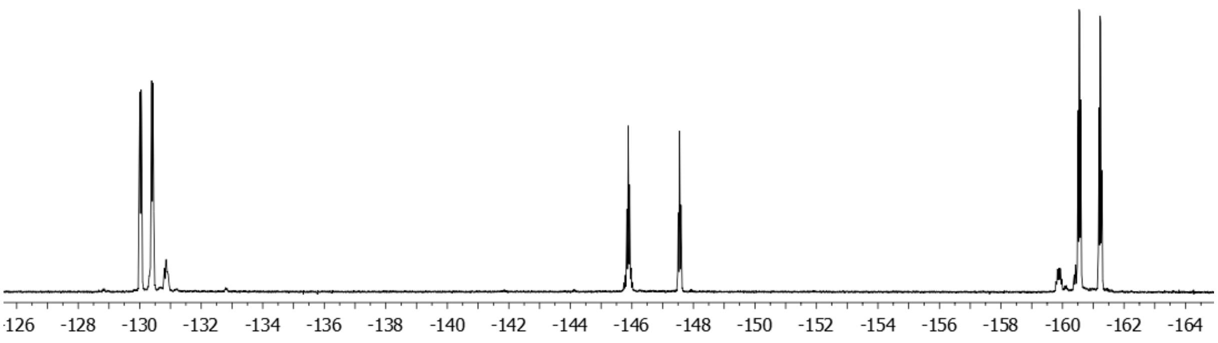

^{19}F NMR (470 MHz, 299 K, C_6D_6*) spectrum of compound **C**.*
